# The refinement of repeated exact time-to-event modeling: methodological refinements and implementation in NONMEM^®^ —part I

**DOI:** 10.3389/fphar.2026.1791423

**Published:** 2026-09-14

**Authors:** Jiyoung Seo, Hyeong-Seok Lim

**Affiliations:** Asan Medical Center, Department of Clinical Pharmacology and Therapeutics, University of Ulsan College of Medicine, Seoul, Republic of Korea

**Keywords:** adverse events, kaplan-meier, NONMEM^®^, NONMEM control stream, pharmacometrics, recurrent events, repeated time-to-event (RTTE), survival analysis

## Abstract

**Background:**

Parametric time-to-event (TTE) modeling is widely used in clinical trials to evaluate treatment response, patient prognosis, and drug-related adverse events (AEs). As follow-up assessments increasingly capture recurrent AEs, there is a growing need for analytical approaches that extend beyond traditional single-event frameworks. However, commonly used TTE models often assume independence between recurrent events, limiting their ability to represent inter-event dependence observed in real-world clinical data.

**Methods:**

We mathematically examined and reconstructed the existing NONMEM® (version 7.5) control code used for parametric repeated-event analyses and evaluated the statistical foundations of commonly applied parametric and nonparametric approaches, including Kaplan–Meier–based methods. Based on these analyses, we identified methodological gaps and proposed an improved probabilistic framework that more rigorously represents recurrent event dynamics while allowing for multiple dependent event occurrences. The proposed and existing methods were compared through simulation studies under diverse event-generation scenarios, and practical guidance for model selection was developed.

**Results:**

Three approaches were evaluated: two conventional parametric methods (methods 1, 2) and a newly proposed method (method 3). Overall, when the hazard function remained constant, all three methods produced similar results; however, clear differences in performance emerged depending on hazard dynamics and event density. When the hazard decreased gradually and events were relatively evenly distributed, method 2 demonstrated the closest alignment with nonparametric estimates, whereas when the hazard declined rapidly and events were concentrated in the early phase, method 3 showed a relatively better fit. In addition, because nonparametric estimators inherently involve uncertainty, discrepancies between parametric and nonparametric approaches do not necessarily indicate model misspecification, underscoring the need for careful interpretation in light of data characteristics and underlying hazard structures.

**Conclusion:**

These findings highlight the importance of selecting analytical methods according to data characteristics and suggest that complementary use of both widely used and newly proposed methods can improve the accuracy of recurrent event modeling. The proposed guidelines are expected to facilitate more systematic and reliable interpretation of recurrent AE data frequently encountered in clinical trials and real-world clinical settings, ultimately contributing to more robust evaluations of drug efficacy and safety as well as optimization of dosing strategies.

## 1 Introduction

Parametric time-to-event (TTE) modeling is widely used in analyses of clinical trial data to evaluate treatment responses, patient prognoses, and drug-related adverse events (AEs) (Collett, 2015; Lim, 2021; Bonate, 2011). As post-treatment follow-up assessments for the recurrence of AEs increase, there is a growing need for methodologies that can be extended beyond single-event analyses to accommodate recurrent events (Cook, 2007; Amorim and Cai, 2015; Ghosh and Lin, 2000; Rauch et al., 2018).

Currently, the TTE framework used for survival analysis in clinical trials was originally developed for single event analyses (Collett, 2015). Therefore, when a single TTE framework is applied to repeated TTE data, conventional approaches typically treat each event as independent (Cook, 2007). Hence, in such conventional approaches based on a single TTE framework, the likelihood of repeated events is computed as the product of individual event probabilities, assuming that the intervals between successive events follow independent single-event processes (Kaplan and Meier, 1958).

However, in real repeated event processes, events often exhibit dependence on previous events (Wang and Chang, 1999). Therefore, such dependence structures can be more appropriately described using conditional probability formulations (Andersen et al., 1993; Fleming and Harrington, 2011). Additionally, even when conditional probabilities are introduced, careful specification is required. If only one of the survival and hazard functions used in parametric methods is defined conditionally, the model may not adequately account for the possibility of multiple events occurring after the previous event and may implicitly assume that only a single event can occur in each interval. In such cases, the probability structure of the repeated event process is not rigorously defined, which may result in a likelihood that is not probabilistically valid.

To better understand these issues, this study conducted a mathematical investigation of the NONMEM® control stream used for parametric repeated time-to-event modeling implemented in NONMEM® (Version 7.5). The NONMEM control stream is a script-based model specification used in NONMEM, a standard software package for nonlinear mixed-effects modeling, to define model structures, parameters, statistical models, and estimation procedures.

In this study, we evaluated two conventional parametric approaches (methods 1 and 2) widely used in repeated time-to-event modeling. Both approaches treat the time interval between successive events as a single time-to-event process and apply this formulation repeatedly; however, they differ in the choice of time scale and in how dependence between events is handled. Method 1 uses gap time as the time scale and does not account for inter-event dependence, whereas method 2 uses calendar time and defines the survival function based on conditional probabilities, thereby partially reflecting dependence between events. To address these limitations, we propose a refined parametric approach (method 3) that uses calendar time and defines both survival and hazard functions within a conditional probability framework. To this end, we reconstructed the equations used in conventional parametric approaches and analyzed them from a probabilistic perspective, compared the likelihood definitions across the three approaches, and evaluated their relationships. Furthermore, we compared the three approaches in terms of probabilistic structure and practical implementation and provided practical guidance for selecting appropriate methods under different scenarios.

For the purposes of this study, we consider two types of repeated time-to-event (TTE) data: exact event times, where the precise timing of each event is recorded, and interval-censored events, where events are known to occur within specific time windows. While other data structures such as terminal events or competing risks also exist, these require different modeling frameworks (e.g., joint models or competing risk models) and are therefore not considered in this study. This paper focuses on parametric repeated time-to-event models with exact event times, while methodological approaches for interval-censored events will be presented separately in Part II.

## 2 Methods

### 2.1 Non-parametric analysis

#### 2.1.1 Kaplan–Meier framework for recurrent event data

To compare parametric approaches for recurrent events, we first considered a nonparametric approach (Kaplan–Meier (KM) estimator) as a reference framework for evaluation.

The mathematical expressions derived for the single-event scenario are summarized in Supplementary Files 1, 2 (Collett, 2015; Kalbfleisch and Prentice, 2002; Kleinbaum and Klein, 2012). The hazard and cumulative hazard functions of commonly used parametric survival models (exponential, Weibull, and log-logistic) are in Supplementary File 3 (Collett, 2015; Lim, 2021).

The KM estimator is a nonparametric method traditionally used to analyze single-event TTE data. The KM estimator assumes that the number of subjects not experiencing an event within the risk set at each event time follows a binomial distribution, with risk sets redefined at each event time point and initially defined as the total number of subjects at a baseline time point (typically $t_{0}=0$ ). A summary table of the KM estimator applied to a single TTE dataset is provided in Supplementary File 4 (Collett, 2015; Kaplan and Meier, 1958; Nelson, 1982; D’Arrigo et al., 2021; Greenwood, 1926).

In the analysis of repeated events with exact event times, a widely adopted nonparametric approach extends the KM framework originally developed for the single TTE analysis (Nelson, 1982). Each interval, defined between a previous event time and the subsequent event time, is reset using the previous event time as the baseline origin, with the occurrence of an event within that interval modeled as a separate binomial trial (Kaplan and Meier, 1958; Lawless, 2003; Therneau, 2023a; Therneau, 2023b; Therneau and Grambsch, 2000).

Accordingly, the probabilities of each event occurrence and non-occurrence within an interval sum to 1, and because the probability of event occurrence after the previous event time is calculated conditionally and multiplied across intervals, the event contribution from each interval is represented by a conditional probability. Although these intervals are not fully independent, the event contributions from each interval are treated as conditionally independent (Cook, 2007; Andersen et al., 1993; Therneau, 2023a; Therneau, 2023b; Therneau and Grambsch, 2000). The overall probability is then constructed as the product of these conditional probabilities, yielding the product-limit estimator of the survival function. As this does not constitute an exact joint distribution, this estimator should not be interpreted as a probability curve representing the exact joint distribution (Cook, 2007; Andersen et al., 1993; Therneau, 2023a; Therneau, 2023b; Therneau and Grambsch, 2000).

From a survival perspective, the probability of survival up to the previous event is treated as a realized constant and incorporated as a conditional component. Thus, because the survival probability up to the current time point is divided by that up to the prior time point, the resulting function can be regarded as conditional survival (Cook, 2007; Wang and Chang, 1999; Andersen et al., 1993; Lawless, 2003). In contrast, for the cumulative hazard, the baseline is reset to the preceding event time, effectively treating the hazard accumulated before that point as zero, and recalculating the cumulative hazard up to the current event time. Consequently, the cumulative hazard until the previous event is not retained. Thus, the KM method does not capture the conditional hazard but instead represents the event hazard in an unconditional form (Cook, 2007; Wang and Chang, 1999; Andersen et al., 1993; Lawless, 2003). Hence, the KM estimator resembles a conditional probability but does not constitute a true conditional distribution that reflects event-to-event dependence. In settings where such a dependence is present, the resetting mechanism fails to capture cumulative risk from earlier events, potentially leading to the underprediction or distortion of overall hazard dynamics (Cook, 2007; Wang and Chang, 1999; Andersen et al., 1993; Lawless, 2003).

Nonetheless, because of its simplicity and its ability to effectively capture overall trends in repeated-event processes, this nonparametric framework remains one of the most widely applied approaches in practice, particularly for visualization and model qualification using KM-type plots (Wang and Chang, 1999; Therneau, 2023a; Therneau, 2023b; Therneau and Grambsch, 2000). In the KM-based framework for repeated exact-time events, each event is treated as a distinct observation and ordered sequentially in time, irrespective of subject identifiers. This sequential arrangement defines the time intervals, with each interval representing the duration between successive events. Therefore, rather than estimating the conditional probability of an event occurring given the previous event for a specific individual, this approach focuses solely on the chronological occurrence of events across the total population, including all individuals (Cook, 2007; Wang and Chang, 1999; Andersen et al., 1993; Therneau, 2023a; Therneau, 2023b; Therneau and Grambsch, 2000).

#### 2.1.2 Parametric extensions corresponding to the KM recurrent event framework

In the nonparametric (KM-based) recurrent-event framework, the time origin is reset at the beginning of each inter-event interval, and event occurrence after the previous event time $t_{i}$ is modeled conditionally. Under this structure, $t_{i}$ is treated as an observed value (realized constant), and the random variable is defined only for the next event time $t_{i+1}$ (Cook, 2007; Wang and Chang, 1999; Andersen et al., 1993; Lawless, 2003). This conditional probability structure was also used to extend single-event parametric models to recurrent event settings. Table 1 summarizes the functions defined under this extended framework and compares them with the corresponding KM-based nonparametric estimates.

**TABLE 1:** Comparison of non-parametric (KM-based) and parametric extension of single-event TTE models for recurrent event models.

Quantity	Non-parametric extension (KM-estimates)	Parametric extension of single-event TTE models	Event process after $t_{i}$	Event interpretation	Probabilistic interpretation
Cumulative hazard^a^ (Nelson, 1982)	$\Delta H_{i}=\frac{e_{i+1}}{n_{i+1}}=\frac{e_{i+1}}{n_{i}-c_{i}}$	$\Delta H_{i}=H\left(t_{i},t_{i+1}\right)$	Multiple event occurrences possible	Mean number of events	Expected number of events in the interval $\left(t_{i},t_{i+1}\right]$
Survival (Kleinbaum and Klein, 2012; Lawless, 2003)	$\hat{P}\left(T>\;t_{i+1}\left|T\right.>\;t_{i}\right)$ $=\hat{S\left(t_{i},t_{i+1}\right)}=1-\Delta H_{i}$	$P\left(T>\;t_{i+1}\left|T>\;\right.t_{i}\right)$ $=S\left(t_{i},t_{i+1}\right)$ $=\exp^{-H\left(t_{i},t_{i+1}\right)}$	Multiple event occurrences possible	KM: no event	Cumulative probability of no event occurrence in the interval $\left(t_{i},t_{i+1}\right]$
				Parametric: first event	Cumulative probability that the first event does not occur in the interval $\left(t_{i},t_{i+1}\right]$
Conditional cumulative (density) probability^b^ (Cook, 2007; Andersen et al., 1993; Kleinbaum and Klein, 2012; Lawless, 2003)	$\hat{P}\left(t_{i}<T\leq t_{i+1}\left|T\right.>\;t_{i}\right)$ $=\hat{F\left(t_{i},t_{i+1}\right)}$ $=1-\hat{S\left(t_{i},t_{i+1}\right)}=\Delta H_{i}$	$P\left(t_{i}<T\leq t_{i+1}\left|T>\;\right.t_{i}\right)$ $=F\left(t_{i},t_{i+1}\right)$ $=1-S\left(t_{i},t_{i+1}\right)$	Multiple event occurrences possible	KM: ≥1 event	Cumulative probability that at least one event occurs in the interval $\left(t_{i},t_{i+1}\right]$
				Parametric: first event	Cumulative probability that the first event occurs in the interval $\left(t_{i},t_{i+1}\right]$
Conditional (density) probability^c^ (Cook, 2007; Andersen et al., 1993; Fleming and Harrington, 2011)	$\hat{P}\left(T=t_{i+1}|T>t_{i}\right)$ $=\hat{f\left(t_{i+1}|t_{i}\right)}=\Delta H_{i}$	$P\left(T=t_{i+1}|T>t_{i}\right)$ $=f\left(t_{i+1}|t_{i}\right)=h\left(t_{i+1}\right)\cdot S\left(t_{i+1}|t_{i}\right)$	KM: Multiple event occurrences possible	KM: ≥1 event	Conditional probability of event occurrence at $t_{i+1}$ given no event occurred in $\left(t_{i},t_{i+1}\right)$
			Parametric: Only one event occurrence possible	Parametric: Only one event occurrence possible	Conditional density of a single event occurrence at $t_{i+1}$ given no event occurred in $\left(t_{i},t_{i+1}\right)$

Given these conceptual distinctions, to avoid confusion with the symbols used in the parametric context, we denoted both the survival function and the associated probabilities in the nonparametric method using a $\hat{hat\;}$ notation.

^a^ Where $e_{i+1}$ is the number of events at $t_{i+1}$ , $n_{i}$ ​ is the number of subjects at risk just prior to $t_{i}$ , $n_{i+1}$ ​ is the number of subjects at risk just prior to $t_{i+1}$ and $c_{i}$ ​ is the number of censored subjects within the interval $(\left.t_{i},t_{i+1}\right]$ (Lawless, 2003). The cumulative hazard increment, between two successive events, reflects only the instantaneous risk of event occurrence at the current time point $t_{i+1}$ , not considering the cumulative hazard at the previous time point $t_{i}$ ​ (Andersen et al., 1993; Kleinbaum and Klein, 2012). Additionally, despite the absence of events between $t_{i+1}$ ​(excluding the event at $t_{i+1}$ ) and $t_{i+2}$ , the hazard is assumed to be constant over the interval [ $\left.t_{i+1},t_{i+2}\right)$ . Accordingly, this value is approximated and applied as the average hazard rate for that interval (Cook, 2007; Andersen et al., 1993).

^b^ The equivalence between the interval cumulative probability and the conditional cumulative probability is demonstrated in Supplementary File 5 (Kaplan and Meier, 1958; Wang and Chang, 1999).

^c^ The detailed mathematical derivation of Table 1 is provided in Supplementary File 5 (Cook, 2007; Andersen et al., 1993; Fleming and Harrington, 2011).

As shown in Table 1, the two approaches begin to diverge conceptually in the definition and interpretation of the survival function. In the non-parametric (KM-based) framework, the survival function ( S^ ), conditional cumulative probability ( F^ ), and conditional probability ( f^ ) are not restricted to a specific event such as the first event but instead represent probability structures that reflect the aggregate effect of multiple events that may occur after the previous event time *t*_i_. Consequently, these quantities can be interpreted as the probability that no event occurs in the interval (i.e., survival probability ( S^ )) and, complementarily, as the cumulative probability that at least one event occurs (i.e., conditional cumulative probability ( F^ )), thereby reflecting the possibility of multiple event occurrences after the previous event time *t*_i_.

In contrast, when single-event parametric models are extended to recurrent events using the same framework, although the possibility of multiple events after the previous event time $t_{i}$ is still allowed, the definitions of the survival function (*S*) and conditional cumulative probability (*F*) are interpreted differently. Specifically, the probability structure in each interval is interpreted in terms of the time to the first event occurring after *t*_i_, rather than the aggregate occurrence of multiple events.

This difference in the interpretation of the survival function (*S*) ultimately leads to a more substantial difference in the definition of the conditional probability density function (*f*). Unlike the survival function (*S*) or conditional cumulative probability function (*F*), the conditional probability density function (*f*) is defined under the assumption that only a single event occurs after the previous event time $t_{i}$ . Therefore, when only one event occurs after $t_{i}$ , the conditional probability density (*f*) is defined for the occurrence of that event at time $t_{i+1}$ . This differs from the non-parametric approach, which allows the possibility of multiple events after $t_{i}$ .

In summary, although single-event parametric models can be extended to recurrent event settings using the same framework applied in the KM-based approach, conceptual differences between the two approaches remain. Nevertheless, the parametric formulation presented in Table 1 can be regarded as the parametric approach most closely aligned with the KM estimates.

In the following sections, we introduce three parametric approaches (methods 1–3) that derive conditional probability density functions (*f*_*k*_) under different probabilistic assumptions for recurrent events, and we investigate in which situations each method is most appropriate. In addition, we compare these methods with the KM estimates and identify which parametric methods yield function structures similar to those shown in Table 1.

The function f introduced in Supplementary File 1 corresponds to the probability density function defined for single-event time-to-event models, and the same notation will be used throughout this paper. The conditional probability density functions derived in each parametric method (methods 1, 2, and 3) will be denoted by $f_{k}$ , and their relationship to the single-event density f will be described in the following sections.

The approach used for the repeated exact TTE analysis in the survival package in R (version 4.4.1) aligns with the methodology described above. The nonparametric analysis plot for the repeated event example presented later was also generated using the survival package in R (Therneau, 2023a; Therneau, 2023b; Therneau and Grambsch, 2000).

The KM-based cumulative hazard was compared with the parametric estimate of $-logS\left(t\right)$ , with detailed justification provided in Supplementary File 6 (Collett, 2015; Kaplan and Meier, 1958; OpenStax, 2022; Conrad, 2010).

### 2.2 Likelihood function

In parametric models for recurrent events, the conditional probability density function for each event, $f_{k}$ , constitutes a component of the likelihood function used in maximum likelihood estimation implemented in NONMEM®. Parameter estimation is performed by maximizing this likelihood function (Bauer et al., 2022).

The likelihood function for recurrent event data differs fundamentally from that for single-event data, primarily due to the dependence structure between successive events (Cook, 2007). In recurrent event data, the likelihood is expressed as the product of conditional probability density functions ( $\left.f_{k}\right)$ for successive event occurrences, where dependence between events is handled through conditioning on the previous event time. However, the specific formulation and modeling of this conditional structure differ across the methods presented later (methods 1, 2, and 3), and this constitutes the main difference between the parametric approaches.

If the kth subject experiences $n_{k}$ events, the likelihood for the kth subject is given as follows (Cook, 2007; Lavielle, 2014).

$$L_{k}=\left\{\prod_{i=0}^{n_{k}-1}f_{k}\left(t_{k,i+1}|t_{k,i}\right)\right\}\cdot S\left(t_{c_{k}}|t_{k,n_{k}}\right)$$ (1)

• $L_{k}$ : observed likelihood for the $k^{th}$ subject,

• $n_{k}$ : total number of exact time events for $k^{th}$ subject,

• $t_{k,i+1}$ : exact time at which the ${\left(i+1\right)}^{th}$ event occurs (event of interest),

• $t_{k,i}$ : exact time at which the $i^{th}$ event occurs (preceding event of interest),

• $t_{c_{k}}$ : time beyond which no further events are observed (right censoring),

• $S\left(t_{c_{k}}|t_{k,n_{k}}\right)$ : survival probability beyond the last observed event time, accounting for right-censoring,

• $f_{k}\left(t_{k,i+1}|t_{k,i}\right)$ : conditional probability density that the ${\left(i+1\right)}^{th}$ event occurs at $t_{k,i+1}$ , given that the $i^{th}$ event has occurred at $t_{k,i}$ , and no additional events have occurred during the interval $\left(t_{k,i},t_{k,i+1}\right)$

The formal likelihood function for repeated exact events is expressed in Equation 1. Each term in the likelihood represents the conditional probability density of the subsequent event occurring, given that no events have taken place between the time of the previous event and the current time point (Cook, 2007; Lavielle, 2014). Meanwhile, the final survival term accounts for right-censoring after the last observed event (Cook, 2007).

The overall likelihood function is constructed as the product of the individual likelihoods for each subject, assuming independence between subjects (Cook, 2007; Lavielle, 2014),

$$L=\prod_{k}L_{k}$$ (2)

For accurate estimation in parametric models for recurrent events, the likelihood function in Equation 2 must be properly defined. To achieve this, the survival and hazard functions whose product defines the conditional probability density function $f_{k}$ for each event must be clearly specified (Cook, 2007; Lavielle, 2014).

For notational simplicity, the subject index k is omitted from the event-time notation in the following sections, and event times are indexed only by the event order i .

### 2.3 Theoretical background and mathematical verification of parametric method

In this study, the mathematical formulations of methods 1, 2 were constructed based on the control stream code implemented in NONMEM® (Version 7.5), and the corresponding code is provided in Supplementary Files 7, 8 (Bauer et al., 2022).

We focused primarily on scenarios with decreasing hazard patterns and constant hazard conditions were also considered. In contrast, increasing hazard patterns are relatively uncommon in repeated event analyses and were therefore not included within the scope of this study (Amorim and Cai, 2015; Rauch et al., 2018).

#### 2.3.1 Method 1

##### 2.3.1.1 Time scale

In method 1, the conditional survival and hazard functions are not defined directly using the actual event times; instead, they are defined based on the time between successive events (gap time) (Cook, 2007; Kalbfleisch and Prentice, 2002).

##### 2.3.1.2 Conditional survival and hazard functions

When the gap time scale is applied to the survival framework, the conditional survival and hazard functions in method 1 are defined as functions of the elapsed time ( $\Delta t_{i}=t_{i+1}-t_{i}$ ) since the previous event, $t_{i}$ . In this framework, the time origin is reset at each event time, and the previous event time, $t_{i}$ is treated as a new time origin $t_{0}$ , at which the survival probability is equal to one. Consequently, the conditional survival function can be expressed in the same form as the unconditional survival function defined on the gap time scale, $\Delta t_{i}$ (Cook, 2007; Kalbfleisch and Prentice, 2002). The same time redefinition applies to the hazard function. As a result, the conditional hazard function simplifies to the unconditional instantaneous hazard function evaluated on the gap time scale, $\Delta t_{i}$ .

Accordingly, the conditional survival and hazard functions can be written as,

$$S\left(t_{i+1}|t_{i}\right)=P\left(T>\Delta t_{i}|T>t_{0}\right)=S\left(\Delta t_{i}\right)\;h\left(t_{i+1}|t_{i}\right)=\lim_{\delta t\to 0}\frac{P\left(\left(\Delta t_{i}\leq T<\Delta t_{i}\left.+\delta t\right|T>t_{0}\right)|\left(T\geq \Delta t_{i}|T>t_{0}\right)\right)}{\delta t}=h\left(\Delta t_{i}\right)$$ (3)

When time-shifted intervals are used instead of actual event times, only the initial segment of the underlying parametric survival function is repeatedly applied to each recurrent event. As a result, the survival and hazard functions defined in Equation 3 may differ from those defined on the absolute time scale. This modeling framework can therefore be interpreted as a renewal process, in which the event-generating process restarts after each event (Cook, 2007; Kalbfleisch and Prentice, 2002).

##### 2.3.1.3 Conditional probability density function

As defined in Section 2.2, the conditional probability density function $f_{k}\left(t_{k,i+1}|t_{k,i}\right)$ can be expressed as the product of the conditional survival function and the conditional hazard function. By defining the gap time $\Delta t_{i}$ as a realization of the random variable *T* and differentiating the cumulative distribution function $F\left(t\right)$ with respect to time t , the conditional probability density function can be obtained by substituting $t=\Delta t_{i}$ (Cook, 2007; Kalbfleisch and Prentice, 2002):

$$f_{k}\left(t_{i+1}|t_{i}\right)=S\left(\Delta t_{i}\right)\cdot h\left(\Delta t_{i}\right)=P\left(T=\Delta t_{i}\right)=\frac{d}{dt}F\left(t\right){|}_{t=\Delta t_{i}}=f\left(\Delta t_{i}\right)$$ (4)

Here, $f\left(\Delta t_{i}\right)$ presented in Equation 4 is the derivative of the cumulative distribution function $F\left(\Delta t_{i}\right)$ , where $F\left(\Delta t_{i}\right)$ represents the cumulative probability that the first event occurs by time $\Delta t_{i}$ . Therefore, $f\left(\Delta t_{i}\right)$ represents the probability density that a single event occurs at time $\Delta t_{i}$ under the assumption that only one event occurs after the reset time origin $t_{0}$ (Cook, 2007; Kalbfleisch and Prentice, 2002). Thus, in method 1, the conditional probability density function $f_{k}$ defined in the recurrent event framework is equivalent to the probability density function f of a single-event model defined in Supplementary File 1, applied directly on the gap time scale.

Because the probability density function for each event in method 1 does not depend on previous events, the probability structure of earlier events does not influence the distribution of subsequent events, and the previous event time only serves to reset the time origin. Consequently, inter-event times (gap times) are assumed to be independent and identically distributed (Cook, 2007; Kalbfleisch and Prentice, 2002).

As a result, the conditional probability density function $f_{k}\left(t_{i+1}|t_{i}\right)$ derived in Equation 4 does not explicitly account for the dependency structure inherent in recurrent events. For this reason, method 1 may not fully reflect the conditional event occurrence process following previous events and may additionally differ from the non-parametric KM-based approach, which accounts for the probability of multiple event occurrences after the previous event time.

The precise definitions of the RTTE functions used in method 1 and their detailed mathematical derivations are provided in Supplementary File 9.

##### 2.3.1.4 Comparison between method 1 and KM estimates

A comparison between the non-parametric KM estimates and method 1 is summarized in Table 2.

**TABLE 2:** Comparison between non-parametric KM-based and Method 1 approaches for recurrent events.

Quantity	Non-parametric extension (KM-estimates)	Method 1
Level of analysis	Population-level^a^	Individual level^b^
Time scale	Calendar time	Gap time
Conditional survival	Probability of no event occurrence in the interval $(\left.t_{i},t_{i+1}\right]$	Cumulative probability that the first event does not occur by gap time $\Delta t_{i}$
Conditional hazard	Instantaneous event occurrence rate at time $t_{i+1}$	Instantaneous event occurrence rate at gap time $\Delta t_{i}$
Conditional (density) Probability	Conditional probability of event occurrence at $t_{i+1}$ , given no event occurred in $\left(t_{i},t_{i+1}\right)$	Conditional density of a single event occurrence at gap time $\Delta t_{i}$ , given no event occurred in $\left(t_{0},\Delta t_{i}\right)$
Event process interpretation	Event count process (multiple events possible)	Renewal process (single event after $t_{i}$ , reset as $t_{0}$ )
Dependence structure	Conditional independence given previous event	Renewal independence (independent gap times)
History dependence	Dependence through survival function	No dependence on previous event history

Notation: $t_{i}$ denotes the previous event time, $t_{i+1}$ denotes the current event time, and $\Delta t_{i}=t_{i+1}-t_{i}$ represents the gap time between two consecutive events.

^a^ Interval-based aggregation of event information across time, irrespective of subject identity, where event information is aggregated at each event time across the entire population.

^b^ Sequential accumulation and representation of event information over time on a per-subject basis, through event-time-based modeling with time-ordered updating for each individual subject.

Conceptually, method 1 relies on a probabilistic structure that differs fundamentally from the KM-based recurrent event analysis. The KM approach estimates event occurrence probabilities based on aggregated event counts and corresponding risk sets at each event time across the entire population, without assuming a parametric form (Kaplan and Meier, 1958; Wang and Chang, 1999). In contrast, method 1 models event occurrence times at the individual level using probability density functions defined for each subject, with parameters estimated by processing each subject’s event history sequentially over time and updating the likelihood contribution at each event record. The resulting parameter estimates are obtained by aggregating these individual-level likelihood contributions across all subjects (Cook, 2007; Andersen et al., 1993).

This individual-level modeling framework contrasts with the KM approach, which operates at the population level by directly capturing event occurrence probabilities from observed data at each event time. Consequently, the probability structure reflecting multiple event occurrences after the previous event time, as captured by the KM method, may not be fully represented in method 1. As a result, method 1-based models may underestimate event occurrence probabilities relative to KM-based nonparametric estimates under certain conditions (Amorim and Cai, 2015; Andersen et al., 1993).

The two methods also differ in their interpretation of the event occurrence process. The KM approach assumes an event count process in which multiple events may occur after the previous event time, whereas method 1 assumes a renewal process structure in which only the next event after the previous event is modeled, based on the assumption that only one event occurs after each previous event, with the time origin reset to $t_{0}$ following each event (Cook, 2007; Kalbfleisch and Prentice, 2002).

The probabilistic dependency structures also differ between the two approaches. In the KM method, dependence between event occurrences is partially retained through the survival structure up to the previous event time, resulting in a conditional independence structure. In contrast, method 1 assumes renewal independence, where inter-event times (gap times) are independent and the probability structure of previous events or event history does not influence future event distributions (Cook, 2007; Kalbfleisch and Prentice, 2002).

These differences in probabilistic structure may lead to discrepancies between non-parametric KM estimates and simulation-based results obtained from parametric models using method 1, such as visual predictive checks (VPCs) (Cook, 2007; Amorim and Cai, 2015).

##### 2.3.1.5 Special case: equivalence between method 1 and KM estimates

In general, method 1 differs conceptually from the KM-based approach. However, when the hazard function is constant over time, the time-shift transformation does not alter the hazard value, and the two methods yield equivalent results.

In particular, under an exponential model with constant hazard, the hazard calculated using the gap time is identical to the hazard evaluated at the actual event time. Consequently, the survival and hazard functions obtained from method 1 coincide with the corresponding conditional quantities defined on the actual event time scale. A detailed proof of this equivalence is provided in Supplementary File 10 (Kalbfleisch and Prentice, 2002).

Therefore, although method 1 can produce results similar to those of the KM approach in the special case of constant hazard, differences may arise when the hazard varies over time due to the different probabilistic structures of the two methods. This discrepancy may lead to differences between simulation-based visual predictive checks (VPCs) from method 1 and KM-based non-parametric estimates. To address this limitation, an alternative approach is introduced in the following section (method 2).

#### 2.3.2 Method 2

##### 2.3.2.1 Time scale

Unlike method 1, method 2 does not apply a time shift when defining the conditional survival and hazard functions; instead, it uses calendar time based on the actual event times (Cook, 2007; Andersen et al., 1993; Fleming and Harrington, 2011).

##### 2.3.2.2 Conditional survival and hazard functions

In method 1, the previous event time $t_{i}$ is reset as a new time origin $t_{0}$ , which effectively removes the probabilistic structure prior to the previous event. In contrast, method 2 maintains the previous event time $t_{i}$ as the conditioning time and directly incorporates survival information up to that time into the conditional probability structure (Andersen et al., 1993; Fleming and Harrington, 2011). In this setting, the survival probability up to $t_{i}$ is treated as a realized quantity rather than a random variable and thus serves as conditioning information for defining the distribution of subsequent event times. Consequently, the current event time $t_{i+1}$ is defined under a conditional probability structure given survival up to the previous event time $t_{i}$ (Cook, 2007; Andersen et al., 1993; Fleming and Harrington, 2011).

Within this conditional framework, the joint survival distribution can be decomposed by conditioning on the previous event time $t_{i}$ (Cook, 2007; Kalbfleisch and Prentice, 2002; Lavielle, 2014). The conditional survival function is therefore defined as

$$S\left(t_{i+1}|t_{i}\right)=P\left(T>t_{i+1}|T>t_{i}\right)=S\left(t_{i},t_{i+1}\right)$$ (5)

The conditional survival function defined in Equation 5 can be interpreted as the survival function for a single event conditional on the previous event time $t_{i}$ and thus represents a conditional form of the first-event survival function. This formulation therefore reflects a probabilistic structure that preserves the dependency inherent in recurrent event processes.

The conditional hazard function can be defined in a manner analogous to the conditional survival function by treating the cumulative hazard up to the previous event time $t_{i}$ as a realized quantity. In general, the hazard function $h\left(t\right)$ is defined as the derivative of the cumulative hazard function $H\left(t\right)$ (Cook, 2007; Kalbfleisch and Prentice, 2002). Accordingly, the conditional hazard function $h\left(t_{i+1}|t_{i}\right)$ can be expressed as the derivative of the conditional cumulative hazard function $H\left(t_{i+1}|t_{i}\right)$ (Cook, 2007; Andersen et al., 1993; Fleming and Harrington, 2011; Aalen et al., 2008). Since the conditional cumulative hazard $H\left(t_{i+1}|t_{i}\right)$ represents the cumulative hazard accumulated over the interval $\left(t_{i},t_{i+1}\right]$ (Supplementary File 11), the conditional hazard function corresponds to the derivative of the cumulative hazard over that interval (Andersen et al., 1993; Fleming and Harrington, 2011; Kleinbaum and Klein, 2012; Rudin, 1976; Apostol, 1974).

In method 2, sequential conditioning is applied in the probabilistic formulation, and therefore the cumulative hazard up to the previous event time $t_{i}$ is first treated as a random variable during the derivation. However, once the previous event time has been realized, the cumulative hazard up to $t_{i}$ becomes a fixed quantity and is subsequently treated as constant when differentiation is performed at the current event time $t_{i+1}$ . As a result, the cumulative hazard term prior to the previous event is eliminated in the differentiation process, and the conditional hazard function is simplified to the instantaneous hazard at the current event time $t_{i+1}$ (Andersen et al., 1993; Fleming and Harrington, 2011).

$$h\left(t_{i+1}|t_{i}\right)=H^{\prime }\left(t_{i+1}|t_{i}\right)=H\prime \left(t_{i},t_{i+1}\right)=\frac{d}{dt}{\left[H\left(t\right)-H\left(t_{i}\right)\right]}_{t=t_{i+1}}=h\left(t_{i+1}\right)$$ (6)

Consequently, although the conditional survival function reflects the cumulative survival structure up to the previous event time $t_{i}$ , the conditional hazard function is expressed as the instantaneous hazard at the current time $t_{i+1}$ (as defined in Equation 6), and thus the carryover effect of cumulative hazard is not preserved at the hazard level. In other words, dependency between previous events is reflected in the survival probability structure but not reflected at the instantaneous hazard level.

##### 2.3.2.3 Conditional density function

In method 2, the conditional probability density $f_{k}\left(t_{i+1}|t_{i}\right)$ is defined based on a sequential conditional probability structure according to the event order. The survival probability and cumulative hazard up to the previous event time $t_{i}$ are treated as random variables during the derivation but become fixed quantities once the previous event time $t_{i}$ has been observed. Therefore, the conditional cumulative distribution function $F\left(t|t_{i}\right)$ for the current event time $t_{i+1}$ is continuous and differentiable with respect to t . Differentiation with respect to t followed by evaluation at $t=t_{i+1}$ yields the conditional probability density function $f_{k}\left(t_{i+1}|t_{i}\right)$ (Andersen et al., 1993; Fleming and Harrington, 2011; Aalen et al., 2008).

$$f_{k}\left(t_{i+1}|t_{i}\right)=S\left(t_{i+1}|t_{i}\right)\cdot h\left(t_{i+1}\right)=\frac{d}{dt}P\left(T\leq t|T>t_{i}\right){|}_{t=t_{i+1}}=\frac{d}{dt}F\left(t|t_{i}\right){|}_{t=t_{i+1}}=f\left(t_{i+1}|t_{i}\right)$$ (7)

The conditional probability density $f_{k}\left(t_{i+1}|t_{i}\right)$ defined over the interval $\left(t_{i},t_{i+1}\right]$ in Equation 7 is structurally equivalent to the probability density function $f\left(t|t_{0}\right)$ for a single time-to-event model defined in Supplementary File 1 (Collett, 2015; Kalbfleisch and Prentice, 2002; Kleinbaum and Klein, 2012). The difference between the two formulations lies in the treatment of the reference time. In Supplementary File 1, the reference time $t_{0}$ is treated as a realized constant and the survival probability is set to $S\left(t_{0}\right)=1$ , whereas in method 2 the reference time $t_{i}$ is treated as a random variable during the probabilistic formulation but, once the event time has been observed, the realized survival value $S\left(t_{i}\right)$ is treated as a constant and incorporated into the conditional probability structure (Collett, 2015; Kalbfleisch and Prentice, 2002; Kleinbaum and Klein, 2012). Accordingly, the conditional density in method 2 may be interpreted as the corresponding form of the single-event density defined in Supplementary File 1.

In method 2, the probability density for each event depends partially on the previous event time $t_{i}$ . Survival information up to the previous event time $t_{i}$ is retained as conditioning information within the conditional framework, whereas the probability update at the current event time $t_{i+1}$ is determined by the instantaneous hazard at that time. Consequently, the time between successive events is defined under a conditional structure given the previous event time $t_{i}$ and is assumed to follow the same functional form of conditional distribution (Cook, 2007; Andersen et al., 1993; Fleming and Harrington, 2011).

However, under this updating structure, potential cumulative effects from previous events (carryover effects) are not directly reflected at the hazard level; rather, such effects are incorporated indirectly through the conditional survival structure (Cook, 2007; Andersen et al., 1993).

The probability density function defined in method 2 leads to a likelihood structure that is structurally similar to the nonparametric approach obtained by extending the Kaplan–Meier estimator to recurrent event settings, as presented in Table 1. In method 2, the likelihood is constructed sequentially through conditional probabilities for successive event occurrences, which makes it closely related to KM-based approaches. However, despite this structural similarity, the conditional density $f\hat{\left(t_{i+1}|t_{i}\right)}$ in the nonparametric approach and the conditional density $f_{k}\left(t_{i+1}|t_{i}\right)$ in method 2 are generally not mathematically identical. The differences between these two formulations are discussed in the following section (Cook, 2007; Kaplan and Meier, 1958; Wang and Chang, 1999).

The exact definitions of the RTTE functions in method 2, along with their detailed mathematical derivations, are presented in Supplementary File 9.

##### 2.3.2.4 Comparison between method 2 and KM estimates

A comparison between the nonparametric KM estimates and method 2 is presented in Table 3, and the findings are largely consistent with those shown in Table 1.

**TABLE 3:** Comparison between non-parametric KM-based and Method 2 approaches for recurrent events.

Quantity	Non-parametric extension (KM-estimates)	Method 2
Level of analysis	Population-level^a^	Individual level^b^
Time scale	Calendar time	
Conditional survival	Probability of no event occurrence in the interval $(\left.t_{i},t_{i+1}\right]$	Cumulative probability that the first event does not occur in the interval $\left(t_{i},t_{i+1}\right]$
Conditional hazard	Instantaneous event occurrence rate at time $t_{i+1}$	
Conditional (density) Probability	Conditional probability of event occurrence at $t_{i+1}$ , given no event occurred in $\left(t_{i},t_{i+1}\right)$	Conditional density of a single event occurrence at time $t_{i+1}$ , given no event occurred in the interval $\left(t_{i},t_{i+1}\right)$
Event process interpretation	Event count process (multiple events possible)	Renewal-type sequential single-event process (single event after $t_{i}$ )
Dependence structure	Conditional independence given previous event	
History dependence	Dependence through survival function	

Notation: $t_{i}$ denotes the previous event time, $t_{i+1}$ denotes the current event time.

^a^ Interval-based aggregation of event information across time, irrespective of subject identity, where event information is aggregated at each event time across the entire population.

^b^ Sequential accumulation and representation of event information over time on a per-subject basis, through event-time-based modeling with time-ordered updating for each individual subject.

Unlike method 1, method 2 exhibits a probabilistic dependence structure that is conceptually similar to the Kaplan–Meier (KM)-based approach for recurrent events. Both approaches follow a conditional independence structure in which partial dependence between event occurrences is introduced through the survival function up to the previous event time $t_{i}$ . Under this framework, the conditional survival function, conditional hazard, and conditional probability density function in method 2 are conceptually similar to the probabilistic structure used in KM-based parametric extensions for recurrent events. Therefore, method 2 can be interpreted as a parametric counterpart to the KM-based recurrent event model presented in Table 1, with the key difference that the conditional survival structure is modeled using a parametric hazard function rather than a nonparametric product-limit estimator (Cook, 2007; Kaplan and Meier, 1958; Wang and Chang, 1999; Andersen et al., 1993; Kalbfleisch and Prentice, 2002).

The difference in the probabilistic dependence structure arises from how the event process after a previous event is interpreted. In nonparametric KM-based approaches, the conditional probability structure is defined for the entire process in which multiple events may occur after a previous event (Cook, 2007; Andersen et al., 1993). In contrast, for each conditional likelihood unit, method 2 defines the conditional distribution only for the time to the next event following the previous event (Collett, 2015; Kleinbaum and Klein, 2012; Lawless, 2003).

In addition, similar to method 1, method 2 adopts an individual-level modeling framework. Therefore, some differences may arise compared to KM estimates, which directly estimate the probability at the population level. These differences can be interpreted as resulting from the structural differences between parametric model-based estimation and nonparametric estimation methods.

Nevertheless, despite these differences, in most cases the KM-based nonparametric estimates and the model-based simulation results obtained using method 2 (e.g., visual predictive checks) show similar overall behavior.

##### 2.3.2.5 Special case: equivalence between method 2 and KM estimates

Although the nonparametric KM estimates and method 2 are not mathematically identical, the differences between the two approaches become negligible when the hazard function varies smoothly over time. Under such conditions, the KM-based nonparametric estimates and the model-based simulation results obtained using method 2 are often nearly indistinguishable. In many clinical situations, hazard functions tend to be relatively high at early times and gradually decrease over time rather than changing abruptly. Therefore, in most practical applications, method 2 can be appropriately considered a parametric counterpart to the KM estimates.

However, when the hazard decreases rapidly over time, for example, in situations where strong dependence exists between events or early events occur in clusters, method 2 may underestimate event occurrences. As discussed previously, this arises from structural differences between method 2 and the nonparametric estimation approach.

As a result, in parametric approaches, hazard increments are effectively updated sequentially along each subject’s event history, with likelihood contributions evaluated row by row within each subject. Consequently, the incorporation of repeated events follows an individual-level, time-ordered structure. In contrast, the KM-based nonparametric approach aggregates events across all subjects within the entire time frame and reflects their occurrence order at the population level. As a result, in parametric approaches the hazard increments tend to be estimated in an averaged form at the individual level. Consequently, when events are clustered in the early observation period and the hazard changes rapidly, these differences may become more pronounced. In addition, method 2 implicitly assumes that only one event occurs after a previous event, which may lead to additional smoothing of the early hazard increments (Cook, 2007; Kaplan and Meier, 1958; Wang and Chang, 1999; Andersen et al., 1993).

This limitation may become more pronounced when steep changes in the hazard need to be described using relatively inflexible parametric models, particularly in exponential models with covariates, such as those incorporating drug concentration, exposure metrics (e.g., area under the curve [AUC]), or non-drug effects. As a result, the estimates obtained from method 2 may differ from nonparametric estimates. Detailed mathematical derivations related to this behavior are provided in Supplementary File 12 (Kleinbaum and Klein, 2012; Lawless, 2003; OpenStax, 2022; Conrad, 2010).

Therefore, method 2 can still be widely applied in clinical situations where the hazard changes gradually over time. However, when the hazard changes rapidly or when strong dependence exists between events, alternative approaches may be required. Accordingly, method 3 is proposed as a new approach that more explicitly accounts for dependence between events.

#### 2.3.3 Method 3

Method 3, proposed in this paper, is designed to more explicitly capture risk by reflecting event-to-event dependence, particularly in settings where events occur densely in the early phase. In this approach, both the time scale and the survival function are applied consistently with method 2.

Notably, the primary distinction between methods 2 and 3 lies in the definition of the conditional hazard.

##### 2.3.3.1 Conditional hazard function

In the sequential framework of method 2, although the approach is designed to sequentially compute the cumulative hazard between two consecutive events in order to preserve the risk at the previous event time within the conditional structure, the differentiation step—where the hazard is defined as the derivative of the cumulative hazard—effectively removes the component reflecting the previous event.

To address this limitation, instead of evaluating the two event times separately, we define the conditional hazard by jointly considering both time points and by characterizing the change in cumulative hazard over the interval between them. When both time points are treated simultaneously as two possible realizations of event-time random variables that share the same underlying properties, the interpretation of $H^{\prime }\left(t_{i}\;t_{i+1}\right)$ extends beyond the instantaneous rate of change of $H\left(t\right)$ at $t_{i+1}$ . Instead, the interpretation represents the overall rate of change in the cumulative hazard over the interval ( $\left.t_{i},t_{i+1}\right]$ . Accordingly, the conditional hazard is not defined as the instantaneous risk at a specific time point, but instead as the interval-averaged hazard, obtained by dividing the increment in cumulative hazard over the interval by its length.

Mathematically, this interpretation corresponds to the average rate of change of the cumulative hazard between $t_{i}$ and $t_{i+1}$ . Therefore, the conditional hazard in method 3 can be defined as follows:

$$h\left(t_{i+1}|t_{i}\right)=\frac{1}{t_{i+1}-t_{i}}{\left[\int_{t_{0}}^{t_{2}}h\left(u\right)\;du-\int_{t_{0}}^{t_{1}}h\left(u\right)\;du\right]}_{t_{1}=t_{i},t_{2}=t_{i+1}}=\frac{\int_{t_{0}}^{t_{i+1}}h\left(u\right)du-\int_{t_{0}}^{t_{i}}h\left(u\right)du}{t_{i+1}-t_{i}}$$ (8)

where $t_{1}$ and $t_{2}$ are treated as two realized values of event-time random variables associated with $h\left(t\right)$ .

Under a counting process framework, the increment in cumulative hazard over an interval $\left(t_{i},\;t_{i+1}\right]$ corresponds to the expected number of events occurring within that interval. Based on this relationship, the conditional hazard in method 3 can be interpreted as an interval-averaged event intensity. The detailed derivations are provided in Supplementary File 13 (Andersen et al., 1993; Fleming and Harrington, 2011; Aalen et al., 2008; Singer and Willett, 2003).

According to the mean value theorem, the mean rate of change of a continuous and differentiable function $H\left(t\right)$ across an interval equals the slope of the tangent line at some point within that interval (Conrad, 2010). Therefore, if $H\left(t\right)$ is concave, corresponding to a decreasing hazard function $h\left(t\right)$ , the mean rate of change will necessarily exceed the tangent slope $h\left(t_{i+1}\right)$ at the later endpoint, $t_{i+1}$ (Boyd and Vandenberghe, 2004). This provides a natural explanation for the amplification of dependent risk, as the contribution of risk at the previous event time is carried forward and reflected in the current event risk.

In method 2, the conditional hazard is defined as the instantaneous hazard at a single time point, whereas in method 3 it is defined as an interval-averaged event intensity based on the increment of cumulative hazard over the interval. In method 3, the cumulative hazard up to the previous event time is explicitly incorporated into the definition of the hazard between successive events. As a result, the accumulated risk from prior events is naturally propagated to subsequent event risk, allowing event-to-event dependence to be more effectively captured through changes in cumulative hazard.

Figure 1 depicts the geometric relationship among $H\left(t\right)$ , $h\left(t\right)$ , and the conditional hazard function defined in methods 2 and 3, as illustrated in both observed data (KM-based plot) and estimated data (continuous parametric function).

**FIGURE 1:**
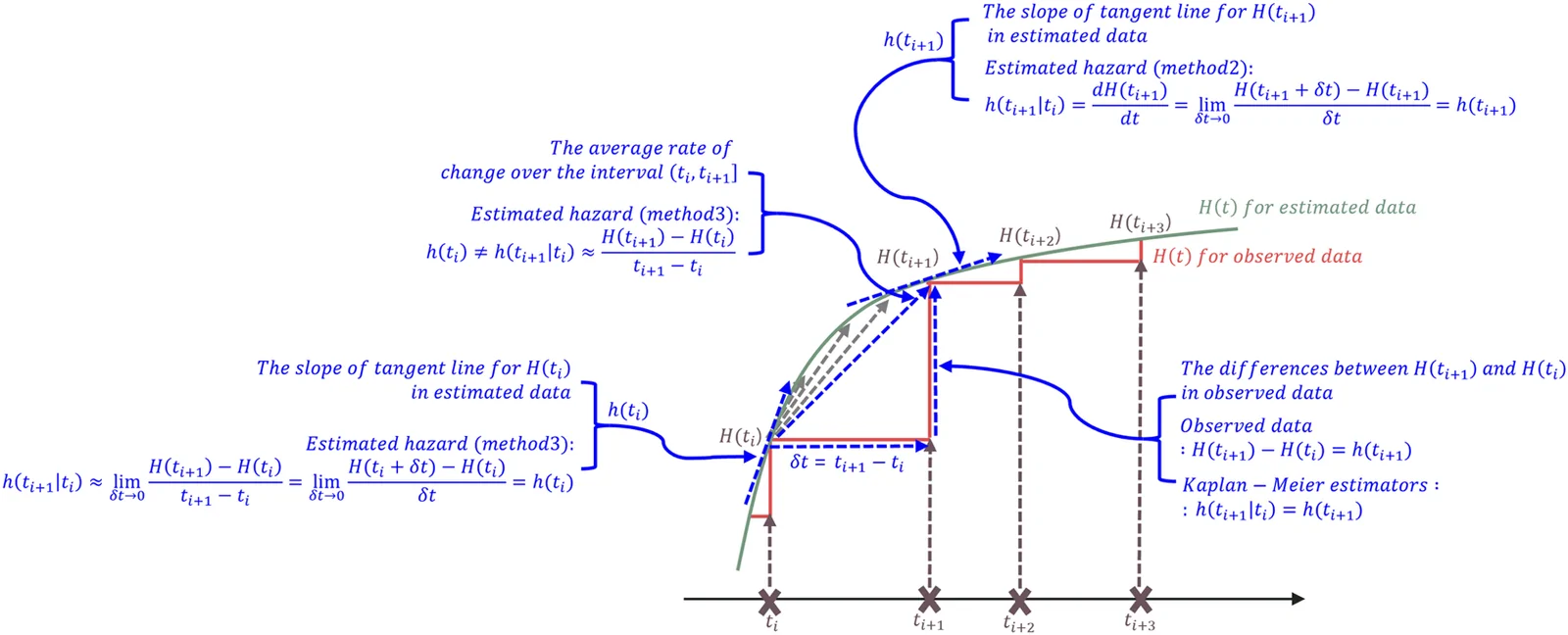
The geometric relationship between *H(t)*, *h(t)*, and the conditional hazard *h(t_i+1_|t_i_)*. Figure 1 illustrates the geometric interpretation of the conditional hazard defined in Equation 8 (Method 3), based on the average rate of change of the cumulative hazard over an event interval. The red line represents the nonparametric Kaplan -Meier estimate of the cumulative hazard function *H(t)* based on observed data, whereas the green line corresponds to the parametric estimate of *H(t)* derived from the fitted model. The observed cumulative hazard function for the discrete event-time data is typically represented as a step function. Given that the cumulative hazard *H(t)* is defined as the sum of individual hazard contributions at each observed event time, the difference *H*(*t_i+1_*) −*H*(*t*_*i*_) corresponds to the hazard at time *t_i+1_*, denoted as *h*(*t_i+1_*), which is equivalent to the conditional hazard *h(t_i+1_|t_i_)* defined in method 2 -- where the conditional hazard is defined directly as the instantaneous hazard without explicit accounting for the length of the underlying time interval -- thereby indicating that the concept of conditional hazard in method 2 is consistent with that of the KM estimators. As the discrete data are derived from the underlying continuous distribution, the conditional hazard between time points *t_i_* and *t_i+1_* can be approximated by the average rate of change in the cumulative hazard over that interval, as illustrated in the graph, consistent with the formulation adopted in method 3. Under a specific parametric assumption for the hazard distribution, this process can be extended to a continuous representation of the cumulative hazard function. As the interval *δ_t_ = t_i+1_ − t_i_* approaches zero, the average rate of change converges to the instantaneous rate of change of the cumulative hazard function. This instantaneous rate, defined as the derivative of *H(t)*, corresponds to the hazard function *h* *(t)*. Accordingly, the predicted hazard at *t_i_*, *h*(*t*_*i*_) can be interpreted as the slope of the tangent to the cumulative hazard curve at that point. Thus, in the limit of very short intervals, the average-rate-based conditional hazard approaches *h*(*t*_*i*_) rather than *h*(*t_i+1_*). *h*(*t*_*i*_) does not coincide with the conditional hazard *h*(*t_i+1_|t_i_*) = *h*(*t_i+1_*) adopted in method 2. Therefore, under a decreasing hazard and sufficiently short intervals, *h*(*t*_*i*_) is always greater than *h*(*t_i+1_*) of method 2, and method 3 leads to overestimation relative to method 2.

##### 2.3.3.2 Conditional probability density function

Unlike methods 1 and 2, method 3 defines the conditional hazard based on an interval formulation. Accordingly, $t_{i}$ and $t_{i+1}$ are treated as realizations of the same underlying continuous event-time random variable T . This formulation allows method 3 to more effectively capture the sequential dependence inherent in recurrent events.

Under this framework, the conditional probability density function $f_{k}\left(t_{i+1}|t_{i}\right)$ can be expressed as the product of the conditional survival function and the interval-averaged event intensity:

$$f_{k}\left(t_{i+1}|t_{i}\right)=S\left(t_{i+1}|t_{i}\right)\cdot h\left(t_{i+1}|t_{i}\right)=\frac{S\left(t_{i+1}\right)}{S\left(t_{i}\right)}\cdot \frac{H\left(t_{i+1}\right)-H\left(t_{i}\right)}{t_{i+1}-t_{i}}$$ (9)

In Equation 9, the conditional density is not defined as an instantaneous hazard of event occurrence at a specific time point, but rather as a quantity based on the interval event intensity defined over the interval between two event times. This formulation avoids reliance on the first-event distribution and, unlike method 2—which assumes, for each conditional likelihood unit, that a single event occurs after $t_{i}$ —allows for the possibility of multiple events occurring after $t_{i}$ .

Nevertheless, method 3 does not yield a mathematically identical form to the nonparametric KM estimator. Although conceptually consistent with the KM conditional survival structure in accounting for multiple events, method 3 defines the conditional hazard using an interval-averaged intensity, which leads to differences from KM-based estimates. These differences are further discussed in the subsequent section.

The precise definitions of the RTTE functions used in method 3 and their detailed mathematical derivations are provided in Supplementary File 9.

##### 2.3.3.3 Comparisons between KM estimates and method3

A comparison between the nonparametric KM estimates and method 3 is summarized in Table 4. Both approaches share several key features: they allow for multiple events to occur after the previous event time, adopt a conditional independence assumption, and are based on a conditional survival framework. However, the two methods differ fundamentally in how the conditional hazard is defined, which in turn leads to differences in the form of the conditional probability density function. Specifically, the KM approach defines the conditional hazard as an instantaneous event occurrence rate at a given time point, whereas method 3 defines it as an interval-averaged hazard based on the increment of cumulative hazard over the interval between successive event times (Kaplan and Meier, 1958; Wang and Chang, 1999).

**TABLE 4:** Comparison between non-parametric KM-based and Method 3 approaches for recurrent events.

Quantity	Non-parametric extension (KM-estimates)	Method 3
Level of analysis	Population-level^a^	Individual level^2)^
Time scale	Calendar time	
Conditional survival	Probability of no event occurrence in the interval $(\left.t_{i},t_{i+1}\right]$	Cumulative probability that the first event does not occur in the interval $\left(t_{i},t_{i+1}\right]$
Conditional hazard	Instantaneous event occurrence rate at time $t_{i+1}$	Interval-averaged event intensity based on cumulative hazard increment in the interval $\left(t_{i},t_{i+1}\right]$
Conditional (density) Probability	Conditional probability of event occurrence at $t_{i+1}$ , given no event occurred in $\left(t_{i},t_{i+1}\right)$	Conditional probability density of event occurrence at $t_{i+1}$ defined using interval-averaged hazard, given no event occurred in $\left(t_{i},t_{i+1}\right)$
Event process interpretation	Event count process (multiple events possible)	Event time process with interval event intensity (multiple events possible)
Dependence structure	Conditional independence given previous event	
History dependence	Dependence through survival function	Dependence through both survival function and cumulative hazard in the interval $\left(t_{i},t_{i+1}\right]$

Notation: $t_{i}$ denotes the previous event time, $t_{i+1}$ denotes the current event time.

^a^ Interval-based aggregation of event information across time, irrespective of subject identity, where event information is aggregated at each event time across the entire population.

^b^ Sequential accumulation and representation of event information over time on a per-subject basis, through event-time-based modeling with time-ordered updating for each individual subject.

As a result, discrepancies may arise between KM-based estimates and simulation results obtained using method 3. According to the mean value theorem, when the hazard function is decreasing, the interval-averaged hazard exceeds the instantaneous hazard at the end of the interval (Conrad, 2010). Consequently, method 3 tends to overestimate the risk relative to KM estimates in the early period. Conversely, when the hazard function is increasing, the interval-averaged hazard becomes smaller than the instantaneous hazard at the end of the interval, leading method 3 to underestimate the risk relative to KM estimates in the early period.

Furthermore, similar to methods 1 and 2, method 3 is a parametric, model-based approach that updates risk sequentially at the individual level following each event, whereas KM estimates are derived nonparametrically at the population level. This difference in the level of inference introduces structural discrepancies between the two approaches. Nevertheless, in certain scenarios, the modeling structure of method 3 can mitigate these differences, as discussed in the following section.

##### 2.3.3.4 Special cases: equivalence between method 3 and KM estimates

As hazard functions most commonly exhibit a decreasing trend, we focus on this setting and further categorize it into two scenarios: one in which the hazard changes smoothly and another in which it changes abruptly (Amorim and Cai, 2015; Rauch et al., 2018).

This study provides mathematical derivations for quantifying the differences between the probability density function $\hat{f\left(t_{i+1}|t_{i}\right)}$ of the KM method and $f_{k}\left(t_{i+1}|t_{i}\right)$ in methods 2 and 3 under conditions of smoothly varying versus rapidly changing hazard functions. Full details are presented in Supplementary File 14 (Kleinbaum and Klein, 2012; Lawless, 2003; OpenStax, 2022; Conrad, 2010; Miller, 2016). Notably, when the hazard varies smoothly, the discrepancy between $f_{k}\left(t_{i+1}|t_{i}\right)$ in method 2 and $\hat{f\left(t_{i+1}|t_{i}\right)}$ in the KM method is smaller. In contrast, when the hazard exhibits steep or abrupt changes, the discrepancy between $f_{k}\left(t_{i+1}|t_{i}\right)$ in method 3 and $\hat{f\left(t_{i+1}|t_{i}\right)}$ in the KM method becomes smaller.

Accordingly, method 2 may be less appropriate in settings where events occur frequently, when the hazard changes rapidly following initial exposure, or when prior events exert a strong influence on subsequent risk (e.g., drug-induced adverse events), leading to pronounced event-to-event dependence. In such cases, the discrepancy between the KM-based results-reflecting cumulative population-level risk and the parametric individual-level estimates $f_{k}\left(t_{i+1}|t_{i}\right)$ obtained from method 2 may become more evident, partly due to the averaging inherent in individual-level updating. Conversely, method 3 may help reduce these discrepancies by explicitly incorporating a cumulative prior-event risk into the conditional hazard structure, thereby amplifying conditional cumulative risk at the individual level while aligning more closely with the KM-based cumulative risk at the population level. In these settings, the divergence between KM curves and method 3 often remains smaller than that observed with method 2.

These discrepancies may become more pronounced in scenarios where events are densely concentrated in the early period and the hazard changes rapidly, particularly when inflexible parametric models are used (e.g., exponential models incorporating covariates such as drug concentration, cumulative drug exposure (area under the curve, AUC), or non-drug effects). Given the diversity of clinical scenarios, method 3 does not universally provide a better fit than method 2. However, when hazards vary sharply and events are heavily concentrated in the early phase, especially in exponential models with multiple covariates, method 2 may tend to underestimate the hazard relative to KM estimates. In such cases, method 3 may serve as a useful alternative.

Figure 2 provides an intuitive illustration of the correspondence between the mathematical formulations of each method and their implementation in NONMEM® (Version 7.5). The NONMEM® control stream code corresponding to method 3, derived from the mathematical formulations presented above, is provided in Supplementary File 15. The characteristics of each method, their comparison with KM estimates, and the corresponding scenarios in which each method is most appropriate are summarized in Table 5.

**FIGURE 2:**
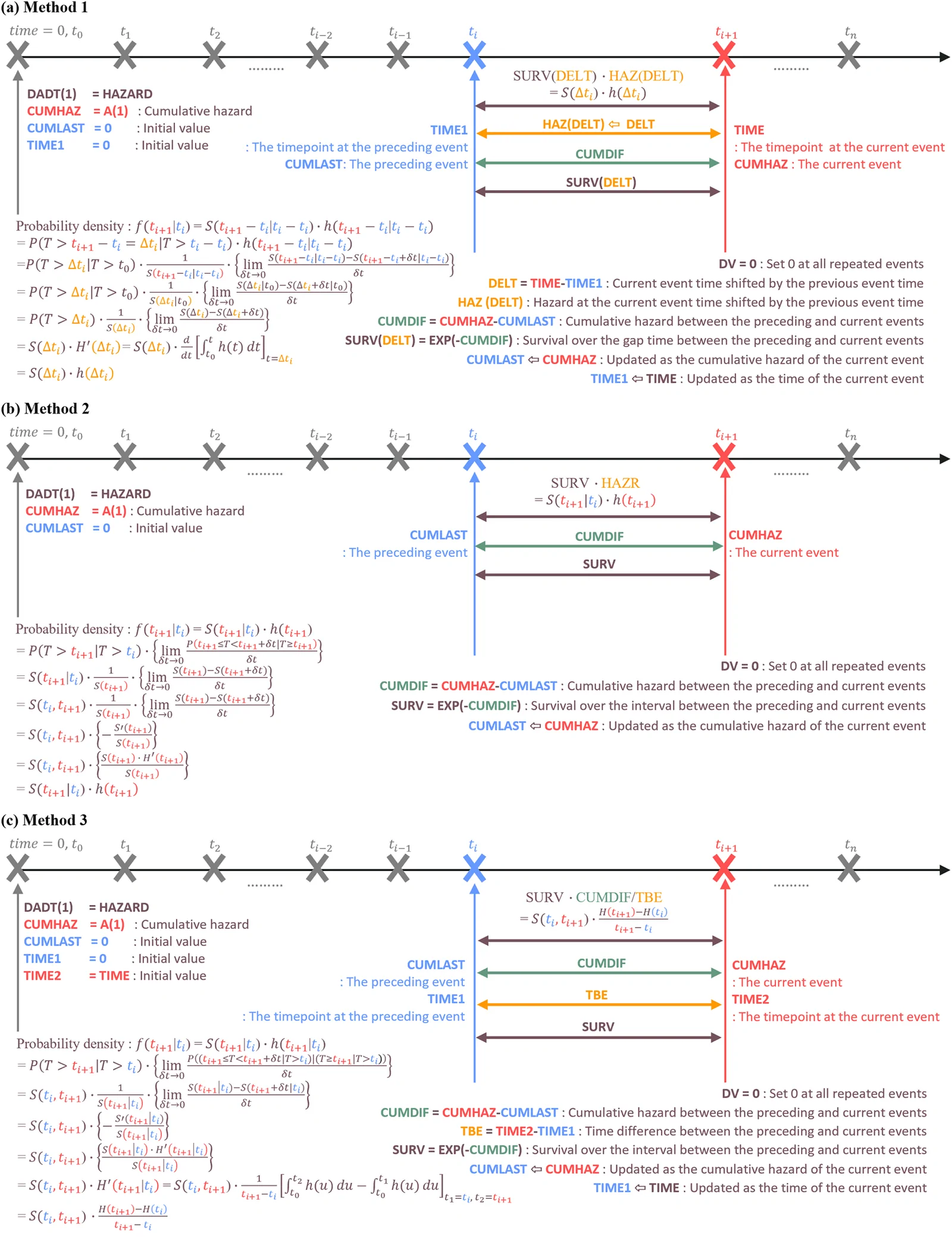
Probability density function and NONMEM® code for the three methods in the repeated exact time events model. **(a–c)** In the manuscript body, each time point associated with the *k*th subject is denoted by a subscript that includes *k.* However, for the sake of notational simplicity in Figure 2, the subscript *k* has been omitted, and only an index indicating the sequential order of time points has been retained.

**TABLE 5:** Comparative overview of non-parametric and parametric methods for repeated exact time event analysis.

Contents		Non-parametric method	Parametric method		
		KM-plot VPC	Method 1	Method 2	Method 3
Level of analysis		Population-level estimation^a^	Individual-level estimation^b^		
Time scale		Calendar time	Gap time (Time-shift)	Calendar time	
Survival function	Previous event	Treated as realized constant Condition on previous event time	Reset as $t_{0}$ at previous time	Treated as realized constant Condition on previous event time	
	Conditionality	O	X	O	
	Definition	Conditional survival function (conditional cumulative probability)	Survival function (cumulative probability)	Conditional survival function (conditional cumulative probability)	
	Equation	$\hat{S\left(t\right)}=\prod_{t_{i}\leq t}\left(1-\Delta H_{i}\right)$	$S\left(\Delta t_{i}\right)$	$S\left(t_{i+1}|t_{i}\right)$	
Cumulative hazard function	Previous event	Treated as realized constant Condition on previous event time	Reset as $t_{0}$ at previous time	Treated as realized constant Condition on previous event time	Previous and current event times treated as joint random variables
	Conditionality	O	X	O	
	Definition	Conditional cumulative hazard	Cumulative hazard	Conditional cumulative hazard	
	Equation	$\Delta H_{i}$	$H\left(\Delta t_{i}\right)$	*H*(*t_i+1_|t_i_*) = *H*(*t_i_, t_i+1_*) = *H*(*t_i+1_*)* − H*(*t_i_*)	
Hazard function	Previous event	Treated as realized constant Condition on previous event time	Reset as $t_{0}$ at previous time	Treated as realized constant Condition on previous event time	Previous and current event times treated as joint random variables
	Conditionality	X			O
	Definition	Unconditional hazard (instantaneous event rate)			Conditional hazard (conditional event rate)
	Equation	$h\left(t\right)=\frac{\Delta H_{i+1}}{\Delta t_{i+1}},t\in \left[t_{i+1},t_{i+2}\right)$	$h\left(\Delta t_{i}\right)$	$h\left(t_{i+1}\right)$	$\frac{H\left(t_{i+1}\right)-H\left(t_{i}\right)}{t_{i+1}-t_{i}}$
Probability density function	Number of random variable	1 ( $\left.t_{i+1}\right)$	1 $\left(\Delta t_{i}\right)$	1 ( $\left.t_{i+1}\right)$	2 ( $\left.t_{i+1},t_{i}\right)$
	Differentiable of F	Possible			Not possible
	Definition	Conditional probability mass function	Probability density function	Conditional probability density function	
	Equation $f_{k}\left(t_{i+1}|t_{i}\right)$	$\hat{f\left(t_{i+1}|t_{i}\right)}=\hat{P}\left(T=t_{i+1}|T>t_{i}\right)$	$P\left(T=\Delta t_{i}\right)=f\left(\Delta t_{i}\right)$	$P\left(T=t_{i+1}|T>t_{i}\right)=f\left(t_{i+1}|t_{i}\right)$	$P\left(T>t_{i+1}|T>t_{i}\right)\cdot \frac{\left\{-log\;P\left(T>t_{i+1}\left|T\right.>t_{i}\right)\right\}}{\Delta t_{i}}$
	Event process interpretation	Event count process (multiple events possible)	Renewal process (single event after $t_{i}$ , reset as $t_{0}$ )	Renewal-type sequential single-event process (single event after $t_{i}$ )	Event time process with interval event intensity (multiple events possible)
​	Dependence structure	Conditional independence given previous event	Renewal independence (independent gap times)	Conditional independence given previous event	
	History dependence	Dependence through survival function	No dependence on previous event history	Dependence through survival function	Dependence through both survival function and cumulative hazard in the interval $\left(t_{i},t_{i+1}\right]$

Abbreviations: KM, Kaplan-Meier; VPC, visual predictive check; *ΔH_i_ = e_i+1_/n_i+1_ = e_i+1_/(n_i_ − c_i_)*, where $e_{i+1}$ is the number of events at $t_{i+1}$ , $n_{i}$ ​ is the number of subjects at risk just prior to $t_{i}$ , $n_{i+1}$ ​ is the number of subjects at risk just prior to $t_{i+1}$ and $c_{i}$ ​ is the number of censored subjects within the interval $(\left.t_{i},t_{i+1}\right]$ ; $\Delta t_{i}=t_{i+1}-t_{i}$ .

^a^ Interval-based aggregation of event information across time, irrespective of subject identity, where event information is aggregated at each event time across the entire population.

^b^ Sequential accumulation and representation of event information over time on a per-subject basis, through event-time-based modeling with time-ordered updating for each individual subject.

### 2.4 Hazard change patterns across base models without covariates

In the exponential model, the hazard rate is constant, so all three methods (methods 1, 2, and 3) yield identical conditional hazards. In contrast, the Weibull and log-logistic models exhibit curvatures of $H\left(t\right)$ that vary with their parameters (Kleinbaum and Klein, 2012; Boyd and Vandenberghe, 2004). Based on the mathematical formulations of the three models presented in Supplementary File 3 (Collett, 2015; Lim, 2021), the increasing and decreasing patterns of the hazard function can be summarized in Table 6, depending on the ranges of λ and γ . The relevant derivations are provided in Supplementary File 16 (Kleinbaum and Klein, 2012; Boyd and Vandenberghe, 2004).

**TABLE 6:** Summary of hazard and cumulative hazard patterns across parametric models.

Model	Condition		$h\left(t\right)$ pattern	$H^{\prime \prime }\left(t\right)$	$H\left(t\right)$ pattern
Exponential	γ=1		Constant	$H^{\prime \prime }\left(t\right)=0$	Straight line
Weibull	γ<1		Decreasing	$H^{\prime \prime }\left(t\right)<0$	Concave
	γ>1		Increasing	$H^{\prime \prime }\left(t\right)>0$	Convex
Log-logistic	γ≤1		Decreasing	$H^{\prime \prime }\left(t\right)<0$	Concave
	γ>1	$t<\lambda {\left(\gamma -1\right)}^{1/\gamma }$	Increasing	$H^{\prime \prime }\left(t\right)>0$	Convex
		$t>\lambda {\left(\gamma -1\right)}^{1/\gamma }$	Decreasing	$H^{\prime \prime }\left(t\right)<0$	Concave

Notation: In the Weibull model with γ<1 , or in the log-logistic model with γ≤1 , as well as in the log-logistic model with γ>1 and $t>\lambda {\left(\gamma -1\right)}^{1/\gamma }$ , the cumulative hazard function $H\left(t\right)$ is concave, indicating a decreasing hazard (Kleinbaum and Klein, 2012; Boyd and Vandenberghe, 2004). This yields positive residuals, and method 3 overpredicts relative to method 2. Conversely, in the Weibull model with γ>1 , as well as in the log-logistic model with γ>1 and $t<\lambda {\left(\gamma -1\right)}^{1/\gamma }$ , the cumulative hazard function $H\left(t\right)$ is convex, reflecting an increasing hazard. Under these conditions, residuals are negative, and method 3 underpredicts relative to method 2 (Collett, 2015; Boyd and Vandenberghe, 2004).

In most clinical scenarios, the hazard tends to decrease over time unless aging effects are explicitly modeled (Audenaert and Aujla, 2011), and the shape parameter (γ) in Weibull or log-logistic models is typically less than 1 (Collett, 2015). As a result, during the early phase immediately following drug administration—when event intervals are short—the cumulative hazard function is generally concave. In this context, under a base model without covariates, method 3 tends to overestimate the hazard relative to method 2.

### 2.5 Comparisons of VPC between method 2 and method 3 in base models without covariates

To examine how methods 2 and 3 differ across time intervals under a base model without covariates (i.e., the exponential, Weibull, and log-logistic models defined in Supplementary File 3), this study analyzes the mathematical differences in the definition of the conditional hazard for each method.

The difference between methods 2 and 3 can be understood through a Taylor series expansion of the cumulative hazard function $H\left(t\right)$ at the previous event time $t_{i}$ . Under this expansion, the conditional hazard in method 3 can be expressed as the unconditional hazard in method 2 plus a residual term. When the inter-event interval Δt is small, this residual is dominated by the leading term and its sign is determined by the curvature of $H\left(t\right)$ , i.e., the second derivative $H^{\prime \prime }\left(t\right)$ . Specifically, when $H\left(t\right)$ is concave (decreasing hazard), the residual becomes positive, whereas when $H\left(t\right)$ is convex (increasing hazard), the residual becomes negative. The detailed mathematical derivation of this relationship, based on a Taylor series expansion of the cumulative hazard function, is provided in Supplementary File 17 (OpenStax, 2022; Miller, 2016).

Consequently, during the early observation period with short event intervals, a decreasing hazard produces positive residuals, leading method 3 to overpredict relative to method 2. Conversely, when the hazard is increasing, the residuals are negative, causing method 3 to underpredict. In the mid-to-late stages of observation, where event intervals are longer and hazard changes are negligible, the residuals converge toward zero, and the difference between methods 2 and 3 becomes minimal.

When interpreted relative to KM estimates, under a base model without covariates (with a decreasing hazard), scenarios characterized by early clustering of events and rapidly changing hazards may lead to a pronounced difference between methods 2 and 3, with method 3 tending to yield higher estimates than method 2, potentially resulting in different levels of agreement with the KM estimates. As events become less frequent and inter-event intervals increase over time, the discrepancies between KM estimates and both methods 2 and 3 tend to converge, resulting in similar levels of agreement.

A detailed comparison between methods 2 and 3 across different observation periods is provided in Table 7.

**TABLE 7:** Differences between method 2 and method 3 over time.

Observation period	Hazard pattern	Inter-event interval	Residual sign	Method 3 vs. method 2
Early phase	Decreasing hazard	Short	Positive	Method 3 overestimates relative to method 2
	Increasing hazard	Short	Negative	Method 3 underestimates relative to method 2
Mid-to-late phase	Approximately constant hazard	Longer	Converges to zero	Method 2 ≈ method 3
Late phase		Much longer	Approaches zero	Minimal difference

Notation: This table summarizes representative scenarios assuming short inter-event intervals during the early observation period. When the distribution of inter-event intervals differs over time, the interpretation of the table may vary accordingly. Therefore, the patterns presented here should not be regarded as universally applicable.

Furthermore, under a base model without covariates, it is important to account for the differences between methods as outlined above and to appropriately refine the model structure for each method to ensure adequate agreement with the KM estimates used as a reference.

### 2.6 Practical guidelines for method selection

Based on the preceding analysis of the mathematical properties of each method and their behavior under different hazard patterns, we summarize practical guidelines to inform method selection in parametric analyses of recurrent event data. The key findings are summarized in Table 8.

**TABLE 8:** Practical guidelines for method selection based on hazard patterns.

Hazard pattern	Event pattern	Type of fitted models	Recommended method	Notes
Constant hazard	Approximately homogeneous event occurrence	Not model-dependent	Method 1, 2, 3	All methods agree well with KM estimates
Decreasing hazard				
Smoothly changing hazard	Sparse events or weak event dependency	Most parametric models	Method 2	Method 2 shows good agreement with KM estimates Method 3 tends to overestimate relative to KM estimates
Rapidly changing hazard	Dense early events or strong event dependency	Base models Without covariates^a^	Method 2	Method 2 generally fits KM estimates well overall or tends to underestimate relative to KM estimates during the early phase
			Method 3	Method 3 tends to overestimate relative to KM estimates during the early phase but shows good agreement with KM estimates in later periods.
		Exponential model + Covariates^b^	Method 3	Method 2 tends to underestimate relative to KM estimates Method 3 shows better agreement with KM estimates

Notation: This table is intended to provide general guidance for selecting appropriate methods in parametric analyses of recurrent events. However, the observed performance and estimation tendencies may vary depending on the model specification (e.g., base model or exponential model with covariates), event occurrence patterns, and the observation period. Therefore, the results presented here should be interpreted as indicative trends rather than strict or universally applicable rules.

^a^ Typically includes parametric models with inherent flexibility (e.g., Weibull, log-logistic), excluding simple exponential models.

^b^ Represents parametric models with limited flexibility (relatively inflexible parametric models) in capturing steep hazard changes, particularly when multiple covariates are included.

Overall, the guidelines highlight that method selection is primarily driven by hazard dynamics and event clustering, with method 2 generally preferred for smoothly varying hazards and method 3 providing advantages in scenarios with rapid hazard changes or strong event dependence.

Additionally, increasing hazard patterns are relatively uncommon in typical clinical data; therefore, this scenario was not specifically investigated in the present study.

### 2.7 Comparison of likelihood values among methods 1, 2, 3

The likelihood values derived from the three methods differ and can be distinguished according to how each defines the conditional hazard. Detailed equations and explanations of these likelihood formulations are provided in Supplementary File 18 (Collett, 2015; Cook, 2007; Rudin, 1976; Pawitan, 2001).

The likelihood values obtained from methods 1, 3, and 2 were consistently smaller in that order across the examples described in the following sections. A detailed mathematical exposition of this comparison is presented in Supplementary File 19 (OpenStax, 2022; Conrad, 2010; Boyd and Vandenberghe, 2004; Miller, 2016; LibreTexts, 2023; Rosen, 2012; Knopp, 1951).

Together, these comparisons demonstrate that under a decreasing hazard (i.e., a concave cumulative hazard function), with events densely concentrated in the early period, the likelihood values follow the order: method 1 > method 3 > method 2, while the objective function values follow the reverse order: method 1 < method 3 < method 2 (Collett, 2015; Cook, 2007; Rudin, 1976; Pawitan, 2001).

Therefore, in cases of decreasing or constant hazard, the observed differences in objective function values primarily reflect variations in mathematical computation methods rather than differences in model performance. Accordingly, we did not evaluate the comparative explanatory power of the three methods based on the magnitude of the objective function, because the relative magnitude of log-likelihood or objective function values is meaningful only when the compared models share an identical likelihood formulation and are in a nested relationship (Collett, 2015; Venables and Ripley, 2002).

### 2.8 Implementation of model fitting, simulation, and evaluation in NONMEM®

A comparative summary of the NONMEM® control stream implementations for each method is provided in Supplementary File 20. The individual control stream codes for methods 1, 2, and 3 are provided in Supplementary Files 7, 8, 15, respectively.

#### 2.8.1 Fitting datasets

Two datasets were analyzed in the repeated exact-time event analyses. The first was “Recurrence times of *tumors in rats on a retinoid treatment or control”* from Table 13.2 (p. 439) of *Modeling Survival Data in Medical Research* [third Edition] (Collett, 2015). The second comprised exact time AE data from a clinical trial of an obesity treatment. To preserve confidentiality and prevent the disclosure of the original clinical records, the AE event times were processed using jittering. The tumor recurrence dataset was well-balanced, without pronounced clustering in the early follow-up, and was sparsely distributed across the entire observation period. In contrast, the obesity treatment AE dataset displayed a sharp increase in event risk immediately following drug administration. Detailed descriptions and formatting guidelines for the NONMEM® datasets are provided in Tables 9 and Supplementary File 21.

**TABLE 9:** Summary of two examples of the repeated exact-time events.

Type	Repeated exact-time event	
Data	Female rat data (Collett, 2015)	Curated and jittered from real adverse events data
Total subjects	48 (23 treated, 25 controls)	265
Total events	211	468
Follow-up period	Approximately 122 days 18 weeks	Approximately 23 weeks 161 days, 3,864 h
Treatment	Retinoid prophylaxis	Obesity drug
Dosing time	First dosing at time 0	First dosing at time 0

Supplementary clarification concerning the female rat dataset (Collett, 2015): To investigate the effect of retinoid treatment on tumor recurrence, an animal study was conducted in which female rats were randomly assigned to either a treatment or a control group. Following exposure to a carcinogen, all rats received retinyl acetate, a vitamin A derivative with potential chemopreventive properties. Sixty days post-exposure, 48 tumor-free rats were re-randomized: one group continued receiving retinoid treatment, while the other received no further intervention. Tumor assessments were conducted twice weekly until 122 days post-randomization, when observations were terminated. Rats with no tumors on day 122 were considered censored. Female rat data present the time from randomization to first tumor development for 23 treated and 25 control rats. Since assessments were not performed daily, multiple tumors could be detected at one visit. To resolve tied event times, slight time shifts were introduced in increments of 0.1 days (e.g., 42.0, 42.1, 42.2 days) to distinguish multiple tumor occurrences observed on the same day.; Supplementary clarification on the curated and jittered real adverse event dataset: This dataset comprises a curated and jittered subset extracted from adverse event data collected following the administration of an obesity drug. For each patient, multiple episodes of a specific adverse event were documented, with numerous occurrences recorded. Events with precisely recorded occurrence times were curated and temporally jittered, with event times systematically adjusted for analytical purposes. As in the female rat dataset, when multiple events occurred at the same time point, they were separated by applying a time shift in increments of 0.1 h; these were used to construct the dataset of repeated exact-time events.

#### 2.8.2 Model fitting

In this study, methods 1, 2, and 3 were independently applied to two datasets presented in Table 9. All model fitting was performed using NONMEM® (version 7.5) with the conditional Laplace method (METHOD = COND LAPLACE) (Bauer et al., 2022). For the rat recurrence dataset, three parametric distributions—exponential, Weibull, and log-logistic—were implemented for each method, resulting in a total of nine fitted models. The mathematical formulations and parameter definitions for each model are provided in Supplementary File 3 (Collett, 2015; Lim, 2021). For the jittered AE dataset, two analysis strategies were considered. First, base models without covariates (i.e., the exponential, Weibull, and log-logistic models defined in Supplementary File 3) were fitted across all methods. Second, extended models incorporating covariates (e.g., drug concentration and AUC) were developed based on the exponential model and applied to each method. Nonparametric analysis was conducted using Kaplan–Meier (KM) estimation implemented in R (version 4.4.1). Within each method, model selection was based on the minimization of the objective function value and stable convergence, with additional consideration of visual predictive check (VPC) results. Direct comparison of objective function values across methods was not performed, as the models were not nested (Collett, 2015; Venables and Ripley, 2002).

#### 2.8.3 Simulation dataset

Simulation datasets were constructed to preserve the structure of the original data, including the number of individuals, observation periods, and covariate distributions. Evaluation time grids were defined, with rat data collected at 2-day intervals up to 122 days, and AE data recorded at 15-h intervals.

#### 2.8.4 Simulation implementation in NONMEM®

Simulations were performed using a Monte Carlo approach based on the estimated hazard structures. For each model and method, 100 replicated datasets (n = 265 per replicate) were generated with fixed parameter estimates. Event occurrences were generated sequentially over time for each individual, reflecting the temporal structure following previous events and thereby preserving event-history structure specified by each method. Detailed mathematical formulations and the implementation algorithm used for event generation are provided in Supplementary File 22.

#### 2.8.5 Simulation-based evaluation

For each simulated dataset, survival functions and cumulative event probabilities were computed and compared with Kaplan–Meier estimates derived from the observed data. Model performance was primarily evaluated using visual predictive checks (VPC), in which simulated percentiles (5th, 50th, and 95th) were compared against observed KM curves. For each method, the model that demonstrated the closest agreement with the KM-based patterns was selected as the best-fitting model (Bonate, 2011; Kaplan and Meier, 1958).

## 3 Results

### 3.1 Tumor recurrence times in rats

The modeling results for the rat dataset (repeated, exact TTE data on breast tumor recurrence in female rats) were evaluated using three methods implemented in NONMEM® (Version 7.5). Parameter estimates and objective function values for all three models across the three methods are presented in Table 10. In every case, the estimation was successfully converged, and the associated standard error (SE) was sufficiently small. In all cases, the 95% confidence intervals excluded zero, and the absolute values of the parameter estimates exceeded 1.96 × SE, confirming the statistical significance of all parameters based on Wald tests.

**TABLE 10:** Summary of the results in NONMEM^®^ for repeated exact time event data.

Distribution	Estimator		NONMEM®		
			Method 1	Method 2	Method 3
Tumor recurrence in female rat data (Collett, 2015)					
Exponential	Objective function value		1824.467	1824.467	1824.467
	Lamda(λ), (day^-1^)	Estimate	3.60E-02	3.60E-02	3.60E-02
		S.E	4.15E-03	4.15E-03	4.15E-03
Weibull	Objective function value		1790.730	1823.978	1805.621
	Lamda(λ), (day^-1^)	Estimate	3.92E-02	3.36E-02	6.34E-02
		S.E	5.13E-03	4.48E-03	1.74E-03
	Gamma(γ), (unitless)	Estimate	7.35E-01	1.05E+00	7.24E-01
		S.E	5.09E-02	7.34E-02	4.99E-02
Log-logistic	Objective function value		1814.379	1843.831	1829.384
	Lamda(λ), (day)	Estimate	1.45E+01	2.20E+01	1.25E+01
		S.E	2.07E+00	3.01E+00	2.22E+00
	Gamma(γ), (unitless)	Estimate	9.84E-01	2.30E+00	1.77E+00
		S.E	7.96E-02	2.50E-01	2.35E-01
Jittered adverse events data (Base model)					
Exponential	Objective function value		8001.72	8001.721	8001.721
	Lamda(λ), (h^-1^)	Estimate	5.27E-04	5.27E-04	5.27E-04
		S.E	7.14E-05	7.14E-05	7.14E-05
Weibull	Objective function value		6602.304	7368.769	6948.377
	Lamda(λ), (h^-1^)	Estimate	1.00E-04	1.38E-03	2.93E-03
		S.E	-	3.90E-04	1.02E-03
	Gamma(γ), (unitless)	Estimate	2.51E-01	3.80E-01	2.54E-01
		S.E	-	3.20E-02	3.19E-02
Log-logistic	Objective function value		6474.718	7355.554	6962.935
	Lamda(λ), (h)	Estimate	4.17E+02	2.14E+02	7.33E+01
		S.E	1.34E+02	5.47E+01	1.82E+01
	Gamma(γ), (unitless)	Estimate	4.02E-01	5.77E-01	4.05E-01
		S.E	2.64E-02	5.92E-02	1.76E-02
Jittered adverse events data (Exponential model with covariates)					
Exponential + covariates^a^	Objective function value		7274.631	7429.481	7295.464

Abbreviations: λ, model parameter; γ, shape parameter. SE, standard error.

^a^ The covariates incorporated into the exponential model were derived directly from the original clinical trial dataset in which the adverse events (AEs) were collected, including drug concentration, area under the curve (AUC), and non-drug effects, etc. A comprehensive analysis of the AE, data from the original clinical trial is beyond the scope of the present study and will be reported elsewhere; therefore, detailed parameter estimates are not presented here.

Figure 3 presents the visual predictive checks (VPCs) of the cumulative probability of recurrent events in the rat dataset. Given the absence of an initial steep slope and the sparse distribution of follow-up data over the entire observation period in the rat dataset, method 2 may be regarded as theoretically the most comparable to the repeated KM plot among the three approaches. In Figure 3, method 2 consistently reproduced the closest agreement with the repeated KM curves across all three base models, resulting in the best overall fit.

**FIGURE 3:**
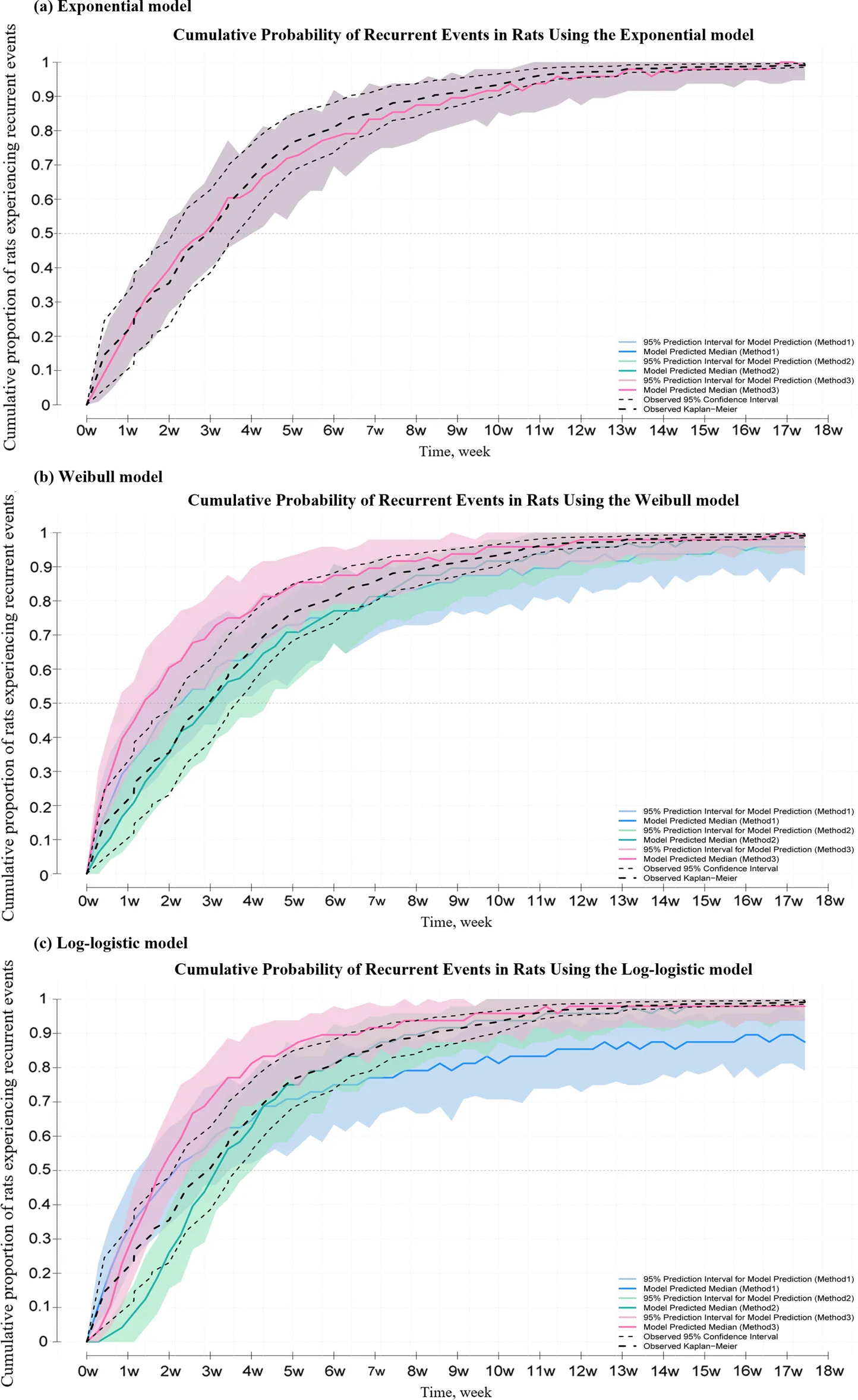
Visual predictive checks (VPCs) of the repeated exact time events for the three base models without covariates (rat data) **(a)** Exponential model **(b)** Weibull model **(c)** Log-logistic model.

Because the exponential model assumes a constant hazard, the instantaneous hazard derived via time-shifting in method 1, the instantaneous hazard at real-time in method 2, and the average cumulative hazard change rate in method 3 all yield equivalent estimates. Accordingly, the exponential model produced identical parameter estimates and objective function values across all three methods.

The Weibull model consistently achieved the lowest objective function values across methods. Although the Weibull model under method 2 produced a slightly lower objective function value than the exponential model, the reduction did not exceed 3.84 despite the addition of one parameter. For this reason, the exponential model can be conclusively identified as the best-fitting model. Additionally, the comparison with the nonparametric analysis (repeated KM plot) and VPCs demonstrated that the exponential model provided the best fit.

With respect to the Weibull model, methods 1 and 3 tended to overpredict the cumulative probability in the early phase compared with the nonparametric estimates, whereas method 2 more closely matched the KM plot. However, whereas method 3 maintained this overprediction throughout the entire 18-week period, method 1 transitioned to an underprediction beginning around weeks 4–5.

Meanwhile, both methods 2 and 3 exhibited slight underprediction during the early stages in the log-logistic model. After approximately 1 week, method 3 began to overpredict, while method 2 continued to show underprediction until around 3.5 weeks, after which method 2 started to align closely with the nonparametric analysis over time. Comparatively, method 1 showed an initial overprediction, followed by underprediction after approximately 6 weeks.

Supplementary File 23 presents the probability of remaining free of recurrent events in the rat dataset. A detailed methodological interpretation of Figure 3 is provided in Supplementary File 24 (Collett, 2015; Cook, 2007; Kleinbaum and Klein, 2012; Aalen et al., 2008; Rudin, 1976; Boyd and Vandenberghe, 2004; Leroy et al., 2014; Singer and Willett, 2003; epiR package documentation, 2026; Miller, 2016).

### 3.2 Jittered adverse events in patients

Our analysis of jittered AE data proceeded in two steps: first, we investigated discrepancies between the KM plot and the simulation plot derived solely from the base model without covariates (i.e., the exponential, Weibull, and log-logistic models as defined in Supplementary File 3), this study subsequently, we compared the KM estimates with those from an exponential model incorporating relevant covariates.

#### 3.2.1 Base models: fitting results

The parameter estimates of the base models for each method are presented in Table 10, along with the corresponding objective function values. In all approaches, the parameter estimation converged successfully. Additionally, except for the Weibull model in method 1, where the SE was not reported, all parameter estimates had reasonably small SEs; in all cases, the 95% confidence intervals excluded zero and the absolute values of the parameter estimates exceeded 1.96 × SE, confirming the statistical significance of all parameters based on Wald tests. Thus, the Wald test confirmed the statistical significance of the parameters. Similar to the rat dataset, the lowest objective function value was observed with method 1, followed by methods 3 and 2, respectively.

Figure 4 presents simulation results based exclusively on the three base models. The KM curve, obtained via nonparametric estimation, demonstrates a steep rise in the early event rate, followed by a slight decline in slope beginning around day 3. Subsequently, approximately 1.5 weeks of post-treatment, a pronounced secondary increase in the event rate is observed, after which the rate gradually tapers off.

**FIGURE 4:**
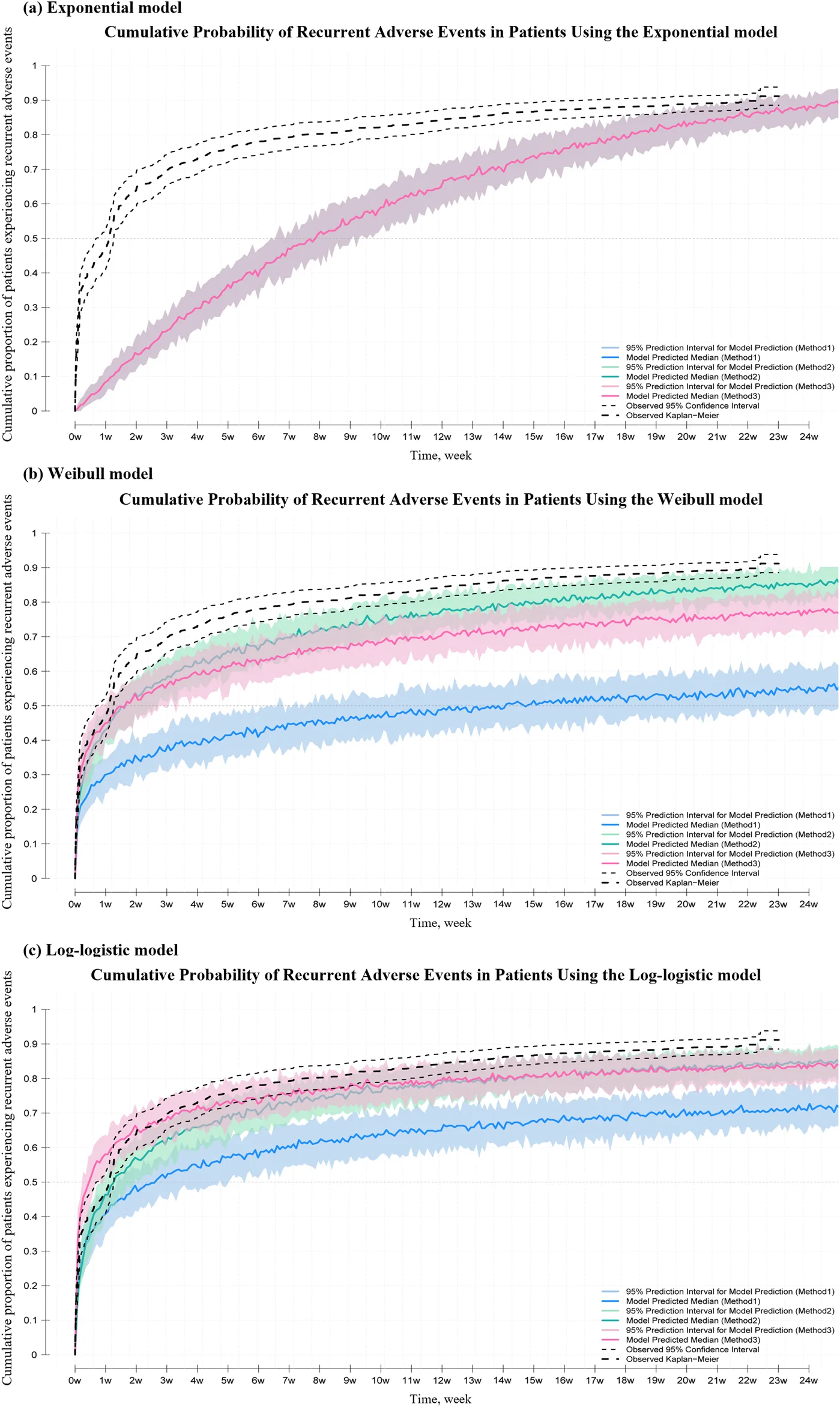
Visual predictive checks (VPCs) of the repeated exact time events for the three base models without covariates (jittered adverse events data) **(a)** Exponential model **(b)** Weibull model **(c)** Log-logistic model.

As shown in Figure 4a, the exponential model produced identical outputs across the three methods described in this paper (Collett, 2015). The simulation generated from the exponential model consistently underpredicted the KM curve in the VPC plots throughout the follow-up period, failing to capture the sharp fluctuations in hazard and instead depicting a more gradual accumulation of events. This finding underscores that a constant hazard model cannot adequately represent the AEs.

As shown in Figure 4b, the Weibull model demonstrated the best fit with method 3 up to approximately 1.5 weeks. Beyond this time point, no VPCs showed complete agreement with the nonparametric Kaplan–Meier (KM) estimates; however, all three methods exhibited underprediction after approximately 1.5 weeks, with method 2 showing the closest approximation.

Since method 3 more effectively captured the rapid initial increase in cumulative probability (reflecting event clustering) during the first approximately 1.5 weeks than the other methods, the Weibull model alone appeared sufficient to adequately describe the data, without requiring the additional steepness in slope provided by the log-logistic model.

As shown in Figure 4c, the simulation curve from method 1 consistently underpredicted the KM curve throughout the observation period. In contrast, method 2 closely approximated the KM curve up to approximately 1.5 weeks, after which it shifted toward underprediction, similar to the pattern observed with the Weibull model. Method 3 exhibited overprediction during the initial week but showed good agreement with the KM curve up to approximately 5 weeks, followed by a shift toward underprediction thereafter. Among the log-logistic models, method 3 provided the closest fit to the KM curve.

Similar to the Weibull model, the log-logistic model is characterized by two parameters but offers greater flexibility in capturing sharp changes in the hazard observed in the data (Kleinbaum and Klein, 2012). This flexibility manifested differently across methods. During the early phase up to approximately 1.5 weeks, where adverse events were clustered, method 2 showed closer agreement with the KM curve than method 3, in contrast to the Weibull model. This suggests that the flexibility of the log-logistic model compensates for the limited ability of method 2 to fully capture time-varying hazard patterns.

In the early phase of the log-logistic model, method 3 exhibited overprediction relative to the KM curve, which may be attributed to its incorporation of cumulative hazard effects that already capture the rapid increase in cumulative event probability. Consequently, the additional flexibility provided by the log-logistic model appears to be redundant, potentially contributing to the observed overprediction in the early phase.

Overall, when jittered AE data were fitted to three base models (Supplementary File 3), none demonstrated satisfactory concordance in the VPCs between the model-predicted cumulative probability of AEs and the nonparametric KM estimates. This suggests that the data cannot be sufficiently explained by a single base model (Figure 4).

Improved fitting can be achieved by incorporating covariates, adopting a hybrid model in the same method aligned with the median of the KM curve, or selecting a model with a high overall overlap within the confidence interval of the KM curve. Method 3 exemplifies such a hybrid approach, employing the Weibull model up to 1.5 weeks and the log-logistic model, thereafter, thereby aligning closely with the KM median. Moreover, method 2 demonstrated a high overlap between the lower bound of the confidence interval in the KM curve and the upper bound of the prediction interval in the models, while exhibiting a consistent underprediction bias, with predicted event probabilities approximately 0.1 lower than the observed values.

Additionally, this dataset exhibited a different pattern from the rat data analyzed earlier. A very steep cumulative hazard and probability slope was observed immediately after the first administration and during the early period, driven by a large number of events. Subsequently, the slope gradually flattened toward the midpoint of the observation period (week 14).

A detailed analysis integrating the methodological characteristics of the Weibull and log-logistic model fitting results for the jittered AEs with the model-specific properties of each method is presented in Supplementary File 25 (Cook, 2007; Andersen et al., 1993; Fleming and Harrington, 2011; Kleinbaum and Klein, 2012; Lawless, 2003). In the three jittered AE models, Supplementary File 26 presents cumulative probability of experiencing no repeated events. In contrast, Supplementary File 27 depicts the VPCs of the cumulative hazard of repeated events.

#### 3.2.2 Exponential model with covariates: fitting results

Given the inherent nature of AEs, a background hazard independent of drug treatment is always present (Collett, 2015; Leroy et al., 2014). Furthermore, depending on multiple influencing factors—such as drug concentration, cumulative drug exposure (AUC), and non-drug effects (Bonate, 2011)—the incidence of AEs typically peaks shortly after drug administration and subsequently declines (Leroy et al., 2014). Therefore, in applied settings, AE analyses often assume a constant hazard over the observation period and incorporate covariates, such as drug concentration or AUC to improve explanatory power (Collett, 2015; Bonate, 2011).

Following the fitting of the base models for each method, parameters of the exponential model incorporating relevant covariates were estimated. The objective function values obtained with successful minimization for each method are presented in Table 10, and the corresponding simulation plots are shown in Figure 5.

**FIGURE 5:**
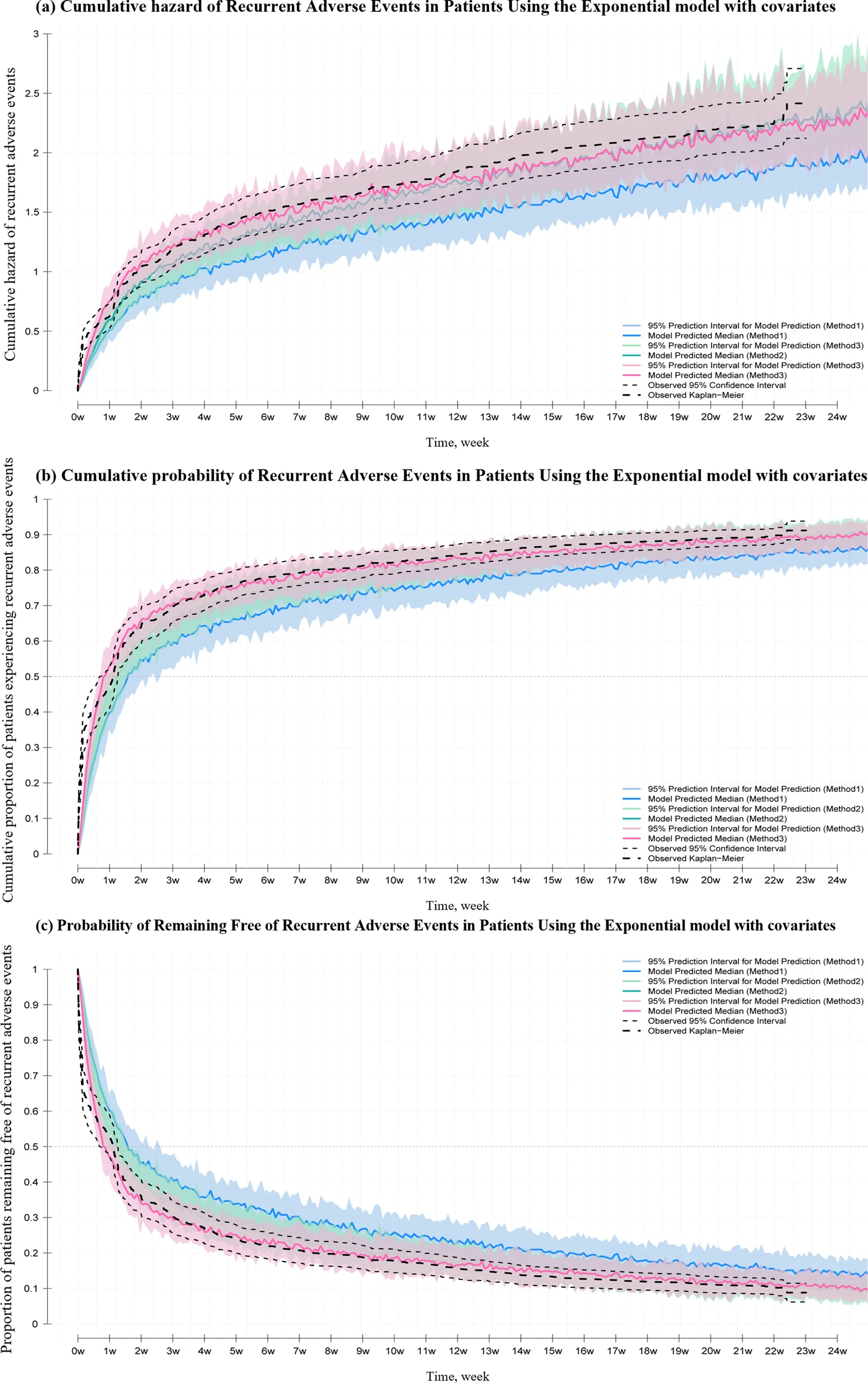
Visual predictive checks (VPCs) of recurrent exact-time events for the exponential model including covariates (jittered adverse event data): **(a)** cumulative hazard of recurrent adverse events in patients; **(b)** cumulative probability of recurrent adverse events in patients; and **(c)** probability of remaining free of recurrent adverse events in patients.

As the analysis of events using the original (non-jittered) dataset will be reported in a separate paper, the present study does not provide the specific code, parameter estimates, or standard errors for the exponential model with covariates.

As direct comparisons of objective function values across methods are not meaningful, visual predictive checks (VPCs) were conducted to evaluate the extent to which the simulation outputs from each method align with the nonparametric Kaplan–Meier (KM) curves (Collett, 2015; Venables and Ripley, 2002).

The exponential model with covariates was selected because it allows the effects of drug concentration, cumulative drug exposure (AUC), and non-drug effects to be evaluated separately over the entire observation period. In contrast, when Weibull or log-logistic models are applied, the estimated base model parameters tend to absorb covariate effects, including both drug-related and non-drug-related factors, making it difficult to disentangle their individual contributions to outcomes such as tumor recurrence or adverse events. Accordingly, comparisons across methods were conducted exclusively using the exponential model with covariates, without employing Weibull or log-logistic models.

Compared to a nonparametric method, fitting the exponential model with both drug-related and non-drug covariates showed that method 1 produced the largest degree of underprediction. Method 2 yielded results slightly closer to the nonparametric estimates than method 1, with the upper bound of the simulated VPC overlapping the lower bound of the confidence interval in the KM plot, although the overall prediction remained below the KM estimates.

Method 3 demonstrated the most favorable visual predictive check (VPC) results among the three methods. It showed slight underprediction during the initial steep interval (∼0.5 weeks), after which the simulated curve aligned closely with the median of the KM estimates. These results likely reflect the greater flexibility of method 3 in representing the conditional hazard. Specifically, by approximating the conditional hazard as the average rate of change in the cumulative hazard, method 3 was able to more effectively capture the underlying event dynamics, particularly in the early phase where events are highly clustered.

## 4 Discussion

### 4.1 Summary of probabilistic structures across methods

In this study, we systematically examined and reformulated the probabilistic structures underlying three parametric approaches (methods 1–3) for repeated time-to-event (TTE) analysis and evaluated their characteristics through comparison with nonparametric Kaplan–Meier (KM) estimates (Cook, 2007; Amorim and Cai, 2015).

Method 1 defines the survival and hazard functions independently based on gap time, treating repeated events as a renewal process and thus not accounting for the influence of prior events. Method 2 preserves calendar time and incorporates partial dependence between events through a conditional survival framework; however, the hazard remains defined as an instantaneous quantity, limiting its ability to reflect accumulated risk. In contrast, the proposed method 3 defines the conditional hazard as the rate of change in cumulative hazard over event intervals, enabling a coherent formulation of both survival and hazard within a conditional probabilistic framework and providing a more consistent representation of inter-event dependence (Cook, 2007; Andersen et al., 1993; Fleming and Harrington, 2011; Kalbfleisch and Prentice, 2002).

### 4.2 Bridging structural differences between KM-based and parametric hazard representations

Parametric modeling estimates parameters at the individual level by sequentially updating likelihood contributions based on each subject’s event history, whereas the KM approach estimates event probabilities at the population level using aggregated event counts within each time interval. Consequently, these two approaches differ fundamentally in how event information is accumulated and represented (Kaplan and Meier, 1958; Wang and Chang, 1999).

In parametric approaches, hazard increments are effectively averaged over individual-level event time distributions, which may smooth out the sharp and localized increases in risk observed in KM curves. This discrepancy becomes more pronounced under decreasing hazard patterns with early event clustering, where steep cumulative hazard trajectories are observed (Cook, 2007; Andersen et al., 1993).

In this context, the proposed method 3 adopts an interval-averaged conditional hazard corresponding to the expected number of events within each interval from a counting process perspective. This formulation is mathematically aligned with the way nonparametric approaches implicitly average hazard within intervals. As a result, method 3 can preserve individual-level parametric modeling while more faithfully reproducing population-level KM curves, as demonstrated by improved agreement in visual predictive checks (VPCs) (Andersen et al., 1993; Fleming and Harrington, 2011).

### 4.3 Mathematical basis and compensatory role of method 3 in complex and relatively inflexible parametric models

In parametric analyses, model structures can become complex with the inclusion of covariates yet may still struggle to adequately capture the steepness of KM curves; in particular, exponential models with covariates (e.g., concentration and AUC) may have inherent structural limitations in representing the curvature of the cumulative hazard function. The proposed method 3 addresses this limitation by defining the conditional hazard as an interval-averaged quantity. This formulation allows repeated events to be interpreted as a counting process with time-varying intensity, linking the hazard within each interval to the expected number of events (Andersen et al., 1993; Fleming and Harrington, 2011).

In particular, pharmacokinetic covariates (e.g., concentration and AUC) typically change rapidly immediately after dosing, further accentuating the concavity of the cumulative hazard function in early phases. As a result, the residual term (derived from the Taylor series expansion in Section 2.5 and Supplementary File 17) becomes positive, leading method 3 to yield systematically larger hazard estimates than method 2.

Consequently, method 3 is better able to capture the dynamic features of the data, particularly the steep early-phase increases observed in KM curves. Therefore, method 3 can help address the limitations of covariate-based exponential models in capturing complex and rapidly changing hazard patterns and may serve as a useful alternative not only in such settings but also in more complex parametric models where steep KM trajectories are not adequately reproduced (Bonate, 2011).

### 4.4 Practical considerations and limitations in applying method 3

The proposed method 3 may exhibit overprediction in certain intervals under decreasing hazard patterns, which can be interpreted as a structural consequence of its cumulative hazard-based conditional formulation. In particular, when events are densely clustered in the early phase, this effect may be amplified in combination with highly flexible parametric models, leading to an overall increase in the estimated cumulative risk. This behavior reflects an inherent trade-off associated with the structural properties of method 3. Accordingly, model selection should be guided by the underlying hazard patterns and event distribution, with an approach that not only selects an appropriate model but also incorporates complementary methods to address the limitations of each modeling framework.

In addition, method 3 defines the conditional hazard using the time interval ( Δt ) between successive events. As a result, numerical instability may arise in the presence of tied events, where multiple events occur at the same time point. To address this issue, a small-time jittering strategy(0.1h) was applied in this study, which enabled stable parameter estimation and convergence within the NONMEM® framework. These findings suggest that, with appropriate preprocessing, the advantages of method 3 can be effectively utilized in real-world clinical datasets. Nevertheless, further work is warranted to develop more robust numerical stabilization approaches for broader applicability (Bauer et al., 2022).

### 4.5 Uncertainty and structural limitations of KM-based estimates

An important but often overlooked consideration in the evaluation of repeated event models is that the Kaplan–Meier (KM) estimator, which is widely used as a reference in visual predictive checks (VPCs), inherently involves both statistical uncertainty and structural limitations. As described in Section 2.1, KM-based extensions for recurrent events reset the time origin at each event and construct product-limit estimates by approximating inter-event intervals as independent binomial processes. This formulation does not fully capture the underlying probabilistic structure of recurrent event processes, particularly the dependence between successive events (Kaplan and Meier, 1958; Wang and Chang, 1999).

Moreover, while KM estimates may be relatively stable in early intervals with a large number of events, their uncertainty increases substantially in later intervals as the risk set diminishes, leading to wider confidence intervals. Therefore, discrepancies between parametric model predictions and KM estimates should not be interpreted solely as evidence of model misspecification but rather should be considered in light of both the structural characteristics of the data and the inherent limitations of the KM estimator (Kaplan and Meier, 1958; Wang and Chang, 1999).

### 4.6 Practical guidance for method selection based on data characteristics

The findings of this study suggest that no single method is universally optimal for recurrent event analysis; rather, method selection should be guided by the underlying data characteristics. While all three approaches demonstrated comparable predictive performance under relatively stable hazard conditions, real-world clinical data often exhibit complex, time-varying hazard dynamics.

When considering decreasing hazard patterns, the scenarios can be broadly categorized as follows.

In scenarios where events are sparse and the hazard changes gradually over time, method 2 may provide a reasonable approximation to nonparametric estimates while offering computational efficiency. In contrast, when early events are clustered—such as immediately following drug administration—or when strong dependence exists between events, resulting in rapidly changing hazard functions, models with sufficient inherent flexibility (e.g., log-logistic or Weibull base models) may still allow method 2 to perform reasonably well; however, underestimation may occur in such settings. In rapidly changing hazard settings, method 3, with its greater flexibility, can provide a more appropriate framework by better capturing these dynamic hazard patterns.

This advantage becomes more pronounced in structurally complex models that incorporate multiple covariates but remain limited in flexibility—such as exponential models with covariates—where method 3 can more effectively accommodate complex, time-varying hazard dynamics and yield improved agreement with nonparametric KM-based estimates. A summary of these practical considerations is provided in Table 8.

## 5 Limitations and future directions

This study has several limitations. First, a systematic sensitivity analysis was not conducted to quantitatively compare differences among the three methods across diverse data scenarios. In particular, factors such as event density, the magnitude of inter-individual variability, and the proportion of right censoring may substantially influence model performance. Our research was based on two datasets—a rat tumor recurrence dataset and a jittered AE dataset—and comparisons were primarily performed using VPCs. As such, the findings may be specific to the decreasing hazard patterns and structural characteristics shown in these datasets. Future research should incorporate simulation-based sensitivity analyses by varying the key factors such as event density, observation period, hazard dynamics, and covariate structures. Such analyses provide a more rigorous basis for refining the method selection guidelines presented in Table 8.

Moreover, external validation using datasets from multiple therapeutic areas and diverse event patterns encountered in real-world clinical settings can be performed to assess the generalizability of method 3. In doing so, datasets with scenarios in which recurrent events are accompanied by terminal events or competing risks should also be included since these occur in real-world clinical settings. In such settings, joint modeling frameworks are required to account for the dependence between recurrent and terminal events, and similarly, distinct modeling approaches are needed to describe competing risk scenarios. Such modeling frameworks may be further developed to improve generalizability (Ghosh and Lin, 2000; Rauch et al., 2018).

Furthermore, the present study primarily focused on decreasing hazard patterns, and systematic evaluation under increasing or more complex hazard dynamics was not conducted. Although decreasing hazards are common in many clinical contexts, scenarios with increasing or non-monotonic hazard patterns may arise, and the relative performance of the proposed methods under such conditions remains to be fully characterized. Such complex data structures fall outside the scope of the current study and should be addressed in future research to extend the applicability of the proposed methods (Ghosh and Lin, 2000).

Lastly, the present study (Part I) only focuses on recurrent event analyses with exact event times within the NONMEM® framework, while interval-censored events will be addressed separately in Part II. In practical applications, recurrent event data in NONMEM® are typically analyzed using exact event times, interval-censored events, right-censored observations, and time scales based on either gap time or calendar time. However, under alternative modeling frameworks, other data structures may also arise, including left censoring, mixed censoring, or irregular follow-up schedules with varying observation intervals. These more complex data configurations were not considered in the present study and should be considered in future research to evaluate the applicability and performance of the proposed methods under these alternative data structures.

## 6 Conclusion

Recurrent time-to-event (RTTE) analysis has been increasingly applied across diverse therapeutic areas, including hemophilia, hereditary angioedema, and pharmacovigilance (Wong et al., 2025; Garcia et al., 2025; Goulooze et al., 2026), underscoring the growing importance of selecting appropriate modeling frameworks for such data. In conclusion, the findings of this study highlight that no single method is universally optimal for recurrent time-to-event modeling, and that method selection should be guided by the underlying hazard patterns and data characteristics. Under constant hazard conditions, methods 1–3 yield equivalent results and can be used interchangeably. In contrast, under decreasing hazard patterns, the choice between methods becomes more consequential. When events are sparse and hazard changes are gradual, method 2 provides a practical and computationally efficient approach with good agreement to nonparametric estimates.

In settings characterized by early event clustering and rapidly changing hazard dynamics—particularly when parametric models lack sufficient flexibility to capture such patterns—method 3 emerges as an appropriate approach, as it more effectively captures steep cumulative hazard trajectories. For example, when covariate-augmented exponential models fail to adequately represent the temporal shape of the hazard, the interval-based conditional hazard formulation of method 3 provides a preferred modeling strategy. Accordingly, an integrated modeling framework that combines appropriate method selection with complementary methodological approaches is recommended to better capture the complexity of recurrent event data in clinical applications.

## Data Availability

The raw data supporting the conclusions of this article will be made available by the authors, without undue reservation.

## References

[B1] Aalen O. O. Borgan Ø. Gjessing H. K. (2008). Survival and event history analysis: a process point of view. New York (NY): Springer.

[B2] Amorim L. D. Cai J. (2015). Modelling recurrent events: a tutorial for analysis in epidemiology. Int. J. Epidemiol. 44 (1), 324–333. 10.1093/ije/dyu222 25501468 PMC4339761

[B3] Andersen P. K. Borgan Ø. Gill R. D. Keiding N. (1993). Statistical models based on counting processes. New York (NY): Springer-Verlag.

[B4] Apostol T. M. (1974). Mathematical analysis. 2nd ed. Reading (MA): Addison-Wesley.

[B5] Audenaert K. M. R. Aujla J. S. (2011). On norm sub-additivity and super-additivity inequalities for concave and convex functions. arXiv [Preprint]. arXiv:1012.2254. Available online at: https://arxiv.org/abs/1012.2254 (Accessed May 4, 2026).

[B6] Bauer R. J. Beal S. L. Sheiner L. B. Bockmann A. J. (2022). NONMEM user’s guides: introduction to NONMEM 7, 5.1. Gaithersburg (MD): ICON plc.

[B7] Bonate P. L. (2011). Pharmacokinetic–pharmacodynamic modeling and simulation. 2nd ed. New York (NY): Springer.

[B8] Boyd S. Vandenberghe L. (2004). Convex optimization. Cambridge (UK): Cambridge University Press.

[B9] Collett D. (2015). Modelling survival data in medical research. 3rd ed. Boca Raton (FL): Chapman and Hall/CRC.

[B10] Conrad K. (2010). The remainder in taylor series. Storrs, CT: University of Connecticut. Available online at: https://kconrad.math.uconn.edu/blurbs/analysis/TaylorRemainder.pdf (Accessed May 04, 2026).

[B11] Cook R. J. (2007). The statistical analysis of recurrent events. New York (NY): Springer.

[B12] DataCamp (2026). Weibull distribution: how to model time-to-event data. Available online at: https://www.datacamp.com/tutorial/weibull-distribution (Accessed October 31, 2025).

[B13] D’Arrigo G. Leonardis D. Abd ElHafeez S. Fusaro M. Tripepi G. Roumeliotis S. (2021). Methods to analyse time-to-event data: the kaplan–meier survival curve. Oxid. Med. Cell Longev. 2021, 2290120. 10.1155/2021/2290120 34594473 PMC8478547

[B14] epiR package documentation (2026). Event instantaneous hazard based on kaplan–meier survival estimates [R package epiR version 2.0.75]. Available online at: https://search.r-project.org/CRAN/refmans/epiR/html/epi.insthaz.html (Accessed May 04, 2026).

[B15] Fleming T. R. Harrington D. P. (2011). Counting processes and survival analysis. New York (NY): John Wiley and Sons.

[B16] Garcia R. Cheng S. Glassman F. Sharma A. De Miguel-Lillo B. Wiens M. (2025). Population pharmacokinetic/pharmacodynamic and exposure–response modeling of garadacimab in healthy volunteers and patients with hereditary angioedema. CPT Pharmacometrics and Syst. Pharmacol. 14 (5), 954–963. 10.1002/psp4.70009 40042097 PMC12072213

[B17] Ghosh D. Lin D. Y. (2000). Nonparametric analysis of recurrent events and death. Biometrics 56 (2), 554–562. 10.1111/j.0006-341X.2000.00554.x 10877316

[B18] Goulooze S. C. Snelder N. Garmann D. (2026). Impact of event-related treatment modifications in exposure-response analyses of repeated time-to-event data. CPT Pharmacometrics and Syst. Pharmacol. 15 (3), e70231. 10.1002/psp4.70231 PMC13097640 41817383

[B19] Greenwood M. (1926). The natural duration of cancer. Rep. Public Health Med. Subj. 33, 1–26.

[B20] Kalbfleisch J. D. Prentice R. L. (2002). The statistical analysis of failure time data. 2nd ed. Hoboken (NJ): John Wiley and Sons.

[B21] Kaplan E. L. Meier P. (1958). Nonparametric estimation from incomplete observations. J. Am. Stat. Assoc. 53 (282), 457–481. 10.1080/01621459.1958.10501452

[B22] Kleinbaum D. G. Klein M. (2012). Survival analysis: a self-learning text. New York (NY): Springer.

[B23] Knopp K. (1951). Theory and application of infinite series. London (UK): Blackie and Son.

[B24] Lavielle M. (2014). Mixed effects models for the population approach. Boca Raton (FL): Chapman and Hall/CRC.

[B25] Lawless J. F. (2003). Statistical models and methods for lifetime data. 2nd ed. Hoboken (NJ): Wiley.

[B26] Leroy F. Dauxois J. Y. Thophile H. Haramburu F. Tubert-Bitter P. (2014). Estimating time-to-onset of adverse drug reactions from spontaneous reporting databases. BMC Med. Res. Methodol. 14, 17. 10.1186/1471-2288-14-17 24490673 PMC3923259

[B27] LibreTexts (2023). “Concavity and the second derivative,” in Calculus. 3rd ed. Available online at: https://math.libretexts.org/Bookshelves/Calculus/Calculus_3e_%28Apex%29/03%3A_The_Graphical_Behavior_of_Functions/3.04%3A_Concavity_and_the_Second_Derivative (Accessed May 04, 2026).

[B28] Lim H. S. (2021). Brief introduction to parametric time-to-event models. Transl. Clin. Pharmacol. 29 (1), 1–5. 10.12793/tcp.2021.29.e7 33854996 PMC8020361

[B29] Miller S. J. (2016). Intermediate and mean value theorems and taylor series. Williamstown, MA: Williams College. Available online at: https://web.williams.edu/Mathematics/sjmiller/public_html/BrownClasses/90/MVT_TaylorSeries.pdf (Accessed May 04, 2026).

[B30] NCSS Statistical Software (2026). Distribution (Weibull) fitting. Available online at: https://www.ncss.com/wp-content/themes/ncss/pdf/Procedures/NCSS/Distribution-Weibull-Fitting.pdf (Accessed May 04, 2026).

[B31] Nelson W. (1982). Applied life data analysis. New York (NY): Wiley.

[B32] OpenStax (2022). Taylor and Maclaurin Series, Vol. 2. Houston (TX): Rice University. Available online at: https://openstax.org/books/calculus-volume-2/pages/6-3-taylor-and-maclaurin-series (Accessed May 04, 2026).

[B33] Pawitan Y. (2001). In all likelihood: statistical modelling and inference using likelihood. Oxford (UK): Oxford University Press.

[B34] Rauch G. Kieser M. Binder H. Bayes-Genis A. Jahn-Eimermacher A. (2018). Time-to-first-event *versus* recurrent-event analysis: points to consider for selecting a meaningful analysis strategy in clinical trials with composite endpoints. Clin. Res. Cardiol. 107 (5), 437–443. 10.1007/s00392-018-1205-7 29453594

[B35] Rosen K. H. (2012). Discrete mathematics and its applications. 7th ed. New York (NY): McGraw-Hill.

[B36] Rudin W. (1976). Principles of mathematical analysis. 3rd ed. New York (NY): McGraw-Hill.

[B37] Singer J. D. Willett J. B. (2003). Applied longitudinal data analysis: modeling change and event occurrence. Oxford (UK): Oxford University Press, 348.

[B38] Therneau T. M. (2023a). A package for survival analysis in R (Version dated August 13, 2023). R package vignette, survival package. Available online at: https://ftp2.uib.no/cran/web/packages/survival/vignettes/survival.pdf (Accessed September 8, 2023).

[B39] Therneau T. M. (2023b). Survival analysis [R package version 3.5-5]. Available online at: https://CRAN.R-project.org/package=survival (Accessed May 04, 2026).

[B40] Therneau T. M. Grambsch P. M. (2000). “Ordered multiple events,” in Modeling survival data: extending the cox model (New York (NY): Springer), 173–219. 10.1007/978-1-4757-3294-8_8

[B41] Venables W. N. Ripley B. D. (2002). Modern applied statistics with S. 4th ed. New York (NY): Springer, 353–385.

[B42] Wang M. C. Chang S. H. (1999). Nonparametric estimation of a recurrent survival function. J. Am. Stat. Assoc. 94 (447), 146–153. 10.1080/01621459.1999.10473831 24244058 PMC3826567

[B43] Wong C. P. Bhagunde P. Nyberg J. Katragadda S. Demissie M. Willemze A. (2025). Efanesoctocog alfa population pharmacokinetics and repeated time-to-event analysis of bleeds in adults, adolescents, and children with severe hemophilia A. J. Clin. Pharmacol. 65, 860–872. 10.1002/jcph.70008 40123211 PMC12202194

