# Supplementary material for: The refinement of repeated exact time-to-event modeling: methodological refinements and implementation in NONMEM® —part I
Source: Front Pharmacol. 2026 Sep 14;17:1791423. doi: 10.3389/fphar.2026.1791423 (PMC13617276; doi:10.3389/fphar.2026.1791423)
Supplement: Supplementary file 1 [file Supplementaryfile1.docx]

**Supplementary 1. Definitions and key equations for a single exact time-to-event** [1, 12-13]

Survival analysis is a representative example of a time-to-event analysis. Following drug administration, a survival curve can be constructed from population-level data to determine whether a patient remains alive or has experienced death. In this context, death represents the event of interest, and the survival curve denotes the cumulative probability that the first event has not occurred by a given time $t$ [1, 13]. $T$ denotes the event time measured from the start of observation, whereas $t_{0}$ denotes the fixed time origin (baseline) at the start of the monitoring period. The survival function represents the cumulative probability of not experiencing the first event up to time $t$, and the corresponding survival curve constitutes a fundamental element in the parametric analysis [1, 13].

The survival function is intrinsically linked to the hazard function, which characterizes the instantaneous risk of the event occurring at a specific time point [1]. The hazard, denoted by $h\left( t \right)$, is mathematically expressed in Equation (Eq.) S1, which also defines the conditional probability that the event occurs instantaneously at time $t$, given that the event has not occurred up to the time $t$ [1]. This function quantifies the instantaneous risk of an event at time $t$ [1].

$$h\left( t \right)=P\left( T=t|T\geq t \right)=\frac{f(t)}{S(t)}=-\frac{d}{dt}\left\{ logS\left( t \right) \right\}=-\frac{1}{S(t)} \lim_{\Delta t\to0} \frac{S(t+\Delta t)-S(t)}{\Delta t}$$

Eq. S1

The cumulative hazard $H\left( t \right)$ quantifies the accumulated hazard of an event occurring by time $t$ [1, 13]. Moreover, $H\left( t \right)$ can be interpreted as the expected number of events occurring during the interval from the time origin $t_{0}$ to $t$ when repetitions of the event are possible [1].

$$H\left( t \right)=\int_{t_{0}}^{t} P\left( T=u|T\geq u \right)du=\int_{t_{0}}^{t} \lim_{\delta t\to0}\frac{P\left( u\leq T<u+\delta t|T\geq u \right)}{\delta t}du$$

$$=\int_{t_{0}}^{t} \left\{ -\frac{1}{S(u)} \lim_{\delta t\to0} \frac{S(u+\delta t)-S(u)}{\delta t} \right\}du=\int_{t_{0}}^{t} h\left( u \right)du$$

Eq. S2

The survival function, $S\left( t \right)$, describes the cumulative probability that the first event has yet to occur by time $t$ from $t_{0}$ [1]:

$$S\left( t \right)=P\left( T>t \right)={exp}^{\left[ -\int_{t_{0}}^{t} h\left( u \right)du \right]}={exp}^{-H(t)}$$

Eq. S3

The cumulative probability function, $F\left( t \right)$, provides the cumulative probability of the first event occurring by time $t$ [1]:

$$F\left( t \right)=P\left( T\leq t \right)=1-S\left( t \right)=1-{exp}^{-H\left( t \right)}=\int_{t_{0}}^{t} f\left( u \right)du$$

Eq. S4

The probability density function (PDF), $f\left( t \right)$, is obtained by differentiating $F\left( t \right)$. Since $F\left( t \right)$ denotes the cumulative probability of the first occurrence of a single event, the derivative of $F\left( t \right)$, $f\left( t \right)$, specifically characterizes the probability density of that single event occurring at an exact time point [1, 13].

$$f\left( t \right)=P\left( T=t \right)=\frac{d}{dt}F\left( t \right)=\frac{d}{dt}\left( 1-S\left( t \right) \right)=-\frac{dS\left( t \right)}{dt}=-\frac{d\left\{ {exp}^{-H\left( t \right)} \right\}}{dt}=S\left( t \right)\cdot h\left( t \right)$$

Eq. S5

Eq. S5 can be reformulated conditionally for an event occurring after the fixed baseline time point $t_{0}$ [1, 12–13]. For $t\geq t_{0}$, the conditional survival function is defined as $S\left( t|t_{0} \right)=\frac{S\left( t \right)}{S\left( t_{0} \right)}$, and the corresponding conditional cumulative hazard is $H\left( t\mid t_{0} \right)=H\left( t \right)-H\left( t_{0} \right)$ [12]. The conditional probability density function is then defined as follows.

$$f\left( t|t_{0} \right)=P\left( T=t|T>t_{0} \right)=\frac{d}{dt}F\left( t|t_{0} \right)=\frac{d}{dt}\left( 1-S\left( t|t_{0} \right) \right)=-\frac{dS\left( t|t_{0} \right)}{dt}=-\frac{d\left\{ {exp}^{-H\left( t|t_{0} \right)} \right\}}{dt}$$

$$=S\left( t|t_{0} \right)\cdot h\left( t|t_{0} \right)=S\left( t|t_{0} \right)\cdot h\left( t \right)$$

Eq. S6

**Supplementary 2. Summary of definitions and key equations for a single exact time-to-event** [1, 12-13]

| **Type of function** | **Definitions and key equations for a single exact time-to-event** |
| --- | --- |
| Hazard function [1, 13] | The conditional probability that the event occurs instantaneously at time $t$, given that the event has not occurred up to the time $t.$  The instantaneous rate at which the event occurs at time $t$, conditional on survival (or event avoidance) up to $t$. |
|  | $h\left( t \right)=P\left( T=t\vert T\geq t \right)=\frac{f(t)}{S(t)}=-\frac{d}{dt}\left\{ logS\left( t \right) \right\}=-\frac{1}{S(t)} \lim_{\delta t\to0} \frac{S(t+\delta t)-S(t)}{\delta t}$ |
| Cumulative hazard function [1, 13] | The cumulative hazard of an event occurring by time $t$.  The expected number of events occurring during the interval from the time origin $t_{0}$ to $t$ when repetitions of the event are possible. |
|  | $H\left( t \right)=\int_{t_{0}}^{t} P\left( T=u\vert T\geq u \right)du=\int_{t_{0}}^{t} \lim_{\delta t\to0}\frac{P\left( u\leq T<u+\delta t\vert T\geq u \right)}{\delta t}du=\int_{t_{0}}^{t} \left\{ -\frac{1}{S(u)} \lim_{\delta t\to0} \frac{S(u+\delta t)-S(u)}{\delta t} \right\}du=\int_{t_{0}}^{t} h\left( u \right)du$ |
| Survival function [1] | The cumulative probability that the first event has yet to occur by time $t$.  The cumulative probability that no event occurs up to time $t$*.* |
|  | $S\left( t \right)=P\left( T>t \right)={exp}^{\left[ -\int_{t_{0}}^{t} h\left( u \right)du \right]}={exp}^{-H(t)}=1-F\left( t \right)=1-\int_{t_{0}}^{t} f\left( u \right)du$ |
| Cumulative probability function [1] | The cumulative probability of the first event occurring by time $t$. |
|  | $F\left( t \right)=P\left( T\leq t \right)=1-S\left( t \right)=1-{exp}^{-H\left( t \right)}=\int_{t_{0}}^{t} f\left( u \right)du$ |
| Probability density function [1, 12-13] | The probability density of the first event occurring at time $t,$  under the assumption that exactly one event occurs after the baseline time $t_{0}$(fixed constant). |
|  | The instantaneous rate at which the cumulative probability of the first event increases at time $t$,  under the assumption that exactly one event occurs after the baseline time $t_{0}$(fixed constant). |
|  | $f\left( t \right)=P\left( T=t \right)=\frac{d}{dt}F\left( t \right)=\frac{d}{dt}\left( 1-S\left( t \right) \right)=-\frac{dS\left( t \right)}{dt}=-\frac{d\left\{ {exp}^{-H\left( t \right)} \right\}}{dt}=S\left( t \right)\cdot h\left( t \right)$ |
|  | $f\left( t\vert t_{0} \right)=P\left( T=t\vert T>t_{0} \right)=\frac{d}{dt}F\left( t\vert t_{0} \right)=\frac{d}{dt}\left( 1-S\left( t\vert t_{0} \right) \right)=-\frac{dS\left( t\vert t_{0} \right)}{dt}=-\frac{d\left\{ {exp}^{-H\left( t\vert t_{0} \right)} \right\}}{dt}=S\left( t\vert t_{0} \right)\cdot h\left( t\vert t_{0} \right)=S\left( t\vert t_{0} \right)\cdot h\left( t \right)$ |

**Supplementary 3. Parametric functions of exponential, Weibull, and log-logistic models** [1-2]

| **Model type** | **Survival function** $S\left( t \right)$ | **Cumulative hazard function** $H\left( t \right)$ | **Hazard function** $h\left( t \right)$ |
| --- | --- | --- | --- |
| Exponential model | $S\left( t \right)=e^{(-\lambda t)}$ | $H\left( t \right)=\lambda t$ | $h\left( t \right)=\lambda$ |
| Weibull model | $S\left( t \right)=e^{\{-({\lambda t)}^{\gamma}\}}$ | $H\left( t \right)=({\lambda t)}^{\gamma}$ | $h\left( t \right)=\gamma({\lambda t)}^{\gamma-1}\lambda=\gamma\lambda^{\gamma}t^{\gamma-1}$ |
| Log-logistic model | $S\left( t \right)=\frac{1}{1+({\frac{t}{\lambda})}^{\gamma}}$ | $H\left( t \right)=log\left\{ 1+\left( \frac{t}{\lambda} \right)^{\gamma} \right\}$ | $h\left( t \right)=\frac{\left( \frac{\gamma}{\lambda} \right)\left( \frac{t}{\lambda} \right)^{\gamma-1}}{1+\left( \frac{t}{\lambda} \right)^{\gamma}}$ |

- $t$: time $(t>0)$
- $\lambda$: model parameter ($\lambda>0$);

rate parameter for the exponential model, inverse-scale parameter for the Weibull model, and scale parameter for the log-logistic model

- $\gamma$: shape parameter $(\gamma>0)$
- $S\left( t \right)$: survival function – probability of surviving beyond time $t$
- $H\left( t \right)$: cumulative hazard – total accumulated hazard up to time $t$
- $h\left( t \right)$: hazard function – instantaneous rate of event at time $t$

**Supplementary 4. Summary of the Kaplan–Meier and Nelson–Aalen estimators for single exact time-to-event data** [1, 8, 14-16]

| **Contents** | **Description** | |
| --- | --- | --- |
| Assumption [1, 15] | 1. Censoring should be non-informative; censored subjects are assumed to have the same subsequent probability of the event as subjects remaining under observation.  2. The exact time of occurrence of the event of interest (e.g., day, month, or year, as applicable) should be available to provide accurate survival estimates.  3. For the interval-based hazard approximation used here, the hazard is assumed to be constant over each inter-event interval.  4. Survival probabilities are assumed to be the same for subjects recruited early and late in the study.  5. Event times are assumed to be independent across subjects. | |
| Definition [8] | The cumulative survival probability that, at a given point in time, the event has not yet occurred. | |
| Type of event [8] | Single event | |
| Characteristics [8] | Cumulative Probability | |
| Risk set [8, 14] | Updated at each event time.  Excludes previous events/censorings. | $n_{i+1}= n_{0}-\sum_{k=0}^{i} \left( e_{k}+c_{k} \right)$ |
| Distribution in each interval [8] | Binomial distribution  The number of subjects with events $X_{i+1}$ | $X_{i+1}\sim Binomial\left( n_{i+1}, p_{i+1} \right)$, $p_{i+1}=\frac{e_{i+1}}{n_{i+1}}$ |
| Piecewise constant (interval-based) hazard estimate, $h\left( t \right)$  [1, 14] | Probability of immediate occurrence of an event per unit of time in each interval | |
|  | $h_{i}(t)=\frac{e_{i+1}}{n_{i+1}\tau_{i+1}}$ *,* $\tau_{i+1}=t_{i+2}-t_{i+1}, t_{i+1}\leq t<t_{i+2}$ | |
| Method | Empirical (Product-limit) | Nelson-Aalen |
| Cumulative hazard function,  derived from the Kaplan–Meier estimator, $H(t)$ [1, 14] | $H\left( t \right)=\sum_{k=0}^{i} \log\left( \frac{n_{k+1}}{n_{k+1}-e_{k+1}} \right) , t_{i+1}\leq t<t_{i+2}$ | $H\left( t \right)=\sum_{k=0}^{i} \frac{e_{k+1}}{n_{k+1}}, t_{i+1}\leq t<t_{i+2}$ |
| Variance of cumulative hazard, $V\left( H\left( t \right) \right)$ [1, 8, 16] | By Normal approximation and Delta method (Greenwood),  $V\left( H\left( t \right) \right)=\sum_{k=0}^{i} \text{\{}\frac{e_{k+1}}{n_{k+1}(n_{k+1}-e_{k+1})}\text{\}}, t_{i+1}\leq t<t_{i+2}$ | By Normal approximation and Delta method (Greenwood),  $V\left( H\left( t \right) \right)=\sum_{k=0}^{i} {\tau_{k+1}}^{2}{h_{k}\left( t \right)}^{2}\frac{(n_{k+1}-e_{k+1})}{n_{k+1}e_{k+1}}, \tau_{k+1}=t_{k+2}-t_{k+1}, t_{i+1}\leq t<t_{i+2}$ |
|  |  | By Poisson approximation, Independence and Additivity of variance  $V\left( H\left( t \right) \right)=\sum_{k=0}^{i} \frac{e_{k+1}}{{n_{k+1}}^{2}}, t_{i+1}\leq t<t_{i+2}$ |
| Survival function, $S\left( t \right)$ [1, 14] | $S\left( t \right)=\prod_{k=0}^{i} \left( \frac{n_{k+1}-e_{k+1}}{n_{k+1}} \right), t_{i+1}\leq t<t_{i+2}$ | $S\left( t \right)=e^{\left\{ -\sum_{k=0}^{i} \frac{e_{k+1}}{n_{k+1}} \right\}}, t_{i+1}\leq t<t_{i+2}$ |
| Variance of the survival function, $V\left( S\left( t \right) \right)$ [1, 8, 16] | By Normal approximation and Delta method (Greenwood),  $V\left( S\left( t \right) \right)={S\left( t \right)}^{2}\sum_{k=0}^{i} \text{\{}\frac{e_{k+1}}{n_{k+1}(n_{k+1}-e_{k+1})}\text{\}},t_{i+1}\leq t<t_{i+2}$ | By Normal approximation and Delta method (Greenwood),  $V\left( S\left( t \right) \right)={S\left( t \right)}^{2}\sum_{k=0}^{i} {\tau_{k+1}}^{2}{h_{k}\left( t \right)}^{2}\frac{(n_{k+1}-e_{k+1})}{n_{k+1}e_{k+1}}, t_{i+1}\leq t<t_{i+2}$ |
|  |  | By Poisson approximation, Independence and Additivity of variance  $V\left( S\left( t \right) \right)={S\left( t \right)}^{2}\sum_{k=0}^{i} \frac{e_{k+1}}{{n_{k+1}}^{2}}, t_{i+1}\leq t<t_{i+2}$ |

where $t_{0}$ denotes the time origin (baseline), $n_{0}$is the initial number of subjects at risk at $t_{0}$, and $e_{0}$ is set to 0 for indexing purposes because no event is assigned to the time origin. Furthermore, $e_{i}$ is the number of events at time $t_{i}$, $n_{i}$ is the number of subjects at risk just prior to $t_{i}$, and $c_{i}$ is the number of censored subjects in the interval $(t_{i}, t_{i+1}]$.

**Supplementary 5. Indexing convention, mathematical formulation, and probabilistic interpretation of the KM-based recurrent-event framework and corresponding parametric extensions**

**(a) Indexing convention for the KM-based recurrent-event framework**

With respect to the calculations illustrated here, the recurrent-event extension follows the same framework as the single-event KM approach, except for risk-set updating: subjects experiencing an event are removed from the subsequent risk set in the single-event framework, whereas they remain eligible for subsequent event intervals in the recurrent-event framework; censored subjects are removed in both frameworks. Apart from this difference in risk-set construction, the same indexing convention is applied to event counts, cumulative-hazard increments, and interval-average hazards.

The figure illustrates the indexing convention used in **Table 1** for the KM-based recurrent-event framework. The cumulative-hazard increment associated with the interval $(t_{i}, t_{i+1}]$ occurs at $t_{i+1}$ and is defined as $\Delta H_{i}=\frac{e_{i+1}}{n_{i+1}}=\frac{e_{i+1}}{n_{i}-c_{i}}$. This increment is subsequently converted to an interval-average hazard, $h_{i}=\frac{\Delta H_{i}}{\tau_{i+1}}=\frac{e_{i+1}}{n_{i+1}\tau_{i+1}}$, and applied over $[t_{i+1}, t_{i+2})$ , where $\tau_{i+1}=t_{i+2}-t_{i+1}$.

The censoring interval and the hazard-application interval use opposite endpoint conventions: $c_{i}$denotes censoring within the left-open, right-closed interval $(t_{i}, t_{i+1}]$, whereas $h_{i}$ is applied over the left-closed, right-open interval $[t_{i+1}, t_{i+2})$. Thus, although $c_{i}$ and $h_{i}$ carry the same subscript $i$, their associated intervals are shifted by one time point because $\Delta H_{i}$, which is associated with $(t_{i}, t_{i+1}]$, occurs at its right endpoint $t_{i+1}$ and is applied as the average hazard over the subsequent interval.

Here, $e_{i+1}$ denotes the number of events at $t_{i+1}$, $n_{i}$ and $n_{i+1}$denote the numbers of subjects at risk immediately before $t_{i}$ and $t_{i+1}$, respectively, and $c_{i}$denotes the number of censored subjects in $(t_{i}, t_{i+1}]$. Red arrows indicate cumulative-hazard increments, and the blue segment indicates the corresponding piecewise-constant interval-average hazard.

**(b) Mathematical formulation and probabilistic interpretation of KM-based and parametric extensions for recurrent event models**

| **Quantity** | **Non-parametric extension**  **(KM-based estimates)** | **Parametric extension** |
| --- | --- | --- |
| Cumulative  Hazard [14] | $\Delta H_{i}=\frac{e_{i+1}}{n_{i+1}}=\frac{e_{i+1}}{n_{i}-c_{i}}$ | $\Delta H_{i}=H\left( t_{i},t_{i+1} \right)=\int_{t_{i}}^{t_{i+1}} h\left( u \right)du$ |
| Survival [13, 17] | $\hat{P}\left( T> t_{i+1}\vert T> t_{i} \right)=\hat{S\left( t_{i}, t_{i+1} \right)}=1-\Delta H_{i}$ | $P\left( T> t_{i+1}\vert T> t_{i} \right)=S\left( t_{i}, t_{i+1} \right)={exp}^{-H(t_{i}, t_{i+1})}$ |
|  |  |  |
| Conditional  cumulative (density)  Probability^1)^  [4, 10, 13, 17] | $\hat{P}\left( t_{i}<T\leq t_{i+1}\vert T> t_{i} \right)=\hat{P}\left( t_{i}<T\leq t_{i+1} \right)$^2)^  $=\hat{F\left( t_{i},t_{i+1} \right)}=1-\hat{S\left( t_{i}, t_{i+1} \right)}=\Delta H_{i}$ | $P\left( t_{i}<T\leq t_{i+1}\vert T> t_{i} \right)=P\left( t_{i}<T\leq t_{i+1} \right)$^2)^  $=F\left( t_{i},t_{i+1} \right)=1-S(t_{i}, t_{i+1})$ |
|  |  |  |
| Conditional  (density)  Probability^3)^  [4, 10-11] | $\hat{P}\left( T=t_{i+1} \mid T>t_{i} \right)$  $=\hat{f\left( t_{i+1} \mid t_{i} \right)}=\frac{\hat{S\left( t_{i} \right)}-\hat{S\left( t_{i+1} \right)}}{\hat{S\left( t_{i} \right)}}=\Delta H_{i}$ | $P\left( T=t_{i+1} \mid T>t_{i} \right)$  $=f\left( t_{i+1} \mid t_{i} \right)=\left. \frac{d}{dt}F\left( t \vert t_{i} \right) \right\vert_{t=t_{i+1}}=\left. \frac{d}{dt}\left\{ -S\left( t \vert t_{i} \right) \right\} \right\vert_{t=t_{i+1}}$  $=h(t_{i+1})\cdot S(t_{i+1}\vert t_{i})$ |
|  |  |  |

Given these conceptual distinctions, to avoid confusion with the symbols used in the parametric context, we denoted both the survival function and the associated probabilities in the nonparametric method using a $\hat{hat}$ notation.

^1)^where $e_{i+1}$ is the number of events at $t_{i+1}$, $n_{i}$ is the number of subjects at risk just prior to $t_{i}$, $n_{i+1}$ is the number of subjects at risk just prior to $t_{i+1}$ and $c_{i}$ is the number of censored subjects within the interval $(t_{i}, t_{i+1}]$ [17]. The detailed mathematical derivation of conditional density probability is provided below.

^2)^Here, because the time origin is reset at $t_{i}$, probabilities over $(t_{i}, t_{i+1}]$ are defined conditionally on $T>t_{i}$. Thus, the condition $T>t_{i}$ is implicit in $P\left( t_{i}<T\leq t_{i+1} \right)$, which denotes the interval probability under the conditional framework rather than an unconditional probability on the original time scale.

^3)^The equivalence between the interval cumulative probability and the conditional cumulative probability is demonstrated below.

**Conditional cumulative probabilities for non-parametric (KM estimates) and parametric methods** [8-9]

By definition of the KM estimates in recurrent event settings,

$$\hat{F\left( t_{i},t_{i+1} \right)}=\hat{P}\left( t_{i}<T\leq t_{i+1} \right)=\hat{P}\left( t_{i}<T\leq t_{i+1}|T>t_{i} \right)=\hat{P}\left( T\leq t_{i+1}|T>t_{i} \right)=\hat{F\left( t_{i+1}|t_{i} \right)}$$

Applying the same definition in the parametric framework yields the corresponding relations

$$F\left( t_{i},t_{i+1} \right)=F\left( t_{i+1}|t_{i} \right)$$

**Probability density and conditional density functions under the KM estimates** [4, 10-11]

Since $\hat{S\left( t \right)}$ is a step function, $\hat{S'\left( t \right)}=0$ within intervals $t_{i}<t<t_{i+1}$ where no events occur. At the event time $t_{i+1}$, the survival probability decreases by a proportion of $\Delta H_{i}$, resulting in a jump of magnitude $\Delta\hat{S\left( t_{i+1} \right)}$. This jump size is equivalent to the corresponding increase in the cumulative distribution function $\hat{F\left( t \right)}$ [4, 10-11].

$$\Delta\hat{S\left( t_{i+1} \right)}=\hat{S\left( t_{i} \right)}-\hat{S\left( t_{i+1} \right)}=\hat{S\left( t_{i} \right)}-\hat{S\left( t_{i} \right)}\cdot\left( 1-\Delta H_{i} \right)=\hat{S\left( t_{i} \right)}\cdot\Delta H_{i}=\Delta\hat{F\left( t_{i+1} \right)}$$

Consequently, the derivative of $\hat{F\left( t \right)}$ at time $t$—the probability density of an event (not restricted to the first occurrence) taking place at time $t$—takes the form of a point density concentrated only at event times [4, 10-11].

$$\hat{f\left( t \right)}=\hat{P}(T=t)=\frac{d}{dt}\hat{F\left( t \right)}=-\frac{d}{dt}\hat{S\left( t \right)}=\sum_{i} (\hat{S\left( t_{i} \right)}-\hat{S\left( t_{i+1} \right)})\delta(t-t_{i+1})$$

Therefore, the value of $\hat{f\left( t \right)}$ at each event can be expressed as follows [4, 10-11].

$$\hat{f\left( t_{i+1} \right)}=\hat{P}(T=t_{i+1})=\hat{S\left( t_{i} \right)}-\hat{S\left( t_{i+1} \right)}$$

In the KM analysis for repeated exact-time events, the time of the previous event $t_{i}$ is treated as a realized constant of the random variable when evaluating the subsequent event time $t_{i+1}$ [4, 10]. Under this formulation, the conditional probability density $\hat{f\left( t|t_{i} \right)}$ has $t$ as its sole variable for differentiation [4, 10-11].

$$\hat{f\left( t|t_{i} \right)}=\frac{d}{dt}\hat{F\left( t|t_{i} \right)}=\frac{d}{dt}\hat{P}\left( T\leq t | T>t_{i} \right)=\hat{P}\left( T=t | T>t_{i} \right)$$

In conclusion, the conditional probability density $\hat{f\left( t_{i+1}|t_{i} \right)}$—that the current event at $t_{i+1}$ occurs after the previous event at $t_{i}$ and no event occurrence within $(t_{i},t_{i+1})$— does not assume the occurrence of only a single event after $t_{i}$ [4, 10-11]. Therefore, $\hat{f\left( t_{i+1}|t_{i} \right)}$ represents the discrete increment of the cumulative hazard between the previous event $\left( t_{i} \right)$ and the current event $\left( t_{i+1} \right)$ [4, 10-11].

Accordingly,

$$\hat{f\left( t_{i+1}|t_{i} \right)}=\hat{P}\left( T=t_{i+1} | T>t_{i} \right)=\frac{\hat{S\left( t_{i} \right)}-\hat{S\left( t_{i+1} \right)}}{\hat{S\left( t_{i} \right)}}=1-\frac{\hat{S\left( t_{i+1} \right)}}{\hat{S\left( t_{i} \right)}}=1-\left( 1-\Delta H_{i} \right)=\Delta H_{i}$$

which corresponds to the hazard increment at $t_{i+1}$ [4, 10-11].

Under the KM framework, the survival function is a step function by definition; therefore, for the interval $(t_{i},t_{i+1}]$, the conditional probability density $\hat{f\left( t_{i+1}|t_{i} \right)}$ is equivalent to the conditional cumulative probability $\hat{F\left( t_{i+1}|t_{i} \right)}$.

**Supplementary 6. Theoretical justification for the comparison of the KM-based cumulative hazard with the parametric** $\boldsymbol{-logS}\left( \boldsymbol{t} \right)$ [1, 8, 21-22]

In the parametric approach, the cumulative hazard is assumed to follow a predefined distribution. Thus, model parameters are estimated based on this assumption and subsequently used to simulate survival probabilities at each time point. The resulting cumulative hazard curves are then compared to those derived from the nonparametric analysis. To provide the theoretical basis for this comparison, the relationship between the KM-based cumulative hazard and survival estimates can be expressed as follows [1, 8]:

$$\hat{H\left( t \right)}= \sum_{t_{i}\leq t} \Delta H_{i}\approx-log\hat{S(t)}$$

The left-hand side represents the cumulative hazard estimated via the nonparametric analysis, whereas the right-hand side represents the negative logarithmic transformation of the corresponding nonparametric survival estimate. This relationship arises from the first-order Taylor approximation $log(1-x)\approx-x$, which holds when $x$ is small [21-22].

$$log\left( 1-x \right)= -x-\frac{x^{2}}{2}-\frac{x^{3}}{3}-\cdots(for small x)$$

Utilizing the above properties, the logarithm of the survival function in the non-parametric method, $log\hat{S\left( t \right)}$, can be expanded as a Taylor series as follows [1, 8].

$$log\hat{S\left( t \right)}= log\left\{ \prod_{i} \left( 1-\Delta H_{i} \right) \right\}=\sum_{i} log(1-\Delta H_{i})\approx\sum_{i} \left( -\Delta H_{i} \right)=-\sum_{i} \Delta H_{i}$$

Indeed, the above expression is obtained by substituting $1-\Delta H_{i}$ for $\left( 1-x \right)$ in the Taylor expansion above. Therefore, the cumulative hazard estimate from the nonparametric analysis can be compared with the negative logarithm of the survival estimate from the parametric analysis.

$$Non-parametric analysis ; \hat{H\left( t \right)}=\sum_{t_{i}\leq t} \Delta H_{i}\approx-log\hat{S\left( t \right)}$$

$$Parametric analysis ; -logS(t)$$

Therefore, the KM-based cumulative hazard estimate can be compared with the parametric cumulative hazard calculated as $-logS(t)$.

**References**

1. Collett D. *Modelling survival data in medical research*. 3rd ed. Boca Raton (FL): Chapman & Hall/CRC; 2015.
2. Lim HS. Brief introduction to parametric time-to-event models. *Transl Clin Pharmacol*. 2021;29(1):1–5. doi: 10.12793/tcp.2021.29.e7
3. Bonate PL. *Pharmacokinetic–pharmacodynamic modeling and simulation*. 2nd ed. New York (NY): Springer; 2011.
4. Cook RJ. *The statistical analysis of recurrent events*. New York (NY): Springer; 2007.
5. Amorim LD, Cai J. Modelling recurrent events: A tutorial for analysis in epidemiology. *Int J Epidemiol*. 2015;44(1):324–333. doi: 10.1093/ije/dyu222
6. Ghosh D, Lin DY. Nonparametric analysis of recurrent events and death. *Biometrics*. 2000;56(2):554–562. doi: 10.1111/j.0006-341X.2000.00554.x
7. Rauch G, Kieser M, Binder H, Bayes-Genis A, Jahn-Eimermacher A. Time-to-first-event versus recurrent-event analysis: Points to consider for selecting a meaningful analysis strategy in clinical trials with composite endpoints. *Clin Res Cardiol*. 2018;107(5):437–443. doi: 10.1007/s00392-018-1205-7
8. Kaplan EL, Meier P. Nonparametric estimation from incomplete observations. *J Am Stat Assoc*. 1958;53(282):457–481. Available from: <https://web.stanford.edu/~lutian/coursepdf/KMpaper.pdf>
9. Wang MC, Chang SH. Nonparametric estimation of a recurrent survival function. *J Am Stat Assoc*. 1999;94(447):146–153. doi: 10.1080/01621459.1999.10473831
10. Andersen PK, Borgan Ø, Gill RD, Keiding N. *Statistical models based on counting processes*. New York (NY): Springer-Verlag; 1993.
11. Fleming TR, Harrington DP. *Counting processes and survival analysis*. New York (NY): John Wiley & Sons; 2011.
12. Kalbfleisch JD, Prentice RL. *The statistical analysis of failure time data*. 2nd ed. Hoboken (NJ): John Wiley & Sons; 2002.
13. Kleinbaum DG, Klein M. *Survival analysis: A self-learning text*. New York (NY): Springer; 2012.
14. Nelson W. *Applied life data analysis*. New York (NY): Wiley; 1982.
15. D’Arrigo G. Methods to analyse time-to-event data: The Kaplan–Meier survival curve. *Oxid Med Cell Longev*. 2021;2021:2290120. doi: 10.1155/2021/2290120
16. Greenwood M. The natural duration of cancer. *Rep Public Health Med Subj*. 1926;33:1–26.
17. Lawless JF. *Statistical models and methods for lifetime data*. 2nd ed. Hoboken (NJ): Wiley; 2003.
18. Therneau TM. A package for survival analysis in R (version 3.x) [R vignette]. 2023. Available from: https://cran.r-project.org/web/packages/survival/vignettes/
19. Therneau TM. *survival*: Survival analysis [R package version 3.5-5]. 2023. Available from: https://CRAN.R-project.org/package=survival
20. Therneau TM, Grambsch PM. Ordered multiple events. In: *Modeling survival data: Extending the Cox model*. New York (NY): Springer; 2000. p. 173–219. doi: 10.1007/978-1-4757-3294-8_8
21. OpenStax. Calculus. Volume 2: Taylor and Maclaurin series. Houston (TX): Rice University; 2022. Available from: [https://openstax.org/books/calculus-volume-2/pages/6-3-taylor-and-maclaurin-series](https://openstax.org/books/calculus-volume-2/pages/6-3-taylor-and-maclaurin-series)
22. Conrad K. The remainder in Taylor series. Storrs (CT): University of Connecticut; 2010. Available from: [https://kconrad.math.uconn.edu/blurbs/analysis/TaylorRemainder.pdf](https://kconrad.math.uconn.edu/blurbs/analysis/TaylorRemainder.pdf)
23. Bauer RJ, Beal SL, Sheiner LB, Bockmann AJ. NONMEM user’s guides: Introduction to NONMEM 7.5.1. Gaithersburg (MD): ICON plc; 2022.
24. Lavielle M. Mixed effects models for the population approach. Boca Raton (FL): Chapman & Hall/CRC; 2014.
25. Aalen OO, Borgan Ø, Gjessing HK. Survival and event history analysis: A process point of view. New York (NY): Springer; 2008.
26. Rudin W. Principles of mathematical analysis. 3rd ed. New York (NY): McGraw-Hill; 1976.
27. Apostol TM. Mathematical analysis. 2nd ed. Reading (MA): Addison-Wesley; 1974.
28. Boyd S, Vandenberghe L. Convex optimization. Cambridge (UK): Cambridge University Press; 2004.
29. Pawitan Y. In all likelihood: Statistical modelling and inference using likelihood. Oxford (UK): Oxford University Press; 2001.
30. Leroy F, Dauxois JY, Thophile H, Haramburu F, Tubert-Bitter P. Estimating time-to-onset of adverse drug reactions from spontaneous reporting databases. *BMC Med Res Methodol*. 2014;14:17. doi: 10.1186/1471-2288-14-17
31. Singer JD, Willett JB. *Applied longitudinal data analysis: Modeling change and event occurrence*. Oxford (UK): Oxford University Press; 2003. p. 348.
32. epiR package documentation. Event instantaneous hazard based on Kaplan–Meier survival estimates [R package epiR version 2.0.75]. Available from: <https://search.r-project.org/CRAN/refmans/epiR/html/epi.insthaz.html>
33. Miller SJ. Intermediate and mean value theorems and Taylor series. Williamstown (MA): Williams College; 2016. Available from: <https://web.williams.edu/Mathematics/sjmiller/public_html/BrownClasses/90/MVT_TaylorSeries.pdf>
34. LibreTexts. Concavity and the second derivative. In: *Calculus*. 3rd ed. 2023. Available from: <https://math.libretexts.org/Bookshelves/Calculus/Calculus_3e_%28Apex%29/03%3A_The_Graphical_Behavior_of_Functions/3.04%3A_Concavity_and_the_Second_Derivative>
35. Audenaert KMR, Aujla JS. On norm sub-additivity and super-additivity inequalities for concave and convex functions. *arXiv* [Preprint]. 2010. Available from: https://arxiv.org/abs/1012.2254
36. Rosen KH. *Discrete mathematics and its applications*. 7th ed. New York (NY): McGraw-Hill; 2012.
37. Knopp K. *Theory and application of infinite series*. London (UK): Blackie & Son; 1951.
38. Venables WN, Ripley BD. *Modern applied statistics with S*. 4th ed. New York (NY): Springer; 2002. p. 353–385.
39. NCSS Statistical Software. Distribution (Weibull) fitting. Available from: <https://www.ncss.com/wp-content/themes/ncss/pdf/Procedures/NCSS/Distribution-Weibull-Fitting.pdf>
40. DataCamp. Weibull distribution: How to model time-to-event data. Available from: <https://www.datacamp.com/tutorial/weibull-distribution>. Accessed October 31, 2025.
41. Wong, C.P. et al. (2025) 'Efanesoctocog alfa population pharmacokinetics and repeated time-to-event analysis of bleeds in adults, adolescents, and children with severe hemophilia A', *The Journal of Clinical Pharmacology*, online ahead of print. doi: 10.1002/jcph.70008
42. Garcia, R., Cheng, S., Glassman, F., Sharma, A., De Miguel-Lillo, B., Wiens, M., Johnston, C., Lawo, J.P., Pragst, I., French, J. and Polhamus, D. (2025) 'Population pharmacokinetic/pharmacodynamic and exposure–response modeling of garadacimab in healthy volunteers and patients with hereditary angioedema', *CPT: Pharmacometrics & Systems Pharmacology*, 14(5), pp. 954–963. doi: 10.1002/psp4.70009
43. Goulooze, S.C., Snelder, N. and Garmann, D. (2026) 'Impact of event-related treatment modifications in exposure-response analyses of repeated time-to-event data', *CPT: Pharmacometrics & Systems Pharmacology*, 15(3), e70231. doi: 10.1002/psp4.70231
