# Supplementary material for: The refinement of repeated exact time-to-event modeling: methodological refinements and implementation in NONMEM® —part I
Source: Front Pharmacol. 2026 Sep 14;17:1791423. doi: 10.3389/fphar.2026.1791423 (PMC13617276; doi:10.3389/fphar.2026.1791423)
Supplement: Supplementary file 2 [file Supplementaryfile2.docx]

**Supplementary 7. NONMEM Control Stream: Method 1**

```
$SUBR ADVAN=13 TOL=6

$MODEL
 COMP=(CUMHAZ)

$PK

 IF (NEWIND.LE.1) THEN
  CUMLAST     = 0
  TIME1       = 0
 ENDIF

 LAMDA	= THETA(1)*EXP(ETA(1))
 GAMMA 	= THETA(2)

 DEL    = 0.000001

$DES

 DELT   = T - TIME1

 H1     = (GAMMA/LAMDA)*((DELT+DEL)/LAMDA)**(GAMMA-1)
 H2     = 1 + ((DELT+DEL)/LAMDA)**GAMMA
 HAZ    = H1/H2

 DADT(1) = HAZ

$ERROR

 DELTIME = TIME - TIME1

 HAZR    = (GAMMA/LAMDA)*((DELTIME+DEL)/LAMDA)**(GAMMA-1)/(1+((DELTIME+DEL)/LAMDA)**GAMMA)

 CUMHAZ  = A(1)
 CUMDIF  = CUMHAZ - CUMLAST
 SURV 	 = EXP(-CUMDIF)

 IF (DV.EQ.0.AND.MDV.EQ.0) THEN
  Y = SURV*HAZR
  TIME1   = TIME
  CUMLAST = CUMHAZ
 ELSE
  TIME1   = TIME1
  CUMLAST = CUMLAST
 ENDIF

 IF (DV.EQ.1) THEN
  Y = EXP(-CUMDIF)
 ENDIF

$THETA
 (0, 0.5)
 (0, 0.1)

$OMEGA 0 FIX

$ESTIMATION PRINT=2 MAXEVALS=9999 METHOD=COND LAPLACE LIKELIHOOD MSFO=RETTE_method1_loglog.MSF

$COV PRINT=E

$TABLE ID TIME DV HAZR CUMHAZ SURV LAMDA GAMMA
        FILE=RETTE_method1_loglog.FIT NOPRINT NOAPPEND ONEHEADER FORMAT=s1PE17.11
```
