# Supplementary material for: The refinement of repeated exact time-to-event modeling: methodological refinements and implementation in NONMEM® —part I
Source: Front Pharmacol. 2026 Sep 14;17:1791423. doi: 10.3389/fphar.2026.1791423 (PMC13617276; doi:10.3389/fphar.2026.1791423)
Supplement: Supplementary file 4 [file Supplementaryfile4.docx]

**Supplementary 9. Mathematical definitions, detailed derivations, and comparative structures of single- and recurrent-event approaches**

**(a) Definitions and detailed mathematical derivations of probability density, survival, and hazard functions for Methods 1–3 in repeated exact time-to-event models**

| **Conditional probability density function** | | |
| --- | --- | --- |
|  | **Method 1** | The probability density that exactly one event occurs at time $\Delta t_{i},$ under the assumption that only one event takes place |
|  |  | The probability density is obtained as the product of  (i) the probability of surviving beyond $\Delta t_{i}=t_{i+1}-t_{i}$ and (ii) the instantaneous risk of experiencing an event at $\Delta t_{i}=t_{i+1}-t_{i}$ |
|  |  | $f_{k}\left( t_{i+1} \vert t_{i} \right)=S\left( \Delta t_{i} \right)\cdot h\left( \Delta t_{i} \right)=P\left( T=\Delta t_{i} \right)=\frac{d}{dt}\left[ P\left( T\leq t \right) \right]_{t=\Delta t_{i}}=\frac{d}{dt}\left[ F(t) \right]_{t=\Delta t_{i}}=f(\Delta t_{i})$ |
|  | **Method 2** | The conditional probability density that exactly one event occurs at $t_{i+1}$, given that the previous event occurred at $t_{i}$ and no event occurred in $(t_{i},t_{i+1})$,  assuming that only a single event occurs after baseline time point $t_{i}$ (the realized value of an event-time random variable, subsequently treated as a fixed constant) |
|  |  | The conditional probability density is obtained as the product of  (i) the probability of surviving past $t_{i+1}$ given $T>t_{i}$ and (ii) the risk of experiencing an event at $t_{i+1}$ |
|  |  | $f_{k}\left( t_{i+1} \vert t_{i} \right)=S\left( t_{i+1} \vert t_{i} \right)\cdot h\left( t_{i+1} \right)=\frac{S\left( t_{i+1} \right)}{S\left( t_{i} \right)}\cdot\left\{ -\frac{S^{'}\left( t_{i+1} \right)}{S\left( t_{i+1} \right)} \right\}=-\frac{S^{'}\left( t_{i+1} \right)}{S\left( t_{i} \right)}=\frac{f\left( t_{i+1} \right)}{S\left( t_{i} \right)}=\frac{P\left( T=t_{i+1} \right)}{P\left( T>t_{i} \right)} =P\left( T=t_{i+1} \vert T>t_{i} \right)$  $=\frac{d}{dt}\left[ P\left( T\leq t \vert T>t_{i} \right) \right]_{t=t_{i+1}}=\frac{d}{dt}\left[ F\left( t \vert t_{i} \right) \right]_{t=t_{i+1}}=f\left( t_{i+1} \vert t_{i} \right)$ |
|  | **Method 3** | The conditional probability density that an event occurs at $t_{i+1}$, given that the previous event occurred at $t_{i}$ and no event occurred in $(t_{i},t_{i+1})$,  while allowing for the possibility of multiple events after $t_{i}$, |
|  |  | The conditional probability density is obtained as the product of  (i) the probability of surviving past $t_{i+1}$ given $T>t_{i}$ and (ii) the interval-averaged risk of experiencing an event at $t_{i+1}$, also conditional on $T>t_{i}$  (averaged risk of experiencing an event over the interval ${(t_{i}, t}_{i+1}]$)  (conditional risk of experiencing the ${(i+1)}^{th}$ event at $t_{i+1}$, incorporating a prior-event risk at $t_{i}$) |
|  |  | $f_{k}\left( t_{i+1} \vert t_{i} \right)=S\left( t_{i+1} \vert t_{i} \right)\cdot h\left( t_{i+1} \vert t_{i} \right)=\frac{S\left( t_{i+1} \right)}{S\left( t_{i} \right)}\cdot\frac{\int_{t_{0}}^{t_{i+1}} h\left( u \right)du-\int_{t_{0}}^{t_{i}} h\left( u \right)du}{t_{i+1}-t_{i}} =\frac{S\left( t_{i+1} \right)}{S\left( t_{i} \right)}\cdot\frac{H\left( t_{i+1} \right)-H\left( t_{i} \right)}{t_{i+1}-t_{i}}=\frac{S\left( t_{i+1} \right)}{S\left( t_{i} \right)}\cdot\frac{-logS\left( t_{i+1} \right)+logS\left( t_{i} \right)}{t_{i+1}-t_{i}}$  $=\frac{S\left( t_{i+1} \right)}{S\left( t_{i} \right)}\cdot\frac{-log\left\{ \frac{S\left( t_{i+1} \right)}{S\left( t_{i} \right)} \right\}}{t_{i+1}-t_{i}}=P\left( T>t_{i+1} \vert T>t_{i} \right)\cdot\frac{\left\{ -logP(T>t_{i+1}\vert T>t_{i}) \right\}}{t_{i+1}-t_{i}}$ |
| **Conditional survival function** | | |
|  | **Method 1** | The probability that the first event does not occur before $\Delta t_{i}=t_{i+1}-t_{i}$, or equivalently, that it occurs after $\Delta t_{i}$ |
|  |  | $S\left( t_{i+1} \vert t_{i} \right)= P\left( T>t_{i+1} \vert T>t_{i} \right)= P\left( T>\left( t_{i+1}-t_{i} \right) \vert T>\left( t_{i}-t_{i} \right) \right)= P\left( T>\Delta t_{i} \vert T>t_{0} \right)=\frac{S\left( \Delta t_{i} \right)}{S\left( t_{0} \right)}=e^{-\int_{t_{0}}^{\Delta t_{i}} h\left( u \right)du}=S(\Delta t_{i})$ |
|  | **Method 2** | The conditional probability that the ${(i+1)}^{th}$ event does not occur by time $t_{i+1}$, given that the $i^{th}$ event occurred at $t_{i}$ |
|  |  | $S\left( t_{i+1} \vert t_{i} \right)= P\left( T>t_{i+1} \vert T>t_{i} \right)=\frac{S\left( t_{i+1} \right)}{S\left( t_{i} \right)}=e^{-\int_{t_{i}}^{t_{i+1}} h\left( u \right)du}=S(t_{i}, t_{i+1})$ |
|  | **Method 3** |  |

| **Conditional hazard function** | | |
| --- | --- | --- |
|  | **Method 1** | The conditional probability that the ${(i+1)}^{th}$ event occurs instantaneously at time $\Delta t_{i}=t_{i+1}-t_{i}$,  given that the ${(i+1)}^{th}$ event has not occurred up to the time $\Delta t_{i}$ |
|  |  | The instantaneous rate at which the ${(i+1)}^{th}$ event occurs at time $\Delta t_{i}=t_{i+1}-t_{i}$, conditional on survival (or event avoidance) up to $\Delta t_{i}$ |
|  |  | $h\left( t_{i+1} \vert t_{i} \right)=\lim_{\delta t\to0} \frac{P\left( (t_{i+1}\leq T<t_{i+1}+\delta t\vert T>t_{i})\vert(T\geq t_{i+1}\vert T>t_{i}) \right)}{\delta t}=\lim_{\delta t\to0} \frac{P\left( (t_{i+1}-t_{i}\leq T<t_{i+1}-t_{i}+\delta t\vert T>t_{i}-t_{i})\vert(T\geq t_{i+1}-t_{i}\vert T>t_{i}-t_{i}) \right)}{\delta t}$  $=\lim_{\delta t\to0} \frac{P\left( (\Delta t_{i}\leq T<\Delta t_{i}+\delta t\vert T>t_{0})\vert(T\geq\Delta t_{i}\vert T>t_{0}) \right)}{\delta t}=\frac{1}{S(\Delta t_{i}\vert t_{0})}\cdot\left\{ \lim_{\delta t\to0} \frac{S(\Delta t_{i}\vert t_{0})-S(\Delta t_{i}+\delta t\vert t_{0})}{\delta t} \right\}=-\frac{S^{'}\left( \Delta t_{i}\vert t_{0} \right)}{S\left( \Delta t_{i}\vert t_{0} \right)}=-\frac{S^{'}\left( \Delta t_{i} \right)}{S\left( \Delta t_{i} \right)}$  $=-\frac{-S\left( \Delta t_{i} \right)\cdot H^{'}\left( \Delta t_{i} \right)}{S\left( \Delta t_{i} \right)}=H^{'}\left( \Delta t_{i} \right)=\frac{d}{dt}\left[ \int_{t_{0}}^{t} h\left( u \right)du \right]_{t=\Delta t_{i}}=\frac{d}{dt}\left[ H\left( t \right) \right]_{t=\Delta t_{i}}= h(\Delta t_{i})$ |
|  | **Method 2** | The conditional probability that the ${(i+1)}^{th}$ event occurs instantaneously at time $t_{i+1}$, given that the ${(i+1)}^{th}$ event has not occurred up to the time $t_{i+1}$ |
|  |  | The instantaneous rate at which the ${(i+1)}^{th}$ event occurs at time $t_{i+1}$, conditional on survival (or event avoidance) up to $t_{i+1}$ |
|  |  | $h\left( t_{i+1}\vert t_{i} \right)=\lim_{\delta t\to0} \frac{P\left( (t_{i+1}\leq T<t_{i+1}+\delta t\vert T>t_{i})\vert(T\geq t_{i+1}\vert T>t_{i}) \right)}{\delta t} =\frac{1}{S\left( t_{i+1} \vert t_{i} \right)}\cdot\left\{ \lim_{\delta t\to0} \frac{S\left( t_{i+1} \vert t_{i} \right)-S\left( t_{i+1}+\delta t \vert t_{i} \right)}{\delta t} \right\}=-\frac{S^{'}\left( t_{i+1} \vert t_{i} \right)}{S\left( t_{i+1} \vert t_{i} \right)}=-\frac{-S\left( t_{i+1} \vert t_{i} \right){\cdot H}^{'}\left( t_{i+1} \vert t_{i} \right)}{S\left( t_{i+1} \vert t_{i} \right)}$  $=H^{'}\left( t_{i+1}\vert t_{i} \right)=H^{'}\left( t_{i}{, t}_{i+1} \right)=\frac{d}{dt}\left[ \int_{t_{i}}^{t} h\left( u \right)du \right]_{t=t_{i+1}}=\frac{d}{dt}\left[ H\left( t \right)-H\left( t_{i} \right) \right]_{t=t_{i+1}}=h\left( t_{i+1} \right)=\lim_{\delta t\to0}\frac{P\left( t_{i+1}\leq T<t_{i+1}+\delta t\vert T\geq t_{i+1} \right)}{\delta t}$  $=\frac{1}{S\left( t_{i+1} \right)}\cdot\left\{ \lim_{\delta t\to0} \frac{S\left( t_{i+1} \right)-S\left( t_{i+1}+\delta t \right)}{\delta t} \right\}=-\frac{S^{'}\left( t_{i+1} \right)}{S\left( t_{i+1} \right)}=-\frac{-S\left( t_{i+1} \right)\cdot H^{'}\left( t_{i+1} \right)}{S\left( t_{i+1} \right)}=H^{'}\left( t_{i+1} \right)=\frac{d}{dt}\left[ \int_{t_{0}}^{t} h\left( u \right)du \right]_{t=t_{i+1}}$ |
|  | **Method 3** | The conditional probability that the ${(i+1)}^{th}$ event occurs instantaneously at time$t_{i+1}$, given that the event has not occurred within the interval $(t_{i}, t_{i+1})$ |
|  |  | The instantaneous rate at which the ${(i+1)}^{th}$ event occurs at time$t_{i+1}$, conditional on survival (or event avoidance) within the interval $(t_{i}, t_{i+1})$ |
|  |  | The average rate of change in cumulative hazard $H(t)$ across the interval ($t_{i}, t_{i+1}]$. |
|  |  | The conditional hazard of the ${(i+1)}^{th}$ event at time $t_{i+1}$, incorporating the hazard of the previous event at $t_{i}$ |
|  |  | $h\left( t_{i+1} \vert t_{i} \right)=\lim_{\delta t\to0} \frac{P\left( (t_{i+1}\leq T<t_{i+1}+\delta t\vert T>t_{i})\vert(T\geq t_{i+1}\vert T>t_{i}) \right)}{\delta t} =\frac{1}{S\left( t_{i+1} \vert t_{i} \right)}\cdot\left\{ \lim_{\delta t\to0} \frac{S\left( t_{i+1} \vert t_{i} \right)-S\left( t_{i+1}+\delta t \vert t_{i} \right)}{\delta t} \right\}=-\frac{S^{'}\left( t_{i+1} \vert t_{i} \right)}{S\left( t_{i+1} \vert t_{i} \right)}=-\frac{-S\left( t_{i+1} \vert t_{i} \right){\cdot H}^{'}\left( t_{i+1} \vert t_{i} \right)}{S\left( t_{i+1} \vert t_{i} \right)}$  $=H^{'}\left( t_{i+1}\vert t_{i} \right)=H^{'}\left( t_{i}, t_{i+1} \right)=\frac{1}{t_{i+1}-t_{i}}\left[ \int_{t_{0}}^{t_{2}} h(u)du-\int_{t_{0}}^{t_{1}} h(u)du \right]_{t_{1}=t_{i}, t_{2}=t_{i+1}}=\frac{\int_{t_{0}}^{t_{i+1}} h(u)du-\int_{t_{0}}^{t_{i}} h(u)du}{t_{i+1}-t_{i}}$  where $t_{1}$and $t_{2}$ are treated as two realized values of an event-time random variable associated with $h(t)$. |

**(b) Comparison of independence, conditioning, and event-process structures across single- and recurrent-event approaches**

| **Approach** | | **Between-subject**  **independence structure** | **Independence/dependence structure**  **of event and interval contributions** | **Conditioning and time-scale structure of**  **survival/hazard functions** | **Event-occurrence assumption**  **following the preceding event** |
| --- | --- | --- | --- | --- | --- |
| **Single-event KM** | | Independent subjects | Not applicable;  One event-time observation per subject  (No recurrent within-subject event structure) | Conditional product-limit factors  ; Marginal survival from baseline  $\hat{S\left( t \right)}=\hat{P}(T>t)$  Pooled discrete event-time hazard increments. | Not applicable  ; Single-event framework |
| **Repeated-event KM** | | Subject-level structure  not explicitly modeled  because of population-level pooling | Conditional independence  given the preceding event time;  Subject-level dependence  is not explicitly modeled  because interval/event information is pooled  at the population level. | Conditional survival $\hat{S(t_{i+1}\mid t_{i})}$  given the preceding event time  Pooled discrete event-time hazard increments. | Event-count process  ; Multiple events possible  following the preceding event |
| **Single-event parametric** | | Independent subjects | Not applicable;  One event-time observation per subject  (No recurrent within-subject event structure) | Unconditional baseline-referenced functions:  $S(t)$, $H(t)$, and $h(t)$ | Not applicable  ; Single-event framework |
| **Repeated-**  **event parametric** | **Method 1** | Independent subjects | Renewal independence  of gap times within subject | Unconditional functions on the reset gap-time:  $S(\Delta t_{i})$, $H(\Delta t_{i})$, and $h(\Delta t_{i})$  The preceding event resets the time origin | Renewal process  ; Single event possible  following the preceding event |
|  | **Method 2** | Independent subjects | Conditional independence  given the preceding event time  on the calendar-time scale within subject | Conditional survival $S(t_{i+1}\mid t_{i})$  given the preceding event time  Unconditional calendar-time hazard $h(t_{i+1})$. | Renewal-type  sequential single-event process  ; Single event possible  following the preceding event |
|  | **Method 3** | Independent subjects | Conditional independence  given the preceding event time  on the calendar-time scale within subject | Conditional survival $S(t_{i+1}\mid t_{i})$  given the preceding event time  Conditional interval-average hazard over $(t_{i},t_{i+1}]$  $h(t_{i+1}\mid t_{i})=\frac{H(t_{i+1})-H(t_{i})}{t_{i+1}-t_{i}}.$ | Event-time process  with interval event intensity  ; Multiple events possible  following the preceding event |

Note: $t_{i}$ denotes the preceding event time and $t_{i+1}$ denotes the current event time. In this table, “unconditional” means that the function is not conditioned on a preceding event time or recurrent-event history. The ordinary hazard $h(t)$ nevertheless retains its standard survival-analysis interpretation as an instantaneous event rate conditional on remaining event-free up to time $t$. In the last column (“Event-occurrence assumption following the preceding event”), $t_{i}$denotes the preceding event time and $t_{i+1}$denotes the current event time in a pair of successive event times. The event-occurrence assumption refers to whether, in defining the probability density or probability mass for the current event at $t_{i+1}$relative to the preceding event at $t_{i}$, the underlying event process assumes the occurrence of a single subsequent event or allows multiple recurrent events following $t_{i}$. “Single event possible following the preceding event” indicates that each probability contribution assumes the occurrence of only one subsequent event after the preceding event. In contrast, “multiple events possible following the preceding event” indicates that the underlying event-count or interval-intensity process allows multiple recurrent events after the preceding event. The former applies to each pair of successive event times and does not mean that only one event can occur during the entire follow-up. Likewise, the latter does not imply that multiple events occur between the two consecutive event times $t_{i}$ and $t_{i+1}$.

**Supplementary 10. Equivalence of conditional and time-shifted survival under constant hazard in method 1** [12]

For the exponential model (constant hazard, $\lambda$) :

$$h\left( t \right)=\lambda=h\left( t_{i+1} \right)=h\left( t_{i+1}-t_{i} \right)=h\left( \Delta t_{i} \right)$$

and

$$H\left( t \right)=\lambda t$$

therefore,

$$S\left( t_{i+1} | t_{i} \right)=\frac{S\left( t_{i+1} \right)}{S\left( t_{i} \right)}=e^{-\int_{t_{i}}^{t_{i+1}} h\left( u \right)du}=e^{\left\{ -\left( H\left( t_{i+1} \right)-H(t_{i}) \right) \right\}}=e^{\left( -\lambda(t_{i+1}-t_{i}) \right)}$$

when the survival values are expressed on the time scale shifted by $t_{i}$

$$e^{\left( -\lambda(t_{i+1}-t_{i}) \right)}=e^{-H(t_{i+1}-t_{i})}=S\left( t_{i+1}-t_{i} \right)=S\left( \Delta t_{i} \right)=S\left( t_{i+1} | t_{i} \right)$$

Accordingly, under the exponential model, the equalities $h\left( t_{i+1} \right)=h\left( \Delta t_{i} \right)$ and $S\left( t_{i+1} | t_{i} \right)=S\left( \Delta t_{i} \right)$ always hold.

**Supplementary 11. Proof that** $\boldsymbol{H'}\left( \boldsymbol{t}_{\boldsymbol{i+1}} | \boldsymbol{t}_{\boldsymbol{i}} \right)\boldsymbol{=H'(}\boldsymbol{t}_{\boldsymbol{i}}\boldsymbol{,}\boldsymbol{t}_{\boldsymbol{i+1}}\boldsymbol{)=}\boldsymbol{h}\left( \boldsymbol{t}_{\boldsymbol{i+1}}\boldsymbol{|}\boldsymbol{t}_{\boldsymbol{i}} \right)$ [10-11, 13, 26-27]

$$H\left( t_{i+1} | t_{i} \right) =\int_{t_{i}}^{t_{i+1}} \lim_{\delta t\to0} \frac{P\left( (t\leq T<t+\delta t|T>t_{i})|(T\geq t|T>t_{i}) \right)}{\delta t}dt$$

$$=\int_{t_{i}}^{t_{i+1}} \frac{1}{S(t|t_{i})}\cdot\left\{ \lim_{\delta t\to0} \frac{S(t|t_{i})-S(t+\delta t|t_{i})}{\delta t} \right\}dt$$

$$=\int_{t_{i}}^{t_{i+1}} \left\{ -\frac{S^{'}\left( t | t_{i} \right)}{S\left( t | t_{i} \right)} \right\}dt=-\left[ logS\left( t | t_{i} \right) \right]_{t_{i}}^{t_{i+1}}=-\left[ logS(t_{i},t) \right]_{t_{i}}^{t_{i+1}}$$

$$=-\left[ loge^{-\int_{t_{i}}^{t} h\left( u \right)du} \right]_{t_{i}}^{t_{i+1}}=-\left[ -\int_{t_{i}}^{t} h\left( u \right)du \right]_{t_{i}}^{t_{i+1}}=\int_{t_{i}}^{t_{i+1}} h\left( u \right)du=H(t_{i},t_{i+1})$$

according to the principle that “when $f(x)$ is identical to $g\left( x \right),$if $f(x)$ and $g(x)$ are continuously differential $\left( C^{1} \right)$ under the condition that their derivatives $f'(x)$ and $g'(x)$ are continuous, then $f'(x)$ is identical to $g'(x)$” and $H'\left( t_{i+1} | t_{i} \right)$ is identical to $H'(t_{i}{,t}_{i+1})$ [26-27].

$$H\left( t_{i+1} | t_{i} \right) =H(t_{i},t_{i+1})$$

$$H'\left( t_{i+1} | t_{i} \right)=H'(t_{i},t_{i+1})=h\left( t_{i+1}|t_{i} \right)$$

**Supplementary 12. Mathematical basis for the approximate VPC agreement between the KM method and method 2 under smoothly varying hazard functions** [13, 17, 21-22]

For the theoretical comparison, the estimate of $\Delta H_{i}$ by both methods is assumed to be identical and denoted as $x$. Additionally, we define the following variables.

$x=\Delta H_{i}$, $\Delta t=t_{i+1}-t_{i}$, $\bar{h}=\frac{x}{\Delta t}$, $r=\frac{h(t_{i+1})}{\bar{h}}$

The KM conditional probability in **Table 1** represents a discrete conditional probability mass associated with the jump at $t_{i+1}$, whereas $f_{k}\left( t_{i+1}\mid t_{i} \right)$ in Equation 7 is a continuous-time conditional density evaluated at the endpoint of the interval $(t_{i}, t_{i+1}]$. Multiplying **Equation 7** by $\Delta t$ provides a right-endpoint local probability approximation that is directly comparable to the nonparametric estimate $\hat{f\left( t_{i+1}|t_{i} \right)}=\Delta H_{i}=x$.

The purpose of this approximation is not to evaluate the exact interval-probability difference between the two methods, but to examine how their local discrepancy changes according to hazard smoothness.

Under sufficiently short intervals and small cumulative hazard increments, and within the decreasing-hazard setting considered here, the ratio $r=h\left( t_{i+1} \right)/\bar{h}$ provides a useful local measure of this smoothness. Accordingly, the following analysis is intended to explain the observed VPC pattern rather than to provide a complete error characterization.

$f_{k}\left( t_{i+1} | t_{i} \right)\cdot\Delta t=S\left( t_{i+1} | t_{i} \right)\cdot h\left( t_{i+1} \right)\cdot\Delta t={exp}^{-H\left( t_{i},t_{i+1} \right)}\cdot h\left( t_{i+1} \right)\cdot\Delta t={exp}^{-x}\cdot rx$[13]

The discrepancy between $f_{k}\left( t_{i+1}\mid t_{i} \right)\cdot\Delta t$ and the KM conditional probability mass in **Table 1** can be formally expressed using a Taylor series [21].

$$\left| \hat{f\left( t_{i+1}|t_{i} \right)}-f_{k}\left( t_{i+1} | t_{i} \right)\cdot\Delta t \right|=\left| x-{exp}^{-x}rx \right|=\left| x-rx(1-x+\frac{x^{2}}{2!}-\cdots) \right|=\left| x(1-r)+rx^{2}-r\frac{x^{3}}{2!}+\cdots\right|$$

where, $x=\Delta H_{i}$ denotes the cumulative hazard increment. In typical clinical settings, the hazard $h(t)$ for disease progression or adverse reactions after treatment typically decreases, and observation intervals $\Delta t$ are chosen sufficiently short [13, 17]. Consequently, the cumulative hazard increment between events, $x=\Delta H_{i}=\bar{h}\cdot\Delta t$, remains small with $x=\Delta H_{i}\ll1$ (in practice, $\Delta H_{i}\leq0.1$). Accordingly, the approximation error can be analyzed using low-order terms [22].

When $r\approx1$—meaning that the hazard at the endpoint $t_{i+1}$ is close to the interval-average hazard $\bar{h}$, reflecting a smoothly varying hazard—and $\left| \frac{(1-r)}{r} \right|<x$, the magnitude of the linear term $x\left( 1-r \right)$ is smaller than the quadratic term $rx^{2}$. Consequently, the approximation error is dominated by the quadratic-order term. If this inequality is not satisfied, the contribution of the $x\left( 1-r \right)$ term is no longer negligible, and the error is dominated by the linear term $x\left( 1-r \right)$, which is of order $O\left( x \right)$ [22].

Conversely, when $r\ll1$—the hazard at the endpoint${(t}_{i+1})$ of the interval $(t_{i},t_{i+1}]$ is much lower than the average hazard $\bar{(h})$ over the interval, under rapidly decreasing hazards, reflecting a steeply varying hazard, the linear term $x(1-r)$, which is of order $O\left( x \right)$, is no longer negligible, and the error is dominated by $O(x)$ [22].

As $\Delta H_{i}$ is typically small in practice (often $\Delta H_{i}\leq0.1$), an increase in $r$ indicates that the endpoint hazard is closer to the interval-average hazard, corresponding to a more smoothly varying hazard over the interval. For a given small $x$, the approximation error in the regime $r\approx1$, whether dominated by the linear term $x\left( 1-r \right)$, which is of order $O\left( x \right)$, or by the quadratic term $rx^{2}$, which is of order $O\left( x^{2} \right)$, remains smaller than the leading linear error $x\left( 1-r \right)$ in the regime $r\ll1$. The latter regime reflects a rapidly varying (steeply decreasing) hazard. Consequently, for a given small cumulative hazard increment $x$, when $\Delta H_{i}\leq0.1$ and the hazards are decreasing, the discrepancy between method 2 and the KM-based nonparametric estimator is larger for $r\ll1$ than for $r\approx1$.

Consequently, when the hazard function decreases smoothly, the local approximation error of method 2 relative to the KM-based nonparametric estimator tends to remain small, supporting closer agreement between the two methods in the VPC. Conversely, when the hazard function decreases abruptly, the local approximation error becomes more pronounced, providing a mathematical explanation for the larger discrepancies observed between method 2 and the KM-based nonparametric estimates.

This analysis assumes a common cumulative hazard increment $x$for the KM method and method 2. In practice, the methods may yield different estimates of $\Delta H_{i}$, and the observed VPC agreement may additionally reflect interval length, contributions accumulated across intervals, parameter estimation, event-time sparsity, and simulation variability. Thus, the present result should be interpreted as a local explanation of the observed VPC pattern rather than a complete comparison of the two methods.

**Supplementary 13. Counting Process Interpretation of the Conditional Hazard Function in method 3** [10-11, 25]

From the perspective of a counting process, the event occurrence process can be represented by $N(t)$, where $N(t)$ denotes the cumulative number of events observed up to time $t$. The intensity function $\lambda(t)$ represents the instantaneous event occurrence rate per unit time and satisfies

$$E[dN(t)]=\lambda(t)\text{ }dt$$

Conditional on the observed event history and the model parameters, the expected number of events occurring within the interval $(t_{i},t_{i+1}]$ is given by

$$E[N(t_{i+1})-N(t_{i})]=\int_{t_{i}}^{t_{i+1}} \lambda(u)\text{ }du$$

Under the conditional counting-process formulation used here, the hazard function $h(t)$ can be interpreted as the intensity function of the counting process, i.e., $\lambda(t)=h(t)$. Therefore,

$$E[N(t_{i+1})-N(t_{i})]=\int_{t_{i}}^{t_{i+1}} h(u)\text{ }du$$

The cumulative hazard function is defined as $H(t)=\int_{0}^{t} h(u)\text{ }du$, implying that the increment in cumulative hazard between two event times corresponds to the expected number of events occurring in that interval:

$$E[N(t_{i+1})-N(t_{i})]=H(t_{i+1})-H(t_{i})$$

Dividing by the interval length yields

$$h\left( t_{i+1} \mid t_{i} \right)=\frac{\Delta H}{\Delta t}=\frac{H\left( t_{i+1} \right)-H\left( t_{i} \right)}{t_{i+1}-t_{i}}=\frac{E\left[ N\left( t_{i+1} \right)-N\left( t_{i} \right) \right]}{t_{i+1}-t_{i}}$$

Thus, the conditional hazard defined in method 3 can be interpreted as the conditional expected number of events per unit time within the interval, i.e., the interval-averaged event intensity.

**Supplementary 14.** **Mathematical basis for the approximate VPC agreement between the KM method and Method 3 under steeply decreasing hazard functions** [13, 17, 21-22, 33]

For the theoretical comparison, we assume that the estimated cumulative hazard increment $\Delta H_{i}$ is identical across methods: thus, $\Delta H_{i}$ is denoted by $x$. Meanwhile, we define the following variables:

$x=\Delta H_{i}$, $\Delta t=t_{i+1}-t_{i}$, $\bar{h}=\frac{x}{\Delta t}$, $r=\frac{h(t_{i+1})}{\bar{h}}$

The KM conditional probability in **Table 1** represents a discrete conditional probability mass associated with the jump at $t_{i+1}$, whereas $f_{k}\left( t_{i+1}\mid t_{i} \right)$ in **Equation 9** is a continuous-time conditional density evaluated at the endpoint of the interval $(t_{i}, t_{i+1}]$. Multiplying **Equation 9** by $\Delta t$ provides a right-endpoint local probability approximation that is directly comparable to the nonparametric estimate $\hat{f\left( t_{i+1} | t_{i} \right)}=\Delta H_{i}=x$.

As in **Supplementary 12**, this approximation is not intended to evaluate the exact interval-probability difference. Rather, it provides a common local scale for comparing the KM method with methods 2 and 3 and for examining why method 3 may retain closer agreement with the KM-based estimator under steeply decreasing hazards.

Because method 3 uses the interval-average hazard $\bar{h}=\Delta H_{i}/\Delta t$, its local probability approximation does not depend on the endpoint-to-average hazard ratio $r$. This provides a basis for examining why method 3 may show closer agreement with the KM-based estimator than method 2 when the hazard decreases steeply.

$$f_{k}\left( t_{i+1} | t_{i} \right)\cdot\Delta t=S\left( t_{i+1} | t_{i} \right)\cdot\frac{\Delta H_{i}}{\Delta t}\cdot\Delta t={exp}^{-\Delta H_{i}}\cdot\Delta H_{i}={exp}^{-x}\cdot x$$

Under the decreasing-hazard setting considered here and sufficiently short observation intervals, $x=\Delta H_{i}$ remains small—in practice, often $\Delta H_{i}\leq0.1$, as assumed for method 2 in **Supplementary 12** [13, 17]. Therefore, the local approximation error can be analyzed using lower-order terms [22].

The discrepancies between the right-endpoint local probability approximations obtained from methods 2 and 3 and the KM-based nonparametric conditional probability mass $\hat{f\left( t_{i+1} | t_{i} \right)}=\Delta H_{i}=x$ are defined as follows:

The discrepancy between method 2 and the KM-based nonparametric estimate is denoted by $E_{m2}$,

$$E_{m2}=\left| \hat{f\left( t_{i+1}|t_{i} \right)}-f_{k}^{(2)}\left( t_{i+1} | t_{i} \right)\cdot\Delta t \right|=\left| x-r{exp}^{-x}x \right|=\left| x(1-r)+rx^{2}-r\frac{x^{3}}{2!}+\cdots\right|[21]$$

Similarly, the discrepancy between method 3 and the KM-based nonparametric estimate is denoted by $E_{m3}$.

$$E_{m3}=\left| \hat{f\left( t_{i+1}|t_{i} \right)}-f_{k}^{(3)}\left( t_{i+1} | t_{i} \right)\cdot\Delta t \right|=\left| x-{exp}^{-x}x \right|=x\left| 1-(1-x+\frac{x^{2}}{2!}-\cdots) \right|=\left| x^{2}-\frac{x^{3}}{2!}+\frac{x^{4}}{3!}+\cdots\right|$$

Because $h\left( t \right)$ is decreasing,

$$h\left( t_{i+1} \right)\leq h\left( u \right), \forall u\in\left( t_{i},t_{i+1} \right]$$

Therefore,

$$\bar{h}=\frac{1}{\Delta t}\int_{t_{i}}^{t_{i+1}} h(u)\text{ }du\geq h(t_{i+1}) [33]$$

Finally,

$$r=\frac{h\left( t_{i+1} \right)}{\bar{h}}\leq1$$

Under a steeply decreasing hazard, $r\ll1$ and hence $1-r\approx1$. In this regime, the Taylor expansions show that the error of method 2 is dominated by the linear term $x\left( 1-r \right)$, whereas the error of method 3 remains dominated by the quadratic term:

$$E_{m2}=x\left( 1-r \right)+O\left( x^{2} \right), E_{m3}=x^{2}+O\left( x^{3} \right).$$

Since $x$remains small while $1-r$ approaches unity,

$$1-r\gg x, \Delta H_{i}=x\leq0.1,$$

and hence

$$E_{m3}\ll E_{m2}$$

For $x>0$, the direction of this comparison is also confirmed by the exact difference between the two local approximation errors:

$$E_{m2}-E_{m3}=xe^{-x}\left( 1-r \right)\geq0,$$

with equality only when $r=1$. Thus, under rapidly and abruptly decreasing hazard patterns, method 3 has a smaller local approximation error than method 2. This provides a mathematical basis for the closer local agreement of method 3 with the KM-based nonparametric estimator under steeply decreasing hazards.

It should be emphasized that the present analysis concerns a single-interval, right-endpoint local probability approximation under a common cumulative hazard increment $x$. In practice, the methods may yield different estimates of $\Delta H_{i}$, and such between-method differences are not represented in this common-$x$ comparison. It is not intended to provide a complete comparison of the overall VPC or global model-fitting performance.

The observed VPC agreement may additionally reflect the full time-course probability structure, the accumulation of contributions across intervals, parameter estimation, the distribution and sparsity of event times, and finite-sample or simulation variability. Accordingly, this analysis provides a local mathematical explanation for the observed VPC pattern rather than a complete characterization of the overall performance of methods 2 and 3.

**References**

1. Collett D. *Modelling survival data in medical research*. 3rd ed. Boca Raton (FL): Chapman & Hall/CRC; 2015.
2. Lim HS. Brief introduction to parametric time-to-event models. *Transl Clin Pharmacol*. 2021;29(1):1–5. doi: 10.12793/tcp.2021.29.e7
3. Bonate PL. *Pharmacokinetic–pharmacodynamic modeling and simulation*. 2nd ed. New York (NY): Springer; 2011.
4. Cook RJ. *The statistical analysis of recurrent events*. New York (NY): Springer; 2007.
5. Amorim LD, Cai J. Modelling recurrent events: A tutorial for analysis in epidemiology. *Int J Epidemiol*. 2015;44(1):324–333. doi: 10.1093/ije/dyu222
6. Ghosh D, Lin DY. Nonparametric analysis of recurrent events and death. *Biometrics*. 2000;56(2):554–562. doi: 10.1111/j.0006-341X.2000.00554.x
7. Rauch G, Kieser M, Binder H, Bayes-Genis A, Jahn-Eimermacher A. Time-to-first-event versus recurrent-event analysis: Points to consider for selecting a meaningful analysis strategy in clinical trials with composite endpoints. *Clin Res Cardiol*. 2018;107(5):437–443. doi: 10.1007/s00392-018-1205-7
8. Kaplan EL, Meier P. Nonparametric estimation from incomplete observations. *J Am Stat Assoc*. 1958;53(282):457–481. Available from: <https://web.stanford.edu/~lutian/coursepdf/KMpaper.pdf>
9. Wang MC, Chang SH. Nonparametric estimation of a recurrent survival function. *J Am Stat Assoc*. 1999;94(447):146–153. doi: 10.1080/01621459.1999.10473831
10. Andersen PK, Borgan Ø, Gill RD, Keiding N. *Statistical models based on counting processes*. New York (NY): Springer-Verlag; 1993.
11. Fleming TR, Harrington DP. *Counting processes and survival analysis*. New York (NY): John Wiley & Sons; 2011.
12. Kalbfleisch JD, Prentice RL. *The statistical analysis of failure time data*. 2nd ed. Hoboken (NJ): John Wiley & Sons; 2002.
13. Kleinbaum DG, Klein M. *Survival analysis: A self-learning text*. New York (NY): Springer; 2012.
14. Nelson W. *Applied life data analysis*. New York (NY): Wiley; 1982.
15. D’Arrigo G. Methods to analyse time-to-event data: The Kaplan–Meier survival curve. *Oxid Med Cell Longev*. 2021;2021:2290120. doi: 10.1155/2021/2290120
16. Greenwood M. The natural duration of cancer. *Rep Public Health Med Subj*. 1926;33:1–26.
17. Lawless JF. *Statistical models and methods for lifetime data*. 2nd ed. Hoboken (NJ): Wiley; 2003.
18. Therneau TM. A package for survival analysis in R (version 3.x) [R vignette]. 2023. Available from: https://cran.r-project.org/web/packages/survival/vignettes/
19. Therneau TM. *survival*: Survival analysis [R package version 3.5-5]. 2023. Available from: https://CRAN.R-project.org/package=survival
20. Therneau TM, Grambsch PM. Ordered multiple events. In: *Modeling survival data: Extending the Cox model*. New York (NY): Springer; 2000. p. 173–219. doi: 10.1007/978-1-4757-3294-8_8
21. OpenStax. Calculus. Volume 2: Taylor and Maclaurin series. Houston (TX): Rice University; 2022. Available from: <https://openstax.org/books/calculus-volume-2/pages/6-3-taylor-and-maclaurin-series>
22. Conrad K. The remainder in Taylor series. Storrs (CT): University of Connecticut; 2010. Available from: <https://kconrad.math.uconn.edu/blurbs/analysis/TaylorRemainder.pdf>
23. Bauer RJ, Beal SL, Sheiner LB, Bockmann AJ. NONMEM user’s guides: Introduction to NONMEM 7.5.1. Gaithersburg (MD): ICON plc; 2022.
24. Lavielle M. Mixed effects models for the population approach. Boca Raton (FL): Chapman & Hall/CRC; 2014.
25. Aalen OO, Borgan Ø, Gjessing HK. Survival and event history analysis: A process point of view. New York (NY): Springer; 2008.
26. Rudin W. Principles of mathematical analysis. 3rd ed. New York (NY): McGraw-Hill; 1976.
27. Apostol TM. Mathematical analysis. 2nd ed. Reading (MA): Addison-Wesley; 1974.
28. Boyd S, Vandenberghe L. Convex optimization. Cambridge (UK): Cambridge University Press; 2004.
29. Pawitan Y. In all likelihood: Statistical modelling and inference using likelihood. Oxford (UK): Oxford University Press; 2001.
30. Leroy F, Dauxois JY, Thophile H, Haramburu F, Tubert-Bitter P. Estimating time-to-onset of adverse drug reactions from spontaneous reporting databases. *BMC Med Res Methodol*. 2014;14:17. doi: 10.1186/1471-2288-14-17
31. Singer JD, Willett JB. *Applied longitudinal data analysis: Modeling change and event occurrence*. Oxford (UK): Oxford University Press; 2003. p. 348.
32. epiR package documentation. Event instantaneous hazard based on Kaplan–Meier survival estimates [R package epiR version 2.0.75]. Available from: <https://search.r-project.org/CRAN/refmans/epiR/html/epi.insthaz.html>
33. Miller SJ. Intermediate and mean value theorems and Taylor series. Williamstown (MA): Williams College; 2016. Available from: <https://web.williams.edu/Mathematics/sjmiller/public_html/BrownClasses/90/MVT_TaylorSeries.pdf>
34. LibreTexts. Concavity and the second derivative. In: *Calculus*. 3rd ed. 2023. Available from: <https://math.libretexts.org/Bookshelves/Calculus/Calculus_3e_%28Apex%29/03%3A_The_Graphical_Behavior_of_Functions/3.04%3A_Concavity_and_the_Second_Derivative>
35. Audenaert KMR, Aujla JS. On norm sub-additivity and super-additivity inequalities for concave and convex functions. *arXiv* [Preprint]. 2010. Available from: https://arxiv.org/abs/1012.2254
36. Rosen KH. *Discrete mathematics and its applications*. 7th ed. New York (NY): McGraw-Hill; 2012.
37. Knopp K. *Theory and application of infinite series*. London (UK): Blackie & Son; 1951.
38. Venables WN, Ripley BD. *Modern applied statistics with S*. 4th ed. New York (NY): Springer; 2002. p. 353–385.
39. NCSS Statistical Software. Distribution (Weibull) fitting. Available from: <https://www.ncss.com/wp-content/themes/ncss/pdf/Procedures/NCSS/Distribution-Weibull-Fitting.pdf>
40. DataCamp. Weibull distribution: How to model time-to-event data. Available from: <https://www.datacamp.com/tutorial/weibull-distribution>. Accessed October 31, 2025.
41. Wong, C.P. et al. (2025) 'Efanesoctocog alfa population pharmacokinetics and repeated time-to-event analysis of bleeds in adults, adolescents, and children with severe hemophilia A', *The Journal of Clinical Pharmacology*, online ahead of print. doi: 10.1002/jcph.70008
42. Garcia, R., Cheng, S., Glassman, F., Sharma, A., De Miguel-Lillo, B., Wiens, M., Johnston, C., Lawo, J.P., Pragst, I., French, J. and Polhamus, D. (2025) 'Population pharmacokinetic/pharmacodynamic and exposure–response modeling of garadacimab in healthy volunteers and patients with hereditary angioedema', *CPT: Pharmacometrics & Systems Pharmacology*, 14(5), pp. 954–963. doi: 10.1002/psp4.70009
43. Goulooze, S.C., Snelder, N. and Garmann, D. (2026) 'Impact of event-related treatment modifications in exposure-response analyses of repeated time-to-event data', *CPT: Pharmacometrics & Systems Pharmacology*, 15(3), e70231. doi: 10.1002/psp4.70231
