# Supplementary material for: The refinement of repeated exact time-to-event modeling: methodological refinements and implementation in NONMEM® —part I
Source: Front Pharmacol. 2026 Sep 14;17:1791423. doi: 10.3389/fphar.2026.1791423 (PMC13617276; doi:10.3389/fphar.2026.1791423)
Supplement: Supplementary file 5 [file Supplementaryfile5.docx]

**Supplementary 15. NONMEM Control Stream: Method 3**

```
$SUBR ADVAN=13 TOL=6

$MODEL
 COMP=(CUMHAZ)

$PK

 IF (NEWIND.LE.1) THEN
  CUMLAST     = 0
  TIME1     = 0
  TIME2     = 0
 ENDIF

 LAMDA	= THETA(1)*EXP(ETA(1))
 GAMMA 	= THETA(2)

 DEL    = 0.00000001

$DES

 HAZ   = 0

 IF(T.GT.0) THEN
  H1     = (GAMMA/LAMDA)*((T+DEL)/LAMDA)**(GAMMA-1)
  H2     = 1 + ((T+DEL)/LAMDA)**GAMMA
  HAZ    = H1/H2
 ENDIF

 DADT(1) = HAZ

$ERROR

 HAZR   = 0

 IF(TIME.GT.0) THEN
  HAZR   = (GAMMA/LAMDA)*((TIME+DEL)/LAMDA)**(GAMMA-1)/(1+((TIME+DEL)/LAMDA)**GAMMA)
 ENDIF

 CUMHAZ = A(1)
 CUMDIF = CUMHAZ - CUMLAST
 SURV 	= EXP(-CUMDIF)
 TIME2  = TIME

 TBE = TIME2 - TIME1

 IF (DV.EQ.0.AND.MDV.EQ.0) THEN
  Y = SURV * (CUMDIF/TBE)
  TIME1   = TIME
  CUMLAST = CUMHAZ
 ELSE
  TIME1   = TIME1
  CUMLAST = CUMLAST
 ENDIF

 IF (DV.EQ.1) THEN
  Y = EXP(-CUMDIF)
 ENDIF

$THETA
 (0, 30)
 (0, 0.4)

$OMEGA 0 FIX

$ESTIMATION PRINT=2 MAXEVALS=9999 METHOD=COND LAPLACE LIKELIHOOD MSFO=RETTE_method3_loglog.MSF

$COV PRINT=E

$TABLE ID TIME DV HAZR CUMLAST CUMHAZ CUMDIF SURV LAMDA GAMMA TBE
       FILE=RETTE_method3_loglog.FIT NOPRINT NOAPPEND ONEHEADER FORMAT=s1PE17.11
```
