# Supplementary material for: The refinement of repeated exact time-to-event modeling: methodological refinements and implementation in NONMEM® —part I
Source: Front Pharmacol. 2026 Sep 14;17:1791423. doi: 10.3389/fphar.2026.1791423 (PMC13617276; doi:10.3389/fphar.2026.1791423)
Supplement: Supplementary file 6 [file Supplementaryfile6.docx]

**Supplementary 16.** **Derivation of the turning point of the log-logistic hazard function and conditions for increasing and decreasing hazard patterns** [13, 28]

To determine the time point at which the hazard function of the log-logistic model attains its maximum, we consider the derivative of $h(t)$ with respect to $t$. Let

$$x=\frac{t}{\lambda}$$

so that the hazard function can be rewritten as

$$h\left( t \right)=\frac{\gamma}{\lambda}\text{ }\frac{x^{\gamma-1}}{1+x^{\gamma}}$$

Since $\gamma/\lambda$ is a constant, the location of the extremum of $h(t)$ is determined by the derivative of

$$g\left( x \right)=\frac{x^{\gamma-1}}{1+x^{\gamma}}$$

Differentiating $g(x)$ yields

$$g^{'}\left( x \right)=\frac{\left( \gamma-1 \right)x^{\gamma-2}\left( 1+x^{\gamma} \right)-\gamma x^{2\gamma-2}}{\left( 1 + x^{\gamma})^{2} \right.}$$

Setting the numerator equal to zero gives

$$\left( \gamma-1 \right)x^{\gamma-2}\left( 1+x^{\gamma} \right)-\gamma x^{2\gamma-2}=0$$

which can be simplified to

$$x^{\gamma-2}\left( \left( \gamma-1 \right)-x^{\gamma} \right)=0$$

For $x>0$, this implies

$$x^{\gamma}=\gamma-1$$

and hence

$$x=(\gamma-1)^{\frac{1}{\gamma}}$$

Substituting back $x=t/\lambda$, the turning point of the hazard function is obtained as

$$t=\lambda(\gamma-1)^{\frac{1}{\gamma}}$$

This result shows that, for $\gamma>1$, the log-logistic hazard function is unimodal, increasing for $t<\lambda(\gamma-1)^{\frac{1}{\gamma}}$ and decreasing for $t>\lambda(\gamma-1)^{\frac{1}{\gamma}}$. In contrast, for $\gamma\leq1$, the hazard function is monotonically decreasing over time.

**Supplementary 17. Formulation of the residual difference between the hazard in method 2 and the conditional hazard in method 3 through a Taylor series expansion** [21-22, 26, 33-34]

When the cumulative hazard function $H\left( t \right)$ is expanded using a Taylor series at the time of the current event $t_{i+1}$, and the previous event time $t_{i}$is substituted in the expansion, the conditional hazard in method 3 can be expressed as the unconditional hazard in method 2 plus a residual term $R\left( t_{i+1} \right)$ [22, 26].

$$h\left( t_{i+1} | t_{i} \right)=R\left( t_{i+1} \right)+h\left( t_{i+1} \right)$$

$$R\left( t_{i+1} \right)=\sum_{n=2}^{\infty} \frac{\left( -1 \right)^{\left( n-1 \right)}}{n!}\cdot h^{\left( n-1 \right)}\left( t_{i+1} \right)\cdot{\Delta t}^{n-1}=-\frac{1}{2!}{\cdot h}^{'}\left( t_{i+1} \right)\cdot\Delta t+\frac{1}{3!}\cdot h^{''}\left( t_{i+1} \right)\cdot{\Delta t}^{2}-\cdots$$

This residual can be either positive or negative, depending on the shape of the cumulative hazard curve. A positive residual implies that the conditional hazard in method 3 exceeds the hazard of method 2, leading to overprediction, while a negative residual implies underprediction.

Because the residual term is expressed as an infinite series, when $\Delta t$ is small; that is, when the event interval is short, typically during the early observation period—the residual is dominated by the leading term, $-\frac{1}{2!}{\cdot h}^{'}\left( t_{i+1} \right)\cdot\Delta t$ [22]. Accordingly, the sign of the residual is determined by the sign of $h^{'}\left( t \right)$, which represents the curvature of $H\left( t \right)$, which is $H''\left( t \right)$ [34]. Specifically, when $h^{'}\left( t \right)=H^{''}\left( t \right)<0$, meaning that $H\left( t \right)$ is concave and the hazard decreases, the residual is positive. Simply, method 3 yields an overprediction relative to method 2. Conversely, when $h^{'}\left( t \right)=H^{''}\left( t \right)>0$, meaning that $H\left( t \right)$ is convex and the hazard increases, the residual is negative.

Specifically, in the early observation period or when inter-event intervals are short, method 3 tends to overestimate the hazard relative to method 2 when the hazard is decreasing, whereas it tends to underestimate the hazard when the hazard is increasing.

In contrast, when $\Delta t$ is large—corresponding to longer event intervals typically observed in the mid-to-late stages—and the hazard function changes only gradually over the interval, $h^{'}\left( t \right)\approx0$ [22, 26]. In this case, the residual remains close to zero, and methods 2 and 3 yield nearly identical results.

A detailed Taylor series expansion underlying this distinction is provided as follows.

Taylor expansion of $H\left( t \right)$ at time $t_{i+1}$

$$H\left( t \right)=H\left( t_{i+1} \right)+h\left( t_{i+1} \right)\cdot(t-t_{i+1})+\frac{1}{2!}{\cdot h}^{'}\left( t_{i+1} \right){\cdot(t-t_{i+1})}^{2}+\frac{1}{3!}\cdot h^{''}\left( t_{i+1} \right)\cdot{(t-t_{i+1})}^{3}+\cdots$$

substituting $t_{i}$ into $t$ in the expanded Taylor series

$$H\left( t_{i} \right)=H\left( t_{i+1} \right)+h\left( t_{i+1} \right)\cdot\left( t_{i}-t_{i+1} \right)+\frac{1}{2!}{\cdot h}^{'}\left( t_{i+1} \right){\cdot\left( t_{i}-t_{i+1} \right)}^{2}+\frac{1}{3!}\cdot h^{''}\left( t_{i+1} \right)\cdot\left( t_{i}-t_{i+1} \right)^{3}+\cdots$$

substituting $-\Delta t$ for $t_{i}-t_{i+1}$

$$H\left( t_{i} \right)=H\left( t_{i+1} \right)+h\left( t_{i+1} \right)\cdot(-\Delta t)+\frac{1}{2!}{\cdot h}^{'}\left( t_{i+1} \right){\cdot(-\Delta t)}^{2}+\frac{1}{3!}\cdot h^{''}\left( t_{i+1} \right)\cdot{(-\Delta t)}^{3}+\cdots$$

rearranging the above equation

$$H\left( t_{i+1} \right)-H\left( t_{i} \right)=-h\left( t_{i+1} \right)\cdot\left( -\Delta t \right)-\frac{1}{2!}{\cdot h}^{'}\left( t_{i+1} \right){\cdot\left( -\Delta t \right)}^{2}-\frac{1}{3!}\cdot h^{''}\left( t_{i+1} \right)\cdot{(-\Delta t)}^{3}+\cdots$$

dividing both sides by $\Delta t$

$$\frac{H\left( t_{i+1} \right)-H\left( t_{i} \right)}{\Delta t}=h\left( t_{i+1} \right)-\frac{1}{2!}{\cdot h}^{'}\left( t_{i+1} \right)\cdot\Delta t+\frac{1}{3!}\cdot h^{''}\left( t_{i+1} \right)\cdot{\Delta t}^{2}-\cdots$$

grouping the terms starting from the second-order Taylor term into a summation form as the residual form

$$R\left( t_{i+1} \right)=\sum_{n=2}^{\infty} \frac{\left( -1 \right)^{\left( n-1 \right)}}{n!}\cdot h^{\left( n-1 \right)}\left( t_{i+1} \right)\cdot{\Delta t}^{n-1}=-\frac{1}{2!}{\cdot h}^{'}\left( t_{i+1} \right)\cdot\Delta t+\frac{1}{3!}\cdot h^{''}\left( t_{i+1} \right)\cdot{\Delta t}^{2}-\cdots$$

$$\frac{H\left( t_{i+1} \right)-H\left( t_{i} \right)}{\Delta t}=h\left( t_{i+1} \right)+\sum_{n=2}^{\infty} \frac{\left( -1 \right)^{\left( n-1 \right)}}{n!}\cdot h^{\left( n-1 \right)}\left( t_{i+1} \right)\cdot{\Delta t}^{n-1}=h\left( t_{i+1} \right)+R(t_{i+1})$$

since $h(t_{i+1})$ represents the hazard in method 2, while $h\left( t_{i+1} | t_{i} \right)$ denotes the conditional hazard in method 3, the relationship between these two hazard forms can be expressed as follows:

$$h\left( t_{i+1} | t_{i} \right)=\frac{\int_{t_{0}}^{t_{i+1}} h\left( u \right)du-\int_{t_{0}}^{t_{i}} h\left( u \right)du}{t_{i+1}-t_{i}}=\frac{H\left( t_{i+1} \right)-H\left( t_{i} \right)}{\Delta t}=h\left( t_{i+1} \right)+R(t_{i+1})$$

therefore, this can be reduced to the difference between the hazard in method 2 and the conditional hazard in method 3.

$$R\left( t_{i+1} \right)=h\left( t_{i+1} | t_{i} \right)-h\left( t_{i+1} \right)$$

$$h\left( t_{i+1} | t_{i} \right)=R\left( t_{i+1} \right)+h\left( t_{i+1} \right)$$

where $R\left( t_{i+1} \right)=\sum_{n=2}^{\infty} \frac{\left( -1 \right)^{\left( n-1 \right)}}{n!}\cdot h^{\left( n-1 \right)}\left( t_{i+1} \right)\cdot{\Delta t}^{n-1}=-\frac{1}{2!}{\cdot h}^{'}\left( t_{i+1} \right)\cdot\Delta t+\frac{1}{3!}\cdot h^{''}\left( t_{i+1} \right)\cdot{\Delta t}^{2}-\cdots$

**Supplementary 18. Comparison of the likelihood functions in the three methods** [1, 4, 26, 29]

The likelihood formulations can be distinguished by how each method incorporates the conditional hazard values.

In method 1, for each event, the previous event time is reset to $t_{0}$, and the corresponding hazard and survival values are calculated. The gap time $g_{i}=t_{i}-t_{i-1}$, defined as the difference between the current and the previous event times, directly determines each probability term [4]. Suppose a total of $n$ events have occurred.

$$L_{1}=\prod_{i=1}^{n} h(g_{i}){exp}^{-H(g_{i})}$$

Therefore, each term depends solely on its own gap time without any overlap or boundary sharing between adjacent intervals. Consequently, the risk accumulated up to the previous event does not influence the hazard of the subsequent event, and no internal boundary coupling arises.

In method 2, the instantaneous hazard at each event time is directly employed. Survival is computed sequentially across intervals, resulting in a “telescoping product” that reduces to a single survival term over the full interval from $t_{0}$ to $t_{n}$ [1, 29].

$$L_{2}=\left\{ \prod_{i=1}^{n} h(t_{i}) \right\}\cdot{exp}^{\left\{ -[H\left( t_{n} \right)-H\left( t_{0} \right) \right\}}$$

The hazard depends solely on the current event time ${(t}_{i})$, with no involvement of the internal boundary ${(t}_{i-1})$. Thus, no internal boundary coupling is introduced by this method.

In method 3, the hazard is expressed as the average rate of increase in the cumulative hazard between successive event times, thereby embedding the risk of the previous event ${(t}_{i-1})$ into the hazard of the current event ${(t}_{i})$ [4, 26]. Survival is evaluated in the same telescoping manner as in method 2, reducing to a single survival term over the full interval from $t_{0}$ to $t_{n}$.

$$L_{3}=\left\{ \prod_{i=1}^{n} \frac{H\left( t_{i} \right)-H\left( t_{i-1} \right)}{t_{i}-t_{i-1}} \right\}\cdot{exp}^{\left\{ -[H\left( t_{n} \right)-H\left( t_{0} \right) \right\}}$$

However, unlike methods 1 and 2, each hazard in this formulation represents a conditional hazard that incorporates both the cumulative hazard increment and the elapsed time between the preceding and current event times. Accordingly, the internal boundary $t_{i-1}$ appears simultaneously in the average-hazard terms for the preceding and current intervals, resulting in boundary coupling between adjacent segments.

**Supplementary 19. Log-Likelihood ordering of three methods under a concave cumulative hazard function** [21-22, 28, 33-34, 36-37]

This derivation is established for the decreasing-hazard setting considered here, in which $H\left( t \right)$ is concave, the decline in $h\left( t \right)$ is more pronounced during the early stage and becomes smaller as time progresses.

To investigate this more formally, we compare the log-likelihood values derived from **Supplementary 10** [1, 4, 26, 29], where the logarithm is applied to the respective likelihood functions for computational convenience.

The gap time $g_{i}=t_{i}-t_{i-1}$ is defined as the difference between the current and the previous event times. And $\bar{h(t_{i})}=\frac{H\left( t_{i} \right)-H\left( t_{i-1} \right)}{t_{i}-t_{i-1}}$ denotes the average rate of change of $H(t)$ over the interval $(t_{i-1}, t_{i}]$.

$$l_{1}=logL_{1}=\sum_{i=1}^{n} logh(g_{i})-\sum_{i=1}^{n} H(g_{i})$$

$$l_{2}=logL_{2}=\sum_{i=1}^{n} logh(t_{i})-\left\{ H\left( t_{n} \right)-H\left( t_{0} \right) \right\}$$

$$l_{3}=logL_{3}=\sum_{i=1}^{n} log\left\{ \frac{H\left( t_{i} \right)-H\left( t_{i-1} \right)}{t_{i}-t_{i-1}} \right\}-\left\{ H\left( t_{n} \right)-H\left( t_{0} \right) \right\}=\sum_{i=1}^{n} log\bar{h(t_{i})}-\left\{ H\left( t_{n} \right)-H\left( t_{0} \right) \right\}$$

---------------------------------------------------------------------------------------------------------------------------------------

when comparing methods 1 and 2, the difference in log-likelihood can be decomposed as follows.

$$l_{1}-l_{2}=\left\{ \sum_{i=1}^{n} logh(g_{i})-\sum_{i=1}^{n} H(g_{i}) \right\}-\left\{ \sum_{i=1}^{n} logh(t_{i})-\left\{ H\left( t_{n} \right)-H\left( t_{0} \right) \right\} \right\}$$

$$=\left\{ \sum_{i=1}^{n} logh\left( g_{i} \right)-\sum_{i=1}^{n} logh(t_{i}) \right\}-\left\{ \sum_{i=1}^{n} H\left( g_{i} \right)-\left\{ H\left( t_{n} \right)-H\left( t_{0} \right) \right\} \right\}$$

if the hazard is decreasing,

$g_{i}\leq t_{i}\Rightarrow h\left( g_{i} \right)\geq h\left( t_{i} \right)\Rightarrow logh\left( g_{i} \right)-logh(t_{i})\geq0$.

when the cumulative hazard function $H(t)$ is concave, the hazard function $h(t)$ is decreasing, with the rate of decrease diminishing over time. Specifically, the decline in hazard is more pronounced in the early stage, whereas for the same time increment the magnitude of decrease becomes smaller as time progresses. In other words, the difference

$$h\left( t_{i-1} \right)-h\left( t_{i} \right)$$

tends to be relatively large in the early phase and comparatively small in the later phase. Under this structure, since $g_{i}=t_{i}-t_{i-1}\leq t_{i}$, the hazard evaluated at $g_{i}$ takes a relatively large value in the early stage, leading to a more pronounced discrepancy between $h(g_{i})$ and $h(t_{i})$ at earlier time points [28, 34].

Consequently, the log-hazard difference $logh\left( g_{i} \right)-logh(t_{i})$ tends to contribute larger positive values in the early stage. Since events are predominantly concentrated in the early phase, these positive differences accumulate to a substantial extent, resulting in a larger overall positive contribution from the first term.

In the next step, without invoking a Taylor approximation, the sign of the second term can be established directly from the “subadditivity of increasing concave functions” [37].

Since $H(t)$ is concave, increasing, and $H(0)=0$, for any positive $g_{i}$,

$$\sum_{i=1}^{n} H\left( g_{i} \right)\geq H\left( \sum_{i=1}^{n} g_{i} \right)=H\left( t_{n}-t_{0} \right)$$

Because each $g_{i}$ represents a positive time interval between successive events, the inequality always holds, regardless of the magnitude of $g_{i}$.

Therefore, the second term is positive when $H(t)$ is concave:

$$\sum_{i=1}^{n} H\left( g_{i} \right)-H\left( t_{n}-t_{0} \right)\geq0$$

To evaluate the magnitude of the second term, we additionally assume that $H\left( t \right)$ is twice differentiable in a neighborhood of $t=0$, with $H\left( 0 \right)=0$, finite $h\left( 0 \right)>0$, and finite $h^{'}\left( 0 \right)<0$. Under this regularity condition, we substitute $H\left( t_{n}-t_{0} \right)$ to $H(\sum_{i=1}^{n} g_{i})$ and apply a “second-order Taylor expansion” around 0 ($H\left( t \right)\approx h\left( 0 \right)t+\frac{1}{2}h'(0)t^{2}$) [21, 33].

$$\sum_{i=1}^{n} H\left( g_{i} \right)-H\left( t_{n}-t_{0} \right)=\sum_{i=1}^{n} H\left( g_{i} \right)-H\left( \sum_{i=1}^{n} g_{i} \right)$$

$$\approx\sum_{i=1}^{n} \left( h\left( 0 \right)g_{i}+\frac{1}{2}h'(0){g_{i}}^{2} \right)-\left( h\left( 0 \right)\sum_{i=1}^{n} g_{i}+\frac{1}{2}h'(0)\left( \sum_{i=1}^{n} g_{i} \right)^{2} \right)=\frac{1}{2}h'(0)\left( \sum_{i=1}^{n} {g_{i}}^{2}-\left( \sum_{i=1}^{n} g_{i} \right)^{2} \right)$$

The second-order Taylor approximation $H(t)\approx h(0)t+\frac{1}{2}h^{'}(0)t^{2}$ is valid when the gap intervals ($\Delta t_{i}=g_{i}$) are sufficiently small, or when the local curvature of the cumulative hazard function is weak (i.e., $h^{'}(0)$ is close to zero), such that higher-order terms become negligible [21-22, 33].

In practice, this condition is typically satisfied for a concave cumulative hazard function $H(t)$, where events are densely concentrated in the early period (small $g_{i}$) and the hazard becomes nearly constant in the later period ($h^{'}(t)\approx0$).

In this context, according to the “expansion formula of a squared sum” [36],

$$\left( \sum_{i=1}^{n} g_{i} \right)^{2}=\sum_{i=1}^{n} {g_{i}}^{2}+2\sum_{i<k}^{n} g_{i}g_{k}$$

since each $g_{i}$ represents the time interval between successive events, $g_{i}>0$ unless two events occur at the same time. Consequently, the cross-product term $\sum_{i<k}^{n} g_{i}g_{k}$ is strictly positive $(\sum_{i<k}^{n} g_{i}g_{k}>0)$.

Moreover, because the cumulative hazard function $H(t)$ is concave, its second derivative satisfies $H^{''}\left( t \right)=h^{'}(t)<0$ [28, 34].

$$\sum_{i=1}^{n} H\left( g_{i} \right)-H\left( t_{n}-t_{0} \right)\approx\frac{1}{2}h^{'}\left( 0 \right)\left( \sum_{i=1}^{n} {g_{i}}^{2}-\left( \sum_{i=1}^{n} g_{i} \right)^{2} \right)=\frac{1}{2}h^{'}\left( 0 \right)\cdot\left\{ -2\sum_{i<k}^{n} g_{i}g_{k} \right\}=-h^{'}\left( 0 \right)\cdot\sum_{i<k}^{n} g_{i}g_{k}\geq0$$

Moreover, using “expansion formula of a squared sum” [36],

$$\sum_{i<k}^{n} g_{i}g_{k}=\frac{1}{2}\left\{ \left( \sum_{i=1}^{n} g_{i} \right)^{2}-\sum_{i=1}^{n} {g_{i}}^{2} \right\}\leq\frac{1}{2}\left( \sum_{i=1}^{n} g_{i} \right)^{2}$$

$-h^{'}\left( 0 \right)\cdot\sum_{i<k}^{n} g_{i}g_{k}$ is a very small positive value.

From this, the second term becomes a small positive value whenever the $\sum_{i} g_{i}$ is small (e.g., when many events are tightly clustered in a short early period).

Under the stated early-clustering conditions, the first term accumulates positive log-hazard differences, whereas the second term remains a small nonnegative quantity. Thus, in the regime considered here, the first term is expected to dominate the second term, yielding $l_{1}-l_{2}>0$.

Consequently, when the cumulative hazard function $H(t)$ is concave (notably in cases when a large proportion of events occur in the early stage), the first term in $l_{1}-l_{2}$ attains a large positive value, whereas the second term takes a nonnegative value of smaller magnitude, implying that the log-likelihood of method 1 is greater than or equal to that of method 2.

Moreover, since the objective function is defined in NONMEM^®^ as −2×log-likelihood, the objective function value of method 2 is necessarily greater than that of method 1.

---------------------------------------------------------------------------------------------------------------------------------------

A similar reasoning applies when comparing methods 1 and 3.

$$l_{1}-l_{3}=\left\{ \sum_{i=1}^{n} logh(g_{i})-\sum_{i=1}^{n} H(g_{i}) \right\}-\left\{ \sum_{i=1}^{n} log\left\{ \frac{H\left( t_{i} \right)-H\left( t_{i-1} \right)}{t_{i}-t_{i-1}} \right\}-\left\{ H\left( t_{n} \right)-H\left( t_{0} \right) \right\} \right\}$$

$$=\left\{ \sum_{i=1}^{n} logh\left( g_{i} \right)-\sum_{i=1}^{n} log\bar{h(t_{i})} \right\}-\left\{ \sum_{i=1}^{n} H\left( g_{i} \right)-\left\{ H\left( t_{n} \right)-H\left( t_{0} \right) \right\} \right\}$$

The second term is identical to that described above and is again a small positive value.

The first term reflects the comparison between the average rate of change of the cumulative hazard from $t_{i-1}$ to $t_{i}$ and the instantaneous hazard at $g_{i}$. For the event-time patterns considered here, the repeated-event intervals are assumed to satisfy $g_{i}\leq t_{i-1}$ for $i=2,\ldots,n$. This means that the gap intervals remain short while $t_{i-1}$ increases with event order. Also, when the hazard function is decreasing, and by the “property of the concave function $H(t)$” [28, 34] and the “mean value theorem” [33], the following inequality holds:

$$g_{i}\leq t_{i-1}\leq t_{i}\Rightarrow h\left( g_{i}=t_{i}-t_{i-1} \right)\geq h\left( t_{i-1} \right)\geq\bar{h\left( t_{i} \right)}\geq h\left( t_{i} \right), i=2,\ldots,n.$$

Consequently, $h(t_{i})$is less than or equal to $\bar{h}(t_{i})$, whereas $h(g_{i})$ is greater than or equal to $\bar{h}(t_{i})$. As discussed in the comparison between methods 1 and 2, when the cumulative hazard function $H(t)$is concave, the hazard function $h(t)$ decreases over time, with the magnitude of the decrease being more pronounced in the early stage. As a result, $logh(g_{i})-log\bar{h\left( t_{i} \right)}$ attains relatively larger positive values during the early phase.

Moreover, when events are predominantly concentrated at earlier time points, these positive differences accumulate, leading to a larger positive contribution from the first term compared to the second term.

Consequently, under a concave cumulative hazard function $H(t)$ (particularly in scenarios when events are predominantly observed at earlier time points), the first term in $l_{1}-l_{3}$ contributes a substantial positive value, while the second term remains nonnegative and relatively small in magnitude, thereby indicating that the log-likelihood of method 1 is no less than that of method 3.

Thus, the log-likelihood of method 1 is greater than that of method 3, and the corresponding objective function value is greater for method 3 than for method 1.

---------------------------------------------------------------------------------------------------------------------------------------

When comparing methods 2 and 3, the second term is identical in both cases and therefore cancels out, leaving only the difference in the first log-hazard term.

$$l_{3}-l_{2}=\left\{ \sum_{i=1}^{n} log\bar{h(t_{i})}-\left\{ H\left( t_{n} \right)-H\left( t_{0} \right) \right\} \right\}-\left\{ \sum_{i=1}^{n} logh(t_{i})-\left\{ H\left( t_{n} \right)-H\left( t_{0} \right) \right\} \right\}$$

$$=\left\{ \sum_{i=1}^{n} log\bar{h\left( t_{i} \right)}-\sum_{i=1}^{n} logh\left( t_{i} \right) \right\}-\left\{ H\left( t_{n} \right)-H\left( t_{0} \right)-\left\{ H\left( t_{n} \right)-H\left( t_{0} \right) \right\} \right\}=\sum_{i=1}^{n} log\bar{h\left( t_{i} \right)}-\sum_{i=1}^{n} logh\left( t_{i} \right)$$

By the property of the concave function $H(t)$ [28, 34] and the mean value theorem [33], this average rate between $t_{i-1}$ and $t_{i}$ can be interpreted as the hazard evaluated at a point slightly earlier than $t_{i}$. When the hazard function is decreasing, the following inequality naturally holds:

$g_{i}\leq t_{i-1}\leq t_{i}\Rightarrow h\left( g_{i}=t_{i}-t_{i-1} \right)\geq h\left( t_{i-1} \right)\geq\bar{h\left( t_{i} \right)}\geq h\left( t_{i} \right)$.

Under a decreasing hazard function (and additionally in settings where events tend to occur at earlier time points), the difference $log\bar{h\left( t_{i} \right)}-logh(t_{i})$ attains a large positive value, rendering $l_{3}-l_{2}$ positive. This implies that the log-likelihood of method 3 exceeds that of method 2. Accordingly, the objective function value is also greater for method 2 than for method 3.

**Supplementary 20. Summary of parametric analysis methods for repeated exact time events.**

| **Event type** | **Type of analysis** | | **Method** | | | | | |
| --- | --- | --- | --- | --- | --- | --- | --- | --- |
| **Exact**  **time event** | **Method** | | **Method 1** | | **Method 2** | | **Method 3** | |
|  | **Def** | **Survival function** | $S\left( t_{i+1} \vert t_{i} \right)=\frac{S\left( \Delta t_{i} \right)}{S\left( t_{0} \right)}=S(\Delta t_{i})$ | | $S\left( t_{i+1} \vert t_{i} \right)=\frac{S\left( t_{i+1} \right)}{S(t_{i})}=S({t_{i},t}_{i+1})$ | | | |
|  |  | **Cumulative**  **hazard function** | $H(t_{i+1}\vert t_{i})=H\left( \Delta t_{i} \right)$ | | $H(t_{i+1}\vert t_{i})=H(t_{i},t_{i+1})$ | | | |
|  |  | **Hazard function** | $h\left( t_{i+1} \vert t_{i} \right)=h(\Delta t_{i})$ | | $h\left( t_{i+1} \vert t_{i} \right)=h\left( t_{i+1} \right)$ | | $h\left( t_{i+1} \vert t_{i} \right)=\frac{\int_{t_{0}}^{t_{i+1}} h(u)du-\int_{t_{0}}^{t_{i}} h(u)du}{t_{i+1}-t_{i}}$ | |
|  | **NM**  **Code** | **$PK** | CUMLAST  TIME1 | = 0  = 0 | CUMLAST | = 0 | CUMLAST  TIME1  TIME2 | = 0  = 0  = 0 |
|  |  | **$DES** | DELT  DADT(1) | = T-TIME1  = HAZ(DELT) | DADT(1) | = HAZ(T) | DADT(1) | = HAZ(T) |
|  |  | **$ERROR**  **define the variables** | CUMHAZ  CUMDIF  SURV  TIME1 | = A(1)  = CUMHAZ-CUMLAST  = EXP(-CUMDIF)  = TIME | CUMHAZ  CUMDIF  SURV | = A(1)  = CUMHAZ-CUMLAST  = EXP(-CUMDIF) | CUMHAZ  CUMDIF  SURV  TIME2  TBE | = A(1)  = CUMHAZ-CUMLAST  = EXP(-CUMDIF)  = TIME  = TIME2 - TIME1 |
|  |  | **$ERROR**  **exact time event** | Y  CUMLAST  TIME1 | = SURV*HAZ  = CUMHAZ  = TIME | Y  CUMLAST | = SURV*HAZ  = CUMHAZ | Y  CUMLAST  TIME1 | = SURV*(CUMDIF/TBE)  = CUMHAZ  = TIME |
|  |  | **$ERROR**  **no event (else)** | CUMLAST  TIME1 | = CUMLAST  = TIME1 | CUMLAST | = CUMLAST | CUMLAST  TIME1 | = CUMLAST  = TIME1 |
|  |  | **$ERROR**  **right-censored event** | Y | = SURV | Y | = SURV | Y | = SURV |

**Abbreviations**: Def, Definition; NM, NONMEM^®^; PK, parameter and intermediate variable definition block; DES, differential equation system block; ERROR, likelihood and observation model block.

- $\Delta t_{i}=t_{i+1}-t_{i}$
- Two time variables were defined in the $PK block: TIME1, which represents the time of the previous event, and TIME2, which denotes the time of the current event. Both variables were initialized to $t_{0}$ for each patient. TIME1 was used in methods 1 and 3, whereas TIME2 was applied only in method 3. Conversely, method 2 did not employ either TIME1 or TIME2.
- The $DES block specifies the assumed hazard function (HAZ). In method 1, DELT is the time variable in the HAZ function, defined as the difference between the current time (T) and TIME1 (the time of the previous event). In contrast, methods 2 and 3 use the current time (T) directly as the time variable in the HAZ function.
- The $ERROR block defines the survival function (SURV) and calculates the event-time likelihood contribution (Y) at each event time
  - Method 1 evaluates HAZ on a gap-time scale reset as $t_{0}$ at the previous event, while CUMHAZ continues to accumulate and CUMDIF extracts the cumulative-hazard increment between successive events. The difference between the cumulative hazard at the current event (CUMHAZ) and that at the previous event (CUMLAST, corresponding to TIME1) is denoted as CUMDIF. For recurrent events, TIME1 and CUMLAST are updated at every event occurrence. SURV is defined using CUMDIF. The likelihood function is expressed as the product of the survival function and the hazard function shifted by the previous event time to $t_{0}$.
  - Method 2 computes the cumulative hazard (CUMHAZ) by integrating the hazard function in a manner analogous to method 1; however, the time variable is taken directly as the actual event times at which discrete events occur. The cumulative hazard evaluated at the time of the previous event is denoted as CUMLAST, whereas that evaluated at the time of the current event is denoted as CUMHAZ. The difference between these two quantities is then defined as CUMDIF and the survival function (SURV) is defined based on CUMDIF. However, HAZ is computed as the instantaneous hazard at each event time without DELT. The likelihood is expressed as the product of the survival function between consecutive events and the instantaneous hazard at the current event.
  - Method 3 introduces an additional variable, TBE, which is defined as TIME2 minus TIME1 and represents the elapsed time between the previous and current events. TBE serves as the denominator in the calculation of the average rate of change in the cumulative hazard. Here, CUMDIF is defined analogously to method 2 as the difference between CUMLAST, corresponding to TIME1 (the time of the previous event), and CUMHAZ, corresponding to TIME2 (the time of the current event), and serves as the numerator in the calculation of the average cumulative hazard rate. SURV is defined in the same manner as in method 2. CUMHAZ evolves continuously over time, whereas CUMLAST is updated to the current CUMHAZ value at each event. The likelihood function is formulated as the product of the survival function from the previous to the current event and the ratio as CUMDIF/TBE.
- In all three methods, for the final right-censored observation, the likelihood value is equivalent to the survival probability.

**Supplementary 21. Summary of two examples of the repeated exact time events.**

| **Event Type** | **EVID** | **EVENT(DV)** | **MDV** |
| --- | --- | --- | --- |
| Start of study (time = 0) | 2 | . | 1 |
| Exact time of event | 0 | 0 | 0 |
| End of study (censored time of follow-up) | 0 | 1 | 0 |

**Abbreviations**: EVID, event identifier; DV, dependent variable; MDV, missing dependent variable indicator.

**Supplementary 22. Monte Carlo Simulation Algorithm for Recurrent Event Generation in NONMEM^®^**

At each time point or interval, $H\left( t \right)$was used to calculate $S\left( t \right)=\exp\left[ -H\left( t \right) \right]$, from which the event probability $1-S\left( t \right)$was derived. Event occurrence was then determined probabilistically by comparing this model-predicted probability with pseudo-random numbers drawn from a uniform distribution $U(0,1)$. Specifically, in the survival-based implementation, an event was declared when the generated random number exceeded the survival probability $S(t)$, corresponding to DV = 0, whereas DV = 1 indicated that no event occurred at that time point. In the probability-based implementation, the event-generation probability used at each discrete simulation time point was defined as $P(t)=h(t)\cdot S(t)$, and event occurrence at each time point was determined through a Bernoulli trial using this probability. This procedure was repeated sequentially across all observation times or intervals for each individual, thereby generating recurrent-event histories that reflect the hazard and event-history structure specified by each method [3, 23].

Accordingly, the simulation can be interpreted as a Monte Carlo approximation in which event occurrence at each time interval is treated as a probabilistic trial, and repeated sampling yields a realization of the recurrent event process. In addition, the continuous-time hazard function was approximated over discrete time intervals, enabling event generation within the NONMEM^®^ simulation framework [3, 24].

**Supplementary 23. VPC of the probability of remaining free of recurrent events in rats among three models**

**Supplementary 24. Hazard-based methodological interpretation of discrepancies between model-based cumulative probabilities and nonparametric estimates in Figure 3** [1, 4, 13, 25-26, 28, 30-33]

By analyzing the discrepancy patterns between the simulation plots and the Kaplan–Meier (KM) curves arising from both the intrinsic characteristics of each model and the methodological distinctions among the three approaches, the following insights can be derived. As the three methods yield identical results under the exponential model, the analysis focused on the Weibull and log-logistic models, examining the associated discrepancy patterns relative to the KM curve in light of the respective model characteristics and methodological features. Before the subsequent discussion, the constant hazard value $3.60\times{10}^{-2}$, estimated from the exponential model that best fits the nonparametric curve of the rat experimental data, is defined and applied as $\lambda_{e}$.

**(a) Method 1**

Method 1 estimates the hazard at the difference in time between the current and previous events [4]. Accordingly, when compared with nonparametric estimators, this method may yield either underprediction or overprediction depending on the form of the underlying hazard function. In this study, both the Weibull and log-logistic models produced estimates of $\gamma$ less than 1, corresponding to decreasing hazard functions [13]. As a result, the initial phase begins with values relatively greater than $\lambda_{e}$, leading to overprediction until the point of intersection with $\lambda_{e}$, after which underprediction emerges.

If method 1 and the nonparametric method are assumed to employ the same hazard estimation approach (instantaneous hazard), the theoretical intersection between $\lambda_{e}$ and the Weibull hazard occurs at 1.57 weeks (**Supplementary 24-A**) [13], while that with the log-logistic hazard occurs at 1.84 weeks (**Supplementary 24-B**) [13]. However, due to the characteristics of method 1, this method repeatedly evaluates the hazard on the reset gap-time scale, thereby placing greater weight on relatively early portions of the Weibull or log-logistic hazard function [4], which are relatively higher than the nonparametric estimates (instantaneous hazard) at the corresponding time points [25, 30-32].

That is, because method 1 continuously relies on the initial hazard estimates of the Weibull or log-logistic models, the decline in hazard appears more gradual than in nonparametric approaches, which use instantaneous hazards on the real-time scale and thus immediately reflect changes in event density [4, 25, 30-32]. As a result, the crossover point in each model is delayed, to approximately 4 weeks rather than the calculated 1.57 and 1.84 weeks for the Weibull hazard and log-logistic hazard, respectively. Beyond this point, the hazard continues to decline, resulting in underprediction.

**(b) Method 2**

According to characteristics of method 2—applying the instantaneous hazard at the event time in a manner analogous to nonparametric estimation—this method produces a hazard curve that closely approximates the nonparametric curve, provided that the hazard does not change abruptly and the selected model is appropriate [1, 4, 25, 30-32]. Consequently, any discrepancies in hazard values or cumulative probabilities can be attributed to differences in the model structure (exponential, Weibull, log-logistic) rather than to limitations in the methodological approach.

In method 2, the Weibull model produced a $\gamma$ estimate slightly greater than 1, yielding an increasing hazard. Accordingly, because the hazard in the Weibull model begins at a relatively low level and increases gradually [13], underprediction persists until the hazard reaches the $\lambda_{e}$. The crossing point between the hazard in the Weibull model and the $\lambda_{e}$ estimate from the exponential model occurs at 6.3 weeks (**Supplementary 24-C**) [13]. Because method 2 and the nonparametric method (instantaneous hazard) employ comparable approaches to hazard estimation, the resulting intersection point is reflected in the VPC [1, 4, 25, 30-32]. After approximately 6.3 weeks, the Weibull hazard exceeds $\lambda_{e}$, and the resulting excess hazard begins to offset the cumulative underprediction accrued during the earlier period. Consequently, the predicted cumulative-probability curve progressively approaches the KM curve, resulting in close agreement during the later observation period.

In the log-logistic model under method 2, the $\gamma$ estimate was greater than 1, producing the characteristic hazard shape of an increase up to a peak followed by a decline [13]. Based on the parameter estimates from the log-logistic model, the time point corresponding to the maximum hazard is calculated as 3.5 weeks. Furthermore, the intersections between the $\lambda_{e}$ and the log-logistic hazard are identified at 1.6 and 8.2 weeks, respectively (**Supplementary 24-D**) [13].

Therefore, the log-logistic model begins with a relatively low initial hazard compared to the $\lambda_{e}$, resulting in underprediction before the first crossover point at 1.6 weeks. The hazard then increases toward its peak at 3.5 weeks, leading to overprediction, which persists until the second crossover point at 8.2 weeks, after which underprediction resumes.

From the perspective of cumulative probability, the underprediction is pronounced initially; however, the subsequent overprediction between 1.6 and 3.5 weeks offsets this discrepancy, yielding close agreement between the nonparametric estimate and the cumulative probability curve around 3.5 weeks. Because the overprediction observed between 3.5 and 8.2 weeks was relatively modest, the cumulative probability curve of the log-logistic model remained closely aligned with the nonparametric estimate beyond 3.5 weeks. Moreover, because the underprediction beyond 8.2 weeks is negligible, the cumulative probability curve exhibits no meaningful deviation from the nonparametric estimates.

**(c) Method 3**

Due to the nature of method 3, which relies on the average hazard during the interval between previous event and current event, rather than the instantaneous hazard at the event time, through application of the mean value theorem, when changes in hazard are gradual and smooth, the nonparametric estimate may fall below or above the model-based hazard depending on the shape of the cumulative hazard [25-26, 30-33].

In contrast to the Weibull model under method 2, the Weibull hazard under method 3 begins at a relatively high level above $\lambda_{e}$ and decreases over time because the estimated $\gamma$ is less than 1 [13]. If method 3 were treated as employing the same instantaneous-hazard formulation as the nonparametric approach, the theoretical intersection point between $\lambda_{e}$ and the Weibull hazard would occur at approximately 5.4 weeks (**Supplementary 24-E**) [13]. Accordingly, the model-based hazard tends to overpredict relative to $\lambda_{e}$ before the theoretical crossing point and to underpredict thereafter. However, under a decreasing hazard pattern, the interval-averaged conditional hazard used in method 3 is greater than the endpoint instantaneous hazard estimated by the nonparametric method [13, 28]. Therefore, the effective transition in the cumulative-probability pattern may be delayed relative to the theoretical instantaneous-hazard crossing at approximately 5.4 weeks [1, 4, 26, 33]. This methodological upward effect reinforces the model-based overprediction during the early period and, after the theoretical crossing, partially offsets the emerging model-based underprediction [1, 4, 26, 33].

Consequently, the simulated cumulative-probability curve for method 3 remains generally above the nonparametric KM curve, with the discrepancy being most pronounced during the early observation period and gradually diminishing over time. The prediction interval begins to overlap substantially with the KM confidence interval at approximately 4–5 weeks; however, this overlap does not necessarily indicate a crossing of the median curves. During the later observation period, the cumulative-probability curve approaches the KM curve, indicating close agreement despite the persistent but diminishing overprediction.

In contrast, the log-logistic model under method 3 produced an estimated $\gamma>1$, resulting in a hazard that initially increased, reached a peak, and subsequently declined [13]. Assuming methodological equivalence between method 3 and the nonparametric approach with respect to instantaneous-hazard estimation, the model-based instantaneous hazard is calculated to reach its peak at approximately 1.5 weeks and to intersect $\lambda_{e}$ at 0.32 and 6.35 weeks, respectively (**Supplementary 24-F**) [13].

Because the model hazard begins below $\lambda_{e}$ and increases gradually, underprediction in the cumulative-probability curve persists until approximately 1.8 weeks. This is later than the first theoretically calculated instantaneous-hazard crossing at 0.32 weeks. Before the hazard reaches its peak, the cumulative hazard is convex; therefore, the interval-averaged conditional hazard used in method 3 is smaller than the corresponding endpoint instantaneous hazard estimated by the nonparametric method [13, 25–26, 28, 30–33]. This methodological downward effect delays the transition from underprediction to overprediction in the cumulative-probability curve.

Owing to the interval-averaging structure of method 3, the peak of the effective interval-averaged conditional hazard is expected to occur slightly later than the calculated instantaneous-hazard peak at approximately 1.5 weeks. In the VPC, overprediction persists after approximately 1.8 weeks, and the discrepancy between the cumulative-probability curve predicted by method 3 and the KM curve is greatest at approximately 3.5 weeks. This maximum discrepancy may be interpreted as the combined result of model-based overprediction accumulated during the initially increasing-hazard period and the methodological upward effect of method 3 that emerges as the hazard passes its peak and begins to decline. Thereafter, the discrepancy between the two curves gradually decreases.

After the log-logistic hazard passes its peak and begins to decline, the cumulative hazard becomes concave, and method 3 yields an interval-averaged conditional hazard greater than the corresponding endpoint instantaneous hazard estimated by the nonparametric method [13, 25–26, 28, 30–33]. Thus, the methodological upward effect of method 3 does not reverse the declining direction of the log-logistic hazard but attenuates the magnitude of the decline in the effective hazard reflected under method 3. Consequently, the previously established overprediction gap in the cumulative-probability curve decreases more slowly, and the second crossing point occurs substantially later than theoretically expected.

Even after the log-logistic hazard falls below $\lambda_{e}$, resulting in model-based underprediction, the methodological upward effect of method 3 partially offsets this underprediction. Consequently, the second intersection between the cumulative-probability curve predicted by method 3 and the KM curve is delayed to approximately 13 weeks rather than occurring near the theoretically calculated second instantaneous-hazard crossing at approximately 6.35 weeks. After approximately 13 weeks, the KM curve and the cumulative-probability curve predicted by method 3 show nearly identical patterns.

**Supplementary 24-A. The time at which the Weibull hazard function crosses the constant hazard of the exponential model in method 1** [13]

As the observed data most closely followed an exponential model with a constant hazard, this model was regarded as the reference hazard function. Under these assumptions, we analytically examined the discrepancy patterns that arise when fitting the Weibull model to data generated under a constant hazard and identified the theoretical time point at which the model's initial overprediction transitions to underprediction.

**reference hazard in the exponential model**

$$h_{e}\left( t \right)=\lambda_{e}=3.60\times{10}^{-2}$$

**comparison of the Weibull model hazard with the exponential model reference hazard**

$$h_{w}\left( t \right)=\gamma_{w}{\lambda_{w}}^{\gamma_{w}}t^{\gamma_{w}-1}, \lambda_{w}=3.92\times{10}^{-2}, \gamma_{w}=0.735$$

**intersection time between reference and the Weibull hazards**

$$h_{e}\left( t \right)=\lambda_{e}=h_{w}\left( t \right)=\gamma_{w}{\lambda_{w}}^{\gamma_{w}}t^{\gamma_{w}-1}$$

$$t=\left( \frac{\lambda_{e}}{\gamma_{w}\cdot{\lambda_{w}}^{\gamma_{w}}} \right)^{\frac{1}{\gamma_{w}-1}}$$

**by substituting the parameter estimates into the equation,**

$$t=\left( \frac{3.62\times{10}^{-2}}{0.735\cdot{(3.92\times{10}^{-2})}^{0.735}} \right)^{\frac{1}{0.735-1}}\approx11.01 \mathrm{days} \approx1.57 \mathrm{weeks}$$

**Supplementary 24-B. The time at which the log-logistic hazard function crosses the constant hazard in the exponential model in method 1** [13]

As the observed data most closely followed an exponential model with a constant hazard, this model was regarded as the reference hazard function. Under these assumptions, we analytically examined the discrepancy patterns that arise when fitting the log-logistic model to data generated under a constant hazard and identified the theoretical time point at which the model's initial overprediction transitions to underprediction.

**reference hazard in the exponential model**

$$h_{e}\left( t \right)=\lambda_{e}=3.60\times{10}^{-2}$$

**comparison of the log-logistic model hazard with the exponential model reference hazard**

$$h_{l}\left( t \right)=\frac{\left( \frac{\gamma_{l}}{\lambda_{l}} \right)\left( \frac{t}{\lambda_{l}} \right)^{\gamma_{l}-1}}{1+\left( \frac{t}{\lambda_{l}} \right)^{\gamma_{l}}}, \lambda_{l}=1.45\times{10}^{1}, \gamma_{l}=0.984$$

**intersection time between reference and log-logistic hazards**

$$h_{e}\left( t \right)=\lambda_{e}=h_{l}\left( t \right)=\frac{\left( \frac{\gamma_{l}}{\lambda_{l}} \right)\left( \frac{t}{\lambda_{l}} \right)^{\gamma_{l}-1}}{1+\left( \frac{t}{\lambda_{l}} \right)^{\gamma_{l}}}$$

$$\lambda_{e}\left( \frac{t}{\lambda_{l}} \right)^{\gamma_{l}}-\left( \frac{\gamma_{l}}{\lambda_{l}} \right)\left( \frac{t}{\lambda_{l}} \right)^{\gamma_{l}-1}+\lambda_{e}=0$$

**by substituting the parameter estimates into the equation,**

$$3.60\times{10}^{-2}\left( \frac{t}{1.45\times{10}^{1}} \right)^{0.984}-\left( \frac{0.984}{1.45\times{10}^{1}} \right)\left( \frac{t}{1.45\times{10}^{1}} \right)^{0.984-1}+3.60\times{10}^{-2}=0$$

$$t\approx12.86 days \approx1.84 weeks$$

**Supplementary 24-C. The time at which the Weibull hazard function crosses the constant hazard in the exponential model in method 2** [13]

As the observed data most closely followed an exponential model with a constant hazard, this model was regarded as the reference hazard function. Under these assumptions, we analytically examined the discrepancy patterns that arise when fitting the Weibull model to data generated under a constant hazard and identified the theoretical time point at which the model's initial underprediction transitions to overprediction.

**reference hazard in the exponential model**

$$h_{e}\left( t \right)=\lambda_{e}=3.60\times{10}^{-2}$$

**comparison of the Weibull model hazard with the exponential model reference hazard**

$$h_{w}\left( t \right)=\gamma_{w}{\lambda_{w}}^{\gamma_{w}}t^{\gamma_{w}-1}, \lambda_{w}=3.36\times{10}^{-2}, \gamma_{w}=1.05$$

**intersection time between reference and the Weibull hazards**

$$h_{e}\left( t \right)=\lambda_{e}=h_{w}\left( t \right)=\gamma_{w}{\lambda_{w}}^{\gamma_{w}}t^{\gamma_{w}-1}$$

$$t=\left( \frac{\lambda_{e}}{\gamma_{w}\cdot{\lambda_{w}}^{\gamma_{w}}} \right)^{\frac{1}{\gamma_{w}-1}}$$

**by substituting the parameter estimates into the equation,**

$$t=\left( \frac{3.60\times{10}^{-2}}{1.05\cdot{(3.36\times{10}^{-2})}^{1.05}} \right)^{\frac{1}{1.05-1}}\approx44.6 \mathrm{days} \approx6.37 \mathrm{weeks}$$

**Supplementary 24-D. The peak time of the log-logistic hazard function, and the intersection time between the log-logistic hazard and the constant hazard from the exponential model in method 2** [13]

**The peak time of the log-logistic hazard function**

The time at which the maximum hazard function is reached is the point at which the derivative is denoted by $h'\left( t \right)=0$. Simply, the peak hazard occurs when the hazard function stops increasing and starts decreasing.

$$\frac{d}{dt}h_{l}\left( t \right)=\frac{d}{dt}\left\{ \frac{\left( \frac{\gamma_{l}}{\lambda_{l}} \right)\left( \frac{t}{\lambda_{l}} \right)^{\gamma_{l}-1}}{1+\left( \frac{t}{\lambda_{l}} \right)^{\gamma_{l}}} \right\}=0$$

$$t=\lambda_{l}{\cdot(\gamma_{l}-1)}^{\frac{1}{\gamma_{l}}}$$

by substituting the parameter estimates into the equation,

$$\lambda_{l}=2.2\times{10}^{1}, \gamma_{l}=2.3$$

$$22\cdot\left( 2.3-1 \right)^{\frac{1}{2.3}}\approx24.66 days=3.5 weeks$$

As the observed data most closely followed an exponential model with a constant hazard, this model was regarded as the reference hazard function. Under these assumptions, we analytically examined the discrepancy patterns that arise when fitting the log-logistic model to data generated under a constant hazard and delineated the theoretical transition points at which the model shifted from initial underprediction to overprediction and subsequently returned to underprediction.

**The intersection time between the log-logistic hazard and the reference hazard under the exponential model**

**reference hazard in the exponential model**

$$h_{e}\left( t \right)=\lambda_{e}=3.60\times{10}^{-2}$$

**comparison of the log-logistic model hazard with the exponential model reference hazard**

$$h_{l}\left( t \right)=\frac{\left( \frac{\gamma_{l}}{\lambda_{l}} \right)\left( \frac{t}{\lambda_{l}} \right)^{\gamma_{l}-1}}{1+\left( \frac{t}{\lambda_{l}} \right)^{\gamma_{l}}}, \lambda_{l}=2.2\times{10}^{1}, \gamma_{l}=2.3$$

**intersection time between reference and the log-logistic hazards**

$$h_{e}\left( t \right)=\lambda_{e}=h_{l}\left( t \right)=\frac{\left( \frac{\gamma_{l}}{\lambda_{l}} \right)\left( \frac{t}{\lambda_{l}} \right)^{\gamma_{l}-1}}{1+\left( \frac{t}{\lambda_{l}} \right)^{\gamma_{l}}}$$

$$\lambda_{e}\left( \frac{t}{\lambda_{l}} \right)^{\gamma_{l}}-\left( \frac{\gamma_{l}}{\lambda_{l}} \right)\left( \frac{t}{\lambda_{l}} \right)^{\gamma_{l}-1}+\lambda_{e}=0$$

**by substituting the parameter estimates into the equation,**

$$3.60\times{10}^{-2}\left( \frac{t}{2.2\times{10}^{1}} \right)^{2.3}-\left( \frac{2.3}{2.2\times{10}^{1}} \right)\left( \frac{t}{2.2\times{10}^{1}} \right)^{2.3-1}+3.60\times{10}^{-2}=0$$

$$t\approx11.25 days, 57.59days \approx1.6weeks, 8.23weeks$$

**Supplementary 24-E. The time at which the Weibull hazard function crosses the constant hazard in the exponential model in method 3** [13]

As the observed data most closely followed an exponential model with a constant hazard, this model was regarded as the reference hazard function. Under these assumptions, we analytically examined the discrepancy patterns that arise when fitting the Weibull model to data generated under a constant hazard and identified the theoretical time point at which the model's initial overprediction transitions to underprediction. However, owing to the methodological characteristics of method 3 under a declining hazard pattern, the model consistently yields higher hazard estimates than the KM curve at corresponding time points, resulting in persistent overprediction throughout the observation period.

**reference hazard in the exponential model**

$$h_{e}\left( t \right)=\lambda_{e}=3.60\times{10}^{-2}$$

**comparison of the Weibull model hazard with the exponential model reference hazard**

$$h_{w}\left( t \right)=\gamma_{w}{\lambda_{w}}^{\gamma_{w}}t^{\gamma_{w}-1}, \lambda_{w}=6.34\times{10}^{-2}, \gamma_{w}=7.24\times{10}^{-1}$$

**intersection time between reference and the Weibull hazards**

$$h_{e}\left( t \right)=\lambda_{e}=h_{w}\left( t \right)=\gamma_{w}{\lambda_{w}}^{\gamma_{w}}t^{\gamma_{w}-1}$$

$$t=\left( \frac{\lambda_{e}}{\gamma_{w}\cdot{\lambda_{w}}^{\gamma_{w}}} \right)^{\frac{1}{\gamma_{w}-1}}$$

**by substituting the parameter estimates into the equation,**

$$t=\left( \frac{3.60\times{10}^{-2}}{(7.24\times{10}^{-1})\cdot{(6.34\times{10}^{-2})}^{7.24\times{10}^{-1}}} \right)^{\frac{1}{7.24\times{10}^{-1}-1}}\approx38.04 \mathrm{days} \approx5.4 \mathrm{weeks}$$

**Supplementary 24-F. The peak time of the log-logistic hazard function, and the intersection time between the log-logistic and the constant hazards from the exponential model in method 3** [13]

**The peak time of the log-logistic hazard function**

The time at which the maximum hazard function is reached is the point where the derivative is denoted by $h'\left( t \right)=0$. Simply, the peak hazard occurs when the hazard function stops increasing and starts decreasing.

$$\frac{d}{dt}h_{l}\left( t \right)=\frac{d}{dt}\left\{ \frac{\left( \frac{\gamma_{l}}{\lambda_{l}} \right)\left( \frac{t}{\lambda_{l}} \right)^{\gamma_{l}-1}}{1+\left( \frac{t}{\lambda_{l}} \right)^{\gamma_{l}}} \right\}=0$$

$$t=\lambda_{l}{\cdot(\gamma_{l}-1)}^{\frac{1}{\gamma_{l}}}$$

by substituting the parameter estimates into the equation,

$$\lambda_{l}=1.25\times{10}^{1}, \gamma_{l}=1.77$$

$$\left( 12.5 \right)\cdot\left( 1.77-1 \right)^{\frac{1}{1.77}}\approx10.78 days=1.5 weeks$$

As the observed data most closely followed an exponential model with a constant hazard, this model was regarded as the reference hazard function. Under these assumptions, we analytically examined the discrepancy patterns that arise when fitting the log-logistic model to data generated under a constant hazard and delineated the theoretical transition points at which the model shifted from initial underprediction to overprediction and subsequently returned to underprediction. However, owing to both the methodological characteristics of method 3 and the property of the log-logistic model, the model consistently yields higher hazard estimates than the KM curve at the delayed first intersection time point, resulting in persistent overprediction after this point.

**The intersection time between the log-logistic hazard and the constant hazard from the exponential model**

**reference hazard under the exponential model**

$$h_{e}\left( t \right)=\lambda_{e}=3.60\times{10}^{-2}$$

**comparison of the log-logistic model hazard with the exponential model reference hazard**

$$h_{l}\left( t \right)=\frac{\left( \frac{\gamma_{l}}{\lambda_{l}} \right)\left( \frac{t}{\lambda_{l}} \right)^{\gamma_{l}-1}}{1+\left( \frac{t}{\lambda_{l}} \right)^{\gamma_{l}}},\lambda_{l}=1.25\times{10}^{1}, \gamma_{l}=1.77$$

**intersection time between reference and the log-logistic hazards**

$$h_{e}\left( t \right)=\lambda_{e}=h_{l}\left( t \right)=\frac{\left( \frac{\gamma_{l}}{\lambda_{l}} \right)\left( \frac{t}{\lambda_{l}} \right)^{\gamma_{l}-1}}{1+\left( \frac{t}{\lambda_{l}} \right)^{\gamma_{l}}}$$

$$\lambda_{e}\left( \frac{t}{\lambda_{l}} \right)^{\gamma_{l}}-\left( \frac{\gamma_{l}}{\lambda_{l}} \right)\left( \frac{t}{\lambda_{l}} \right)^{\gamma_{l}-1}+\lambda_{e}=0$$

**by substituting the parameter estimates into the equation,**

$$3.60\times{10}^{-2}\left( \frac{t}{1.25\times{10}^{1}} \right)^{1.77}-\left( \frac{1.77}{1.25\times{10}^{1}} \right)\left( \frac{t}{1.25\times{10}^{1}} \right)^{1.77-1}+3.60\times{10}^{-2}=0$$

$$t\approx2.24 days, 44.46 days \approx0.32 weeks, 6.35 weeks$$

**Supplementary 25. Methodological interpretation of discrepancies between model-based hazards and nonparametric estimates in Figure 4** [4, 10-11, 13, 17]

By examining the discrepancy patterns between the simulation results and the Kaplan–Meier (KM) curves, arising from both the intrinsic characteristics of each model and the methodological differences among the three approaches, the following insights can be derived. Because all three methods yielded identical results under the exponential model, the analysis focused on the Weibull and log-logistic models, evaluating the associated discrepancy patterns relative to the KM curve in light of their respective model-specific characteristics and methodological features.

**(a) Weibull model**

The parameters $\lambda$ and $\gamma$ were estimated based on the Weibull model presented in **Supplementary 3** [1-2]. In all three methods, $\gamma$ was estimated to be less than 1, indicating a decreasing hazard over time [13]. The rate of decline $(\gamma)$ was steepest in method 1, followed by method 3, and was slowest in method 2 (**Table 10**).

When parameters were estimated based on the Weibull model formulation presented in **Supplementary 3** [1-2], In the Weibull parameterization used here, $\lambda$ acts as an inverse time-scale parameter. For a given or comparable value of $\gamma$, a larger $\lambda$ compresses the time scale and shifts cumulative event accumulation toward earlier times, whereas a smaller $\lambda$ expands the time scale and delays cumulative event accumulation.

The scale parameter $\lambda$ was estimated in the descending order: method 3 > method 2 > method 1 (**Table 10**). Because a larger $\lambda$ compresses the time scale and is associated with earlier event occurrence, method 3 exhibited the fastest event onset, while method 1 exhibited the slowest.

Method 1 yields parameter estimates associated with the most rapid decline in the hazard and the latest event occurrence. Because method 1 resets the time used in each hazard value as the difference between the current and the preceding event times, this method repeatedly applies to the relatively high hazard values observed in the early phase of the Weibull model [4]. As a result, extremely high hazard rates are concentrated in the initial dense event region. To accommodate this pattern, in this setting, method 1 tends to estimate that $\gamma$ is very small, producing a hazard curve that declines sharply from the initial elevated values. Consequently, as observed in Figure 4, the cumulative-probability curve from the mid-phase onward shows substantial underprediction relative to the KM estimate.

Compared with the log-logistic distribution, the survival function of the Weibull model exhibits an exponential (light) tail, such that progressively less probability mass is assigned to event occurrence at later times (**Supplementary 25-A** [13, 17]). Consequently, owing to this inherent property of the Weibull distribution, event occurrences tend to be shifted toward earlier time periods overall, and beyond a certain point, the hazard rate declines sharply. As a result, in the latter phase of the observation period, the model predicts progressively fewer additional events during the later observation period. Ultimately, this adjustment leads to excessive underprediction of hazard in the later phase, thereby amplifying the overall prediction error.

The maximum likelihood estimation (MLE) procedure jointly estimates $\lambda$ and $\gamma$ to capture the overall hazard structure, adjusting for bias in the regions with greater deviation occurrence to maximize the goodness of fit. Accordingly, when both the inherent characteristics of the Weibull model and the methodological properties of method 1 are taken into account, the MLE appears to have ultimately favored an expansion of the time scale, so as to simultaneously accommodate the high hazard observed in the early stage and minimize deviations in the later stage.

As a result, the MLE enhances the explanatory power for the elevated early-stage hazard through a smaller estimate of $\gamma$, while yielding a very small estimate of $\lambda$. Thus, estimating a smaller value of λ effectively stretches the time scale, slowing the apparent rate of event occurrence in the later phase. This temporal dilation lowers the overall fitted hazard level and slows cumulative event accumulation by shifting the fitted event-time distribution toward later times. In this process, as the occurrence of events is effectively delayed, the excessive magnitude of the initial hazard is also indirectly attenuated.

Consequently, among the three methods, method 1 produces the steepest decline in the slope of the cumulative probability curve, resulting in a marked underprediction relative to the KM-plot across the entire follow-up.

Method 2 positions event occurrence times approximately midway between those of methods 1 and 3 under the Weibull model and tends to exhibit the most gradual decline in hazard estimates. Since method 2 uses the instantaneous hazard calculation analogous to the nonparametric approach, this method appears methodologically most comparable to the KM estimates [4, 10-11].

However, while the KM method nonparametrically reflects discrete event-time hazard increments at the population level, method 2 estimates $\lambda$ and $\gamma$ within a parametric framework using individual-level data, resulting in hazard estimates that reflect a smooth, population-average behavior relative to the KM method. As a result, method 2 is less responsive to the sharp initial hazard increase reflected in the KM estimates and tends to slightly underpredict cumulative event probabilities relative to the KM curve. Among the three methods, method 2 yields a comparatively lower fitted hazard during the early period because it is less affected by the methodological elevation of early hazard estimates observed with methods 1 and 3; consequently, a less pronounced subsequent hazard decline is required, consistent with its largest estimated $\gamma$.

Overall, the simulation plot for method 2 aligns more closely with the slope of the lower confidence bound of the KM curve. The underprediction appears to persist at a nearly constant magnitude throughout the observation period, without a pronounced deviation in any specific interval.

Method 2 avoids estimating $\lambda$ at either extreme—neither as small as in method 1, which expands the time scale and redistributes predicted events toward later times, nor as large as in method 3, which accommodates the elevated early-phase event concentration. As a result, method 2 yields an intermediate $\lambda$ value between those obtained from methods 1 and 3.

The close alignment of the cumulative-probability curve for method 2 with the lower confidence bound of the KM curve suggests that, although methodological limitations may contribute to this pattern, the remaining discrepancy is more likely attributable to the intrinsic shape characteristics of the parametric Weibull model than to differences in hazard-estimation methodology, because method 2 and the KM approach employ closely analogous event-time hazard principles.

Method 3 exhibited the earliest onset of event occurrence, with a faster rate of hazard reduction than method 2 and a rate similar to that of method 1. Method 3 estimates hazard based on the average rate of change in cumulative hazard between consecutive events, from the previous event to the current one. Under a decreasing hazard pattern, this approach tends to yield higher hazard estimates than those (instantaneous hazard) derived from the nonparametric method. Accordingly, the methodological upward effect on the fitted early hazard is weaker than that in method 1 but stronger than that in method 2, yielding an intermediate fitted early-hazard level..

Consequently, method 3 yielded a $\gamma$estimate slightly larger than that of method 1 but substantially smaller than that of method 2, corresponding to a hazard decline nearly as steep as that of method 1. As outlined in the “Method” section, when the cumulative hazard rises steeply in intervals characterized by clustered events, the parametric approach of method 3 more effectively reflects the underlying population-level hazard dynamics (non-parametric method). Thus, during the first week, the slightly amplified hazard estimates align more closely with the nonparametric curve.

However, as the number of events decreases in the middle and later stages relative to the initial phase, the hazard amplification effect inherent to method 3 tends to generate a potential hazard-level overprediction. Nevertheless, this tendency is naturally counterbalanced by the structural properties of the Weibull model, which is characterized by an exponential tail that induces a sharp decline in the slope of the cumulative-probability curve during the middle-to-late stages of follow-up.

Through the interaction of these two features, potential hazard-level overprediction during the later phases is effectively offset, resulting in a transition to slight underprediction of the cumulative event probability.

Accordingly, the MLE procedure appears to operate in a manner that prioritizes improving the explanatory power for the elevated hazard observed in the early phase, rather than directly correcting residual deviations in the middle and late stages, which are already partially adjusted by the model’s structural characteristics. As a result, method 3 yields the largest estimated value of $\lambda$ among the three approaches. This corresponds to a compression of the time scale, which increases the overall vertical magnitude of the hazard function and shifts event occurrences toward earlier time points, thereby reinforcing the model’s ability to account for the high early-stage hazard.

Overall, the cumulative probability curve generated by method 3 closely followed the KM curve during the first week but subsequently tended to underpredict. Notably, none of the three methods produced simulation plots that fully aligned with the K–M curve throughout the entire observation period.

**(b) Log-logistic model**

The parameters $\lambda$ and $\gamma$ were estimated based on the log-logistic model presented in **Supplementary 3** [1-2].

As with the Weibull model, the log-logistic model also yielded $\gamma$ estimates less than 1 across all three methods, indicating a decreasing hazard pattern over time [13]. The decline was steepest in method 1, followed by method 3, and most gradual in method 2 (**Table 10**).

When parameters were estimated according to the log-logistic model parameterization described in **Supplementary 3** [1-2], the estimates of the scale parameter $\lambda$ followed the order: method 1 > method 2 > method 3 (**Table 10**). Because a smaller $\lambda$ compresses the time scale and is associated with accelerated event occurrence, method 3 exhibited the earliest event onset, while method 1 exhibited the latest [1, 39-40]. In the log-logistic model, the relative magnitudes of the $\gamma$ estimates across the three methods were consistent with those observed in the Weibull model. However, the order of the $\lambda$ estimates was reversed between methods 1 and 3 (**Table 10**).

Similar to the Weibull model shown in **Figure 4(b)**, method 1 exhibits the steepest reduction in hazard rate in the log-logistic model presented in **Figure 4(c)**. Owing to the nature of method 1, a very small $\gamma$ is estimated to account for the extremely high initial hazard, thereby producing a pattern characterized by a sharp early decline. Conversely, because the pattern is characterized by a high initial hazard followed by a sharp decline, the cumulative-probability curve predicted by method 1 tends to underpredict cumulative event probability relative to the KM curve in the later phase.

The survival function of the Weibull model exhibits an exponential (light) tail, whereas that of the log-logistic model follows a polynomial (heavy) tail. Consequently, the log-logistic model assigns relatively more probability to event occurrence at later times, allowing predicted events to persist throughout the follow-up period (**Supplementary 25-A** [13, 17]). Therefore, because event occurrences are globally delayed and the overall event-time distribution is shifted toward later periods, the hazard does not exhibit a sharply declining interval. This structural property of the log-logistic model may help alleviate the late-stage hazard underprediction commonly observed with method 1 [4].

The MLE procedure jointly adjusts $\lambda$ and $\gamma$ to achieve an optimal fit for the overall hazard structure. The log-logistic model structurally mitigates the late-stage deviations that may arise under method 1. In a similar manner, the MLE tends to estimate the parameters in a manner that simultaneously attenuates the elevated hazard observed in the early stage while alleviating hazard underestimation in the later stage [13, 17]. Specifically, to offset the high early-stage hazard, the MLE favors a relatively large estimate of $\lambda$, which expands the time scale, reduces the overall vertical magnitude of the hazard function, and delays the timing of event occurrences. This delay in event occurrence, in turn, promotes sustained event incidence in the later stage, thereby indirectly mitigating the underprediction of the late-stage hazard.

Overall, among the three methods, method 1 exhibits the steepest decline in the slope of the cumulative probability curve, resulting in persistent underprediction relative to the KM curve. Because the Weibull and log-logistic models use different parameterizations and units for $\lambda$, their raw estimates are not directly comparable. Based on the model formulations presented in **Supplementary 3** [1–2], the reciprocal of the Weibull $\lambda$was therefore used to express both parameters on a comparable time scale.

Under this transformation, the reciprocal of the Weibull $\lambda$ estimated under method 1 (10,000 h; **Table 10**) is substantially larger than the corresponding $\lambda$ estimate of the log-logistic model (417 h) (**Table 10**). Because larger values of the transformed $\lambda$ indicate an expanded time scale, analogous to the interpretation in the log-logistic model, this implies that under method 1 the Weibull model attenuates the elevated early-stage hazard more strongly than the log-logistic model. Consequently, the underprediction in the early stage is more pronounced for the Weibull model, whereas the extent of underprediction is relatively less severe in the log-logistic model.

In the log-logistic model, similar to the Weibull model, model 2 estimated the event occurrence times that were positioned at an intermediate position between those of methods 1 and 3, exhibiting the most gradual decline in hazard. This method adopts the instantaneous hazard estimation framework analogous to that underlying the KM approach; however, because it iteratively updates model parameters using individual-level data rather than aggregating risks at the population level, it tends to represent the hazard in a more population-averaged and smoothed manner [4, 10-11].

Consequently, it tends to slightly underpredict cumulative event probability relative to the KM curve during the densely clustered early-event period. Accordingly, method 2 yields lower initial hazard estimates than the other methods and is associated with the slowest hazard decline rate and the largest estimated $\gamma$ value.

The survival function of the Weibull model follows an exponential form, imposing a relatively rigid structure that limits curvature changes during the early phase. In contrast, the survival function of the log-logistic model includes a polynomial term in the denominator, allowing a trajectory characterized by a steep initial drop followed by a gradual flattening (**Supplementary 25-A** [13, 17**]**).

Consequently, under the log-logistic model, the cumulative hazard curve can exhibit greater curvature at early stages. In other words, the structurally more flexible nature of the log-logistic model may permit a more sensitive response to densely occurring early events. This flexibility compensates for the slight early underprediction observed in method 2, resulting in an initial segment that more closely approximates the KM curve, particularly within the first week.

After the first week, the cumulative probability curve from method 2 followed a trajectory similar to the slope of the lower bound of the 95% confidence interval (CI) for the KM estimates, akin to the Weibull model. Accordingly, no substantial over- or underprediction requiring hazard adjustment was observed during the first week (unlike method 1).

Thereafter, a stable degree of underprediction persisted, with the predicted curve remaining marginally below the lower confidence bound of the KM estimates. Consequently, as in the Weibull model, only minimal hazard adjustment was necessary, and method 2 estimated a $\lambda$ value at an intermediate level between those obtained from methods 1 and 3.

Consistent with method 1, a comparison under method 2 between the reciprocal of the Weibull $\lambda$ (724.64 h) and the $\lambda$ estimate of the log-logistic model (214 h) indicates that the transformed Weibull $\lambda$ remains substantially larger (**Table 10**). Similarly, because the Weibull model expands the time scale to a greater extent than the log-logistic model, it can be interpreted as exerting a stronger attenuating effect on the elevated hazard observed in the early stage. As a result, the cumulative-probability curve derived from method 2 under the log-logistic model exhibited less pronounced underprediction during the early phase than that under the Weibull model. This finding suggests that the greater survival-function flexibility of the log-logistic model may have mitigated the early-phase underprediction observed with the Weibull model.

Method 3 yielded the earliest estimated event occurrence time and exhibited a faster rate of hazard reduction than method 2, though slightly slower than method 1. Owing to the methodological characteristics of method 3, this method was associated with mildly higher hazard estimates than the instantaneous hazards (nonparametric estimation) observed at each event time under a decreasing hazard pattern.

Accordingly, the initial hazard level estimated by method 3 was intermediate among the three methods—positioned between the extremely high estimate of method 1 and the slightly underpredicted value of method 2. Consequently, method 3 yielded the $\gamma$ estimate slightly larger than that of method 1 but substantially smaller than that of method 2, corresponding to a hazard decline nearly as steep as that of method 1.

The log-logistic model, which incorporates a polynomial structure in the denominator, allows for a steeper initial ascent in the cumulative-probability curve—representing the leading segment of the characteristic flexible trajectory [13, 17]. Through this property, prediction by method 2 exhibited an excellent fit with the KM curve during the first week, when events were densely concentrated, thereby helping to compensate for the minor underprediction exhibited by this method in the early phase [4, 10-11]. In contrast, method 3 showed slight hazard-level overprediction during the early phase. This behavior arises from the combined effects of the flexible structural curvature of the log-logistic model and the methodological hazard amplification, which utilizes the mean rate of change in the cumulative hazard across successive events, inherent to method 3 under a decreasing hazard pattern.

Owing to the structural characteristics of the log-logistic model, a larger initial curvature—corresponding to a steeper early decline in hazard—tends to be followed by a more gradual tailing pattern in the subsequent period [13, 17]. Accordingly, method 3 generates a cumulative probability curve with the sharpest curvature during the first week, followed by gradual flattening thereafter, consistent with a more gradual late-stage decline in the fitted hazard.

Due to the hazard-amplifying effect inherent in method 3 and the relatively gradual decline in hazard during the middle and later stages induced by the polynomial tail of the log-logistic model, a tendency toward risk overprediction may arise in the mid-to-late observation period.

Accordingly, the MLE procedure appears to prioritize mitigating the persistent hazard-level overprediction in the middle and later stages—beyond the inflection point where the curvature of the log-logistic hazard changes—rather than correcting the elevated early-stage hazard briefly observed during approximately the first week. To this end, the MLE appears to have favored a relatively smaller estimate of $\lambda$, resulting in a compressed time scale, an increased overall vertical magnitude of the hazard function, and a shift in event occurrence toward earlier time points. These fitted changes appear to have contributed to a faster overall decline in the hazard, thereby helping to reduce overprediction in the middle and later stages while simultaneously enhancing the model’s explanatory power for the elevated hazard in the early stage. As a result, method 3 yielded the smallest estimated $\lambda$ among the three approaches.

The cumulative probability curve of method 3 slightly overpredicted the KM curve during the first week but remained aligned with the KM trend until approximately week five. Thereafter, the cumulative probability curve exhibited a minor underprediction and remained within the range between the median and the lower bound of the KM curve.

Under method 3, a comparison between the reciprocal of the Weibull $\lambda$ (341.30 h) and the $\lambda$ estimate of the log-logistic model (73.3 h) indicates that the transformed Weibull $\lambda$ is substantially larger (**Table 10**). Consistent with the findings from methods 1 and 2, the Weibull model induces a greater expansion of the time scale than the log-logistic model and can therefore be interpreted as exerting a stronger attenuating effect on the elevated hazard observed in the early stage. Consequently, the cumulative probability curve derived under method 3 closely aligns with the nonparametric estimate up to approximately the first week when using the Weibull model, whereas under the log-logistic model the cumulative probability curve obtained from method 3 exhibits overprediction during the early observation period of roughly one week.

Across all methods, the estimation of the shape parameter $\gamma$ in both the Weibull and log-logistic models appears to have been strongly influenced by the methodological tendency to align the early-phase hazard with the nonparametric estimates, particularly in periods characterized by dense event clustering. As a result, the estimated $\gamma$ values in all approaches may primarily reflect the elevated hazard observed in the initial phase rather than the later-stage dynamics.

In contrast, the estimation of the scale parameter $\lambda$ across methods was largely influenced by the tail behavior inherent to each parametric model. Given its exponential (light-tailed) nature together with the fitted $\gamma<1$ [13, 17], the Weibull model inherently induces a rapid decline in hazard during the middle and later stages. Consequently, the estimated $\lambda$ may reflect a compromise between mitigating middle- and late-stage underprediction and accommodating hazard-level overprediction arising from the methodological characteristics of each approach.

For the log-logistic model, which exhibits a polynomial (heavy) tail [13, 17], the hazard in the middle and late stages declines more gradually, while the early phase is characterized by pronounced curvature. Accordingly, the estimated $\lambda$ may reflect a compromise between leveraging the polynomial tail to reduce method-induced underprediction and restraining potential middle- and late-stage overprediction associated with the model’s structural characteristics.

**Supplementary 25-A. Comparison of the tail behavior of the cumulative hazard and survival functions in the Weibull and log-logistic models** [13, 17]

**Weibull model**

cumulative hazard function

$$H_{w}\left( t \right)=\int_{0}^{t} \gamma({\lambda u)}^{\gamma-1}\lambda du=({\lambda t)}^{\gamma}$$

survival function

$$S_{w}\left( t \right)={exp}^{-({\lambda t)}^{\gamma}}$$

tail behavior $(t\to\infty)$

$$T_{w}\left( t \right)=\lim_{t\to\infty} S_{w}\left( t \right)=\lim_{t\to\infty} {exp}^{-({\lambda t)}^{\gamma}}\to0$$

The survival probability approaches zero at an exponential-type rate, assigning comparatively little probability to very late event times. Accordingly, predicted events become increasingly unlikely during the later follow-up period.

**Log-logistic model**

cumulative hazard function

$$H_{l}\left( t \right)=\int_{0}^{t} \frac{\left( \frac{\gamma}{\lambda} \right)\left( \frac{u}{\lambda} \right)^{\gamma-1}}{1+\left( \frac{u}{\lambda} \right)^{\gamma}}du=log\left\{ 1+\left( \frac{t}{\lambda} \right)^{\gamma} \right\}$$

survival function

$$S_{l}\left( t \right)=\frac{1}{1+({\frac{t}{\lambda})}^{\gamma}}$$

tail behavior $(t\to\infty)$

$$T_{l}\left( t \right)=\lim_{t\to\infty} S_{l}\left( t \right)=\lim_{t\to\infty} \frac{1}{1+({\frac{t}{\lambda})}^{\gamma}}\approx\lim_{t\to\infty} ({\frac{t}{\lambda})}^{-\gamma}\to0$$

The survival probability approaches zero at a polynomial rate. Consequently, the log-logistic model assigns relatively more probability to very late event times than the Weibull model, allowing predicted events to persist further into the follow-up period.

**Supplementary 26. VPC of the probability of remaining free of recurrent adverse events in patients among three models**

**Supplementary 27. VPC of the cumulative hazard of recurrent adverse events in patients among three models**

**References**

1. Collett D. *Modelling survival data in medical research*. 3rd ed. Boca Raton (FL): Chapman & Hall/CRC; 2015.
2. Lim HS. Brief introduction to parametric time-to-event models. *Transl Clin Pharmacol*. 2021;29(1):1–5. doi: 10.12793/tcp.2021.29.e7
3. Bonate PL. *Pharmacokinetic–pharmacodynamic modeling and simulation*. 2nd ed. New York (NY): Springer; 2011.
4. Cook RJ. *The statistical analysis of recurrent events*. New York (NY): Springer; 2007.
5. Amorim LD, Cai J. Modelling recurrent events: A tutorial for analysis in epidemiology. *Int J Epidemiol*. 2015;44(1):324–333. doi: 10.1093/ije/dyu222
6. Ghosh D, Lin DY. Nonparametric analysis of recurrent events and death. *Biometrics*. 2000;56(2):554–562. doi: 10.1111/j.0006-341X.2000.00554.x
7. Rauch G, Kieser M, Binder H, Bayes-Genis A, Jahn-Eimermacher A. Time-to-first-event versus recurrent-event analysis: Points to consider for selecting a meaningful analysis strategy in clinical trials with composite endpoints. *Clin Res Cardiol*. 2018;107(5):437–443. doi: 10.1007/s00392-018-1205-7
8. Kaplan EL, Meier P. Nonparametric estimation from incomplete observations. *J Am Stat Assoc*. 1958;53(282):457–481. Available from: <https://web.stanford.edu/~lutian/coursepdf/KMpaper.pdf>
9. Wang MC, Chang SH. Nonparametric estimation of a recurrent survival function. *J Am Stat Assoc*. 1999;94(447):146–153. doi: 10.1080/01621459.1999.10473831
10. Andersen PK, Borgan Ø, Gill RD, Keiding N. *Statistical models based on counting processes*. New York (NY): Springer-Verlag; 1993.
11. Fleming TR, Harrington DP. *Counting processes and survival analysis*. New York (NY): John Wiley & Sons; 2011.
12. Kalbfleisch JD, Prentice RL. *The statistical analysis of failure time data*. 2nd ed. Hoboken (NJ): John Wiley & Sons; 2002.
13. Kleinbaum DG, Klein M. *Survival analysis: A self-learning text*. New York (NY): Springer; 2012.
14. Nelson W. *Applied life data analysis*. New York (NY): Wiley; 1982.
15. D’Arrigo G. Methods to analyse time-to-event data: The Kaplan–Meier survival curve. *Oxid Med Cell Longev*. 2021;2021:2290120. doi: 10.1155/2021/2290120
16. Greenwood M. The natural duration of cancer. *Rep Public Health Med Subj*. 1926;33:1–26.
17. Lawless JF. *Statistical models and methods for lifetime data*. 2nd ed. Hoboken (NJ): Wiley; 2003.
18. Therneau TM. A package for survival analysis in R (version 3.x) [R vignette]. 2023. Available from: https://cran.r-project.org/web/packages/survival/vignettes/
19. Therneau TM. *survival*: Survival analysis [R package version 3.5-5]. 2023. Available from: https://CRAN.R-project.org/package=survival
20. Therneau TM, Grambsch PM. Ordered multiple events. In: *Modeling survival data: Extending the Cox model*. New York (NY): Springer; 2000. p. 173–219. doi: 10.1007/978-1-4757-3294-8_8
21. OpenStax. Calculus. Volume 2: Taylor and Maclaurin series. Houston (TX): Rice University; 2022. Available from: [https://openstax.org/books/calculus-volume-2/pages/6-3-taylor-and-maclaurin-series](https://openstax.org/books/calculus-volume-2/pages/6-3-taylor-and-maclaurin-series)
22. Conrad K. The remainder in Taylor series. Storrs (CT): University of Connecticut; 2010. Available from: [https://kconrad.math.uconn.edu/blurbs/analysis/TaylorRemainder.pdf](https://kconrad.math.uconn.edu/blurbs/analysis/TaylorRemainder.pdf)
23. Bauer RJ, Beal SL, Sheiner LB, Bockmann AJ. NONMEM user’s guides: Introduction to NONMEM 7.5.1. Gaithersburg (MD): ICON plc; 2022.
24. Lavielle M. Mixed effects models for the population approach. Boca Raton (FL): Chapman & Hall/CRC; 2014.
25. Aalen OO, Borgan Ø, Gjessing HK. Survival and event history analysis: A process point of view. New York (NY): Springer; 2008.
26. Rudin W. Principles of mathematical analysis. 3rd ed. New York (NY): McGraw-Hill; 1976.
27. Apostol TM. Mathematical analysis. 2nd ed. Reading (MA): Addison-Wesley; 1974.
28. Boyd S, Vandenberghe L. Convex optimization. Cambridge (UK): Cambridge University Press; 2004.
29. Pawitan Y. In all likelihood: Statistical modelling and inference using likelihood. Oxford (UK): Oxford University Press; 2001.
30. Leroy F, Dauxois JY, Thophile H, Haramburu F, Tubert-Bitter P. Estimating time-to-onset of adverse drug reactions from spontaneous reporting databases. *BMC Med Res Methodol*. 2014;14:17. doi: 10.1186/1471-2288-14-17
31. Singer JD, Willett JB. *Applied longitudinal data analysis: Modeling change and event occurrence*. Oxford (UK): Oxford University Press; 2003. p. 348.
32. epiR package documentation. Event instantaneous hazard based on Kaplan–Meier survival estimates [R package epiR version 2.0.75]. Available from: <https://search.r-project.org/CRAN/refmans/epiR/html/epi.insthaz.html>
33. Miller SJ. Intermediate and mean value theorems and Taylor series. Williamstown (MA): Williams College; 2016. Available from: <https://web.williams.edu/Mathematics/sjmiller/public_html/BrownClasses/90/MVT_TaylorSeries.pdf>
34. LibreTexts. Concavity and the second derivative. In: *Calculus*. 3rd ed. 2023. Available from: <https://math.libretexts.org/Bookshelves/Calculus/Calculus_3e_%28Apex%29/03%3A_The_Graphical_Behavior_of_Functions/3.04%3A_Concavity_and_the_Second_Derivative>
35. Audenaert KMR, Aujla JS. On norm sub-additivity and super-additivity inequalities for concave and convex functions. *arXiv* [Preprint]. 2010. Available from: https://arxiv.org/abs/1012.2254
36. Rosen KH. *Discrete mathematics and its applications*. 7th ed. New York (NY): McGraw-Hill; 2012.
37. Knopp K. *Theory and application of infinite series*. London (UK): Blackie & Son; 1951.
38. Venables WN, Ripley BD. *Modern applied statistics with S*. 4th ed. New York (NY): Springer; 2002. p. 353–385.
39. NCSS Statistical Software. Distribution (Weibull) fitting. Available from: <https://www.ncss.com/wp-content/themes/ncss/pdf/Procedures/NCSS/Distribution-Weibull-Fitting.pdf>
40. DataCamp. Weibull distribution: How to model time-to-event data. Available from: <https://www.datacamp.com/tutorial/weibull-distribution>. Accessed October 31, 2025.
41. Wong, C.P. et al. (2025) 'Efanesoctocog alfa population pharmacokinetics and repeated time-to-event analysis of bleeds in adults, adolescents, and children with severe hemophilia A', *The Journal of Clinical Pharmacology*, online ahead of print. doi: 10.1002/jcph.70008
42. Garcia, R., Cheng, S., Glassman, F., Sharma, A., De Miguel-Lillo, B., Wiens, M., Johnston, C., Lawo, J.P., Pragst, I., French, J. and Polhamus, D. (2025) 'Population pharmacokinetic/pharmacodynamic and exposure–response modeling of garadacimab in healthy volunteers and patients with hereditary angioedema', *CPT: Pharmacometrics & Systems Pharmacology*, 14(5), pp. 954–963. doi: 10.1002/psp4.70009
43. Goulooze, S.C., Snelder, N. and Garmann, D. (2026) 'Impact of event-related treatment modifications in exposure-response analyses of repeated time-to-event data', *CPT: Pharmacometrics & Systems Pharmacology*, 15(3), e70231. doi: 10.1002/psp4.70231
